# Capsid and integrase play essential apposing roles in viral ribonucleoprotein assembly during HIV-1 core morphogenesis

**DOI:** 10.1016/j.isci.2026.116378

**Published:** 2026-06-22

**Authors:** Ahinsa Ranaweera, Jonathan R. Andino-Moncada, Sarah E. Dillon, Satya P. Singh, Scott M. Stagg, Alan N. Engelman, Christopher Aiken, Ashwanth C. Francis

**Affiliations:** 1 Institute of Molecular Biophysics and Department of Biological Sciences, Florida State University, Tallahassee, FL, USA; 2 Department of Cancer Immunology and Virology, Dana-Farber Cancer Institute, Boston, MA, USA; 3 Department of Medicine, Harvard Medical School, Boston, MA, USA; 4 Department of Pathology, Microbiology and Immunology, Vanderbilt University Medical Center, Nashville, TN, USA

**Keywords:** virology

## Abstract

HIV-1 virions harbor a core composed of a conical shell of capsid proteins that encases the viral ribonucleoprotein complex (vRNP). The vRNP is composed of HIV-1 genomic RNA (gRNA) and nucleocapsid (NC), reverse transcriptase (RT), and integrase (IN) proteins. Using a rapid affinity-capture purification approach, we have enriched for native HIV-1 capsids and analyzed the roles of IN-gRNA interactions and capsid lattice assembly on HIV-1 core formation. Following disruption of IN-gRNA interactions by allosteric IN inhibitor treatment or IN-deletion, we find that RT remains associated with cores that have effectively lost their IN, gRNA, and NC contents. Morphologically disrupted capsids formed by clinical inhibitor lenacapavir treatment or capsid hexamer cross-linking, by contrast, retained IN-gRNA but lost their RT and NC contents. These contrasting effects reveal that IN and the assembling capsid architecture cooperatively regulate the formation and encapsulation of vRNPs into the viral core during infectious HIV-1 morphogenesis.

## Highlights

• HIV-1 RNA, NC, IN, and RT incorporate into capsid cores through distinct pathways
• IN mediates gRNA incorporation into conical and non-canonical hexameric capsids
• RT incorporates into capsids independent of gRNA, IN, and NC
• NC and RT incorporation depends on canonical capsid assembly pathways

## Graphical abstract

**Figure:**
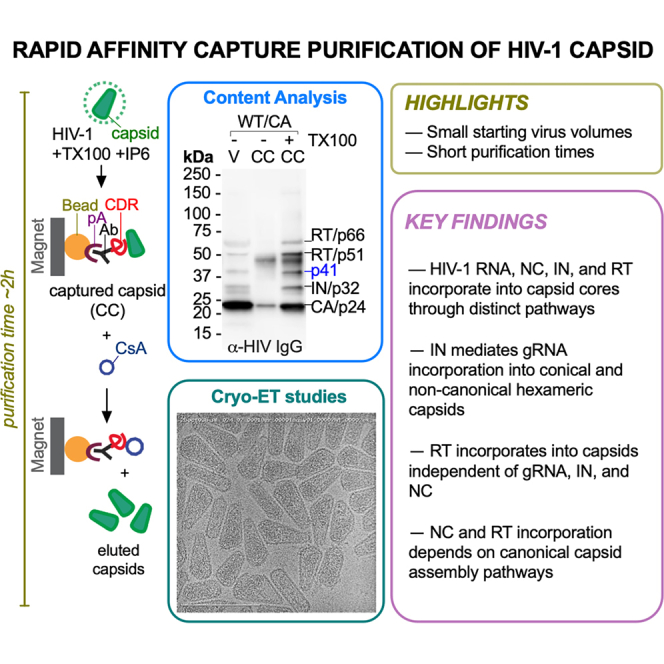

## Introduction

HIV-1 assembly at the plasma membrane is orchestrated by precursor viral Gag- and Gag-Pol polyproteins. The viral genomic RNA (gRNA) becomes encapsidated into assembling virions through specific interactions between the packaging signal within gRNA and the nucleocapsid (NC) domain of Gag.^1^^,^^2^^,^^3^^,^^4^ During egress of immature virus particles, the viral protease (PR) initiates the process of HIV-1 maturation. PR cleaves Gag and Gag-Pol polyproteins at defined sites to release their individual domains, consisting of matrix (MA), capsid (CA), spacer peptide (SP) 1, NC, SP2, and p6 for Gag, and PR, reverse transcriptase (RT), and integrase (IN) for Pol.

During maturation, it is generally thought that NC, IN, and RT condense with gRNA to form the viral ribonucleoprotein complex (vRNP).^1^^,^^3^ Simultaneously, proteolytically released CA assembles into ∼250 hexamers and 12 pentamers to form a closed conical capsid structure. The proper orchestration of the HIV-1 maturation process leads to the formation of a structurally mature virus with the vRNP incorporated into the capsid shell.^5^^,^^6^ The exact sequence of events driving vRNP formation, including the mechanisms of how the gRNA and the contents of the viral core are incorporated into the assembling capsid shell—referred here to as structural maturation of the viral core—remain unclear.

It is minimally known that the binding of HIV-1 IN to gRNA plays a key role in the structural maturation of the viral core.^7^ Deletion of IN from virus particles^8^ or mutations in IN that prevent its binding to gRNA^7^ (referred to as class II IN mutants), as well as production of HIV-1 in the presence of allosteric integrase inhibitors (ALLINIs) that aberrantly hyper-multimerize IN and disrupt gRNA binding, result in the formation of “eccentric” virions, characterized by vRNP-electron densities mislocalized outside of the protective capsid shell.(reviewed in ^9^^,^^10^) By contrast, encapsidation of vRNPs and infectivity of IN-deleted viruses were partially restored by *trans*-incorporation of wildtype (WT) IN, demonstrating an important role for IN in functional HIV-1 morphogenesis.^11^ Additionally, IN-gRNA forms an extended filament structure that interacts with the inner wall of the mature capsid lattice.^12^ Although this recent study elucidated the structural basis for IN-gRNA incorporation, it remains unclear whether other vRNP components, including RT and NC, are also recruited into capsid through IN-gRNA interactions during viral core assembly.

Given its critical role in virus replication, the HIV-1 capsid has become a key target for antiviral drug development. Small molecule inhibitors, including PF74^13^ and the clinically approved drug lenacapavir (LEN),^13^^,^^14^^,^^15^ block HIV-1 infection by binding the assembled capsid structure^13^^,^^15^^,^^16^^,^^17^ and perturbing its functions. LEN potently (EC_50_ ∼50 pM) inhibits the early steps of HIV-1 replication via hyper-stabilizing capsids, blocking virus nuclear import and integration.^13^^,^^18^^,^^19^^,^^20^^,^^21^^,^^22^^,^^23^^,^^24^ Moreover, when present during virus production, LEN can inhibit late maturation steps by promoting “off-pathway” capsid assembly,^25^ which appears to depend on the stoichiometry of LEN-to-CA in solution.^20^^,^^26^ However, the precise mechanisms by which LEN interferes with HIV-1 maturation remain unclear. The development of LEN, which has demonstrated efficacy in pre-exposure prophylaxis for preventing new HIV-1 infections,^27^^,^^28^ along with emerging capsid-binding drugs in clinical development,^29^^,^^30^ underscores the importance of studies focused on the structural maturation of HIV-1 cores.

Current methods for studying the content of mature HIV-1 cores rely on tedious procedures requiring ultracentrifugation of virus particles followed by biochemical analysis of sucrose gradient fractions.^31^^,^^32^^,^^33^^,^^34^^,^^35^ While informative, these methods require large starting volumes of viral supernatant and offer limited scalability. Nevertheless, they have been instrumental in characterizing capsid architecture and identifying key viral core components^31^ RT, IN, NC, and gRNA, as well as the accessory protein Vpr, which is incorporated into virions and cores through interactions with the p6 domain of Gag.^32^^,^^34^^,^^35^^,^^36^ However, interpreting data from fractionation studies can be challenging. Components of the vRNP—including NC, IN, RT, and gRNA—co-immunoprecipitate,^37^ and mislocalized vRNPs from eccentric virions often co-migrate with intact capsid fractions in sucrose gradients.^33^ These limitations underscore the need for a more robust and scalable approach to investigate how viral contents are incorporated into assembling capsids during HIV-1 morphogenesis.

In this study, we leveraged the high-avidity capsid-binding property of a recombinant cyclophilin A-DsRed (CDR) fusion protein^38^ to rapidly enrich native HIV-1 capsids from a comparatively small starting volume (∼2 mL) of virus supernatant. Notably, the purification process is completed within ∼2 h. Using single-virus imaging, cryo-electron microscopy (cryo-EM), and immunoblot analyses, we demonstrate that our rapid affinity capture approach enriches authentic HIV-1 capsids in sufficient quantities for biochemical and structural studies.

We then applied our streamlined approach to examine the role of IN-gRNA interactions (disrupted via ALLINI treatment or IN-deletion) and of the assembling capsid structure (disrupted via LEN treatment or cross-linking of CA hexamers) on vRNP content incorporation and viral core morphogenesis. Consistent with the eccentric morphology and mislocalization of vRNP-electron density outside of capsid structures following disruption of IN-gRNA interactions,^7^^,^^8^^,^^11^^,^^33^^,^^39^ affinity captured cores (CCs) from ALLINI-treated or IN-deleted virions showed a strong depletion of gRNA, IN, and NC components—validating the approach. However, despite the strong depletion of gRNA, we found that RT remained core-associated under these conditions. By contrast, disruption of canonical capsid assembly by enforced cross-linking of CA hexamers^40^ or by LEN treatment of virus producing cells,^20^^,^^25^ reshaped the overall capsid architecture and selectively impaired the incorporation of RT and NC proteins, without affecting IN-gRNA recruitment into the viral core. Based on these observations, we suggest distinct pathways for recruitment of vRNP components RT, NC, and IN-gRNA into capsids, and underscore cooperative roles for IN and the assembling capsid architecture during the structural maturation of HIV-1 cores.

## Results

### CDR selectively binds to mature HIV-1 capsids *in vitro*

We previously described the construction and utility of the CDR fusion construct for HIV-1 imaging studies.^38^ CDR is packaged into assembling HIV-1 virions via interactions between its cyclophilin A (CypA) domain and G89/P90 residues within the CypA-binding loop of the Gag CA domain. Here, we examined the binding properties of CDR to cores isolated from fully assembled virions. Time-resolved imaging showed that CDR from a cellular extract rapidly bound (∼2.5 min) to mature assembled WT capsids in the low nanomolar (<50 nM) range (Figures S1A and S1B), and failed to bind control G89V mutant capsids that do not bind CypA (Figures S1A and S1B). Importantly, CDR showed selective binding to mature WT capsids, illustrated by a lack of binding to immature structures produced in the presence of the PR inhibitor saquinavir (Figures S1A and S1B). Although CypA in CDR engages the CypA-binding loop on CA at 1:2 stoichiometry,^38^ DsRed-tetramerization facilitated CypA multimerization increases the affinity and overall avidity of the CypA-capsid interaction.^41^ Plausibly, the reduced interaction with immature particles reflects limited accessibility of the large (∼200 kDa) CDR tetramer to tightly packed CA domains within Gag precursors.^42^

### Rapid affinity capture purification of native HIV-1 cores

**Figure 1:**
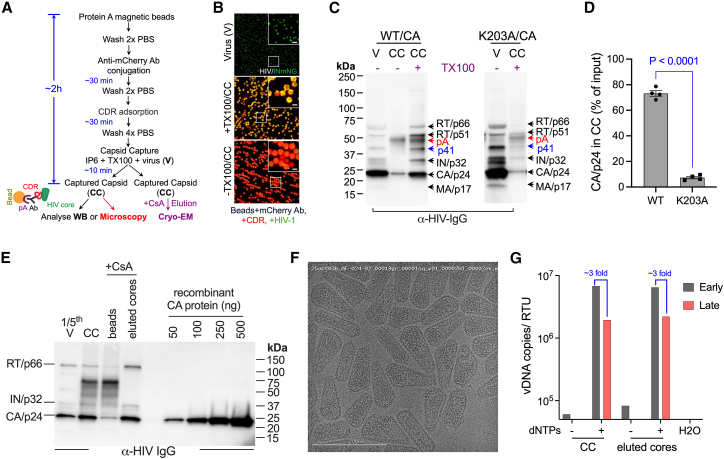
Affinity capture purification of native HIV-1 cores (A) Schematic of the affinity capture approach highlighting the short ∼2 h duration of the protocol and a cartoon of the captured capsid analyzed by microscopy and immunoblot (WB) analysis. (B) Confocal images showing INmNG-labeled virus and cores (green), captured by CDR-coated beads in the presence (+) or absence (−) of TX100 treatment. Scale bars in inset, 1 μm. (C) Content analysis of affinity captured cores (CC) by anti-HIV serum immunoblots of WT *(*left side*)* and unstable K203A *(*right side) virions (V) revealed selective capture of assembled cores under TX100 permeabilization. Known viral proteins (black arrows), including ∼ p41 (blue arrows), and non-specific protein-A (pA) (red arrow), are overlayed on the blots for clarity. (D) Densitometry analysis of CA/p24 present in CC as a percentage of the same in virions from *n* = 4 experiments. See Figure S2A for additional examples of WT and K203A V and CC samples loaded in parallel. Data are represented as mean ± SEM. Student’s *t* test was used to determine statistical significance. (E) Efficiency of capsid capture and elution by CsA. Immunoblots show 1/5^th^ of input virions (V), captured cores (CC), the beads after CsA-elution, and eluted cores (+CsA). For capture efficiency comparison, the indicated amounts of recombinant CA protein were loaded on the blot. (F) Cryo-EM images collected at 37,000× magnification show a cluster of isolated HIV-1 capsids. Scale bars, 200 nm. See also related (Figures S3A–S3D). (G) ERT data indicating the extent of early (minus strand strong stop, gray) and late (full-length minus strand, red) vDNA products synthesized by affinity-captured cores on beads (CC) and CsA-eluted cores in the presence (+) or absence (−) of dNTPs. Data show absolute copy number of RT products from one experiment, and fold-differences between early and late RT products from *n* = 4 experiments, see also related Figure S3E.

Building on these observations, we asked if we could repurpose the CDR-probe to develop an affinity capture strategy to selectively enrich mature HIV-1 cores for biochemical and structural studies (Figure 1A). Toward this goal, a small volume (2 mL) of virus supernatant was clarified of cellular debris by polyethylene glycol (PEG) precipitation, and the resulting virus pellet was resuspended in phosphate-buffered saline (PBS) at one-tenth of the starting volume. We used protein A-coated magnetic beads to bind antibodies against the DsRed domain to capture CDR and retain the CypA-domain available to bind capsids from Triton ×100 (TX100)-permeabilized virions. Inositol hexakisphosphate (IP6, 100 μM) was included in the capture buffer to stabilize the exposed capsid lattice^41^^,^^43^^,^^44^ and enable the enrichment of structurally intact cores.

CC were either imaged by confocal microscopy (via a fluorescently tagged, core-associated Vpr-IN-mNeonGreen (INmNG) fusion protein)^45^ or by SDS-PAGE and immunoblotting with anti-HIV serum when unlabeled cores were analyzed (Figure 1A). Initial tests confirmed that anti-mCherry monoclonal or polyclonal antibodies effectively captured CDR and enriched HIV-1 capsids (Figure S1C). Titration experiments showed that as little as 1 μg of antibody and/or 100 ng of CDR, combined with 15 μL of protein A-coated magnetic beads, isolated sufficient HIV-1 capsids from 5 μL of 10×-concentrated virions for immunoblot analysis (Figures S1D and S1E). These results highlight the feasibility of HIV-1 core biochemical studies starting from a relatively small volume of virus supernatant and over a comparatively short time frame (∼2 h, Figure 1A), compared to ∼30 h required for traditional ultracentrifugation and gradient fractionation approaches.^46^

Confocal microscopy revealed punctate INmNG-labeled cores bound to the surface of beads after TX100 treatment (Figure 1B); fluorescent puncta were not observed without detergent, confirming that membrane permeabilization is essential to capture HIV-1 capsids using our CDR probe. Immunoblot analysis corroborated these findings and revealed robust capture of unlabeled native HIV-1 cores only under permeabilizing conditions (Figure 1C, left). Viral proteins detected in both input (V) and CCs included known core components RT (p66 and p51 subunits), IN, and CA, as well as a ∼41 kDa intermediate Gag cleavage product (blue arrows in Figure 1C, left; Figures S1C–S1E) that was reported to migrate with core fractions in earlier studies.^34^^,^^35^ While presumable membrane-associated MA/p17 was detected in the input virus, it was absent in CC fractions, indicating successful exclusion of membrane material. The ∼50 kDa cross-reactive species found only in CC lanes (red arrows in Figure 1C; Figures S1C–S1E) was identified as non-specific binding of anti-HIV serum to protein A (pA).

### Selective enrichment of assembled capsids from membrane-permeabilized virions and infected cells

Next, to rule out the possibility that CDR-mediated capture enriched for disassembled, monomeric CA/p24 rather than intact capsids, we performed experiments using the unstable K203A CA mutant, which rapidly disassembles upon virus membrane permeabilization.^31^^,^^38^^,^^47^ In contrast to the successful enrichment of WT capsids, CDR failed to recover significant amounts of CA/p24 or other viral proteins from K203A cores (Figure 1C, right; Figure S2A). Densitometry analysis (Figure 1D) revealed efficient recovery (∼70%) of WT CA/p24, with <9% CA/p24 recovered from K203A viruses. These results confirmed that CDR efficiently captures assembled, mature capsids rather than disintegrated components from membrane permeabilized virions.

To test the applicability of our approach in a cellular context, we adapted a modified “fate-of-capsid” assay^48^^,^^49^^,^^50^ to assess whether CDR could capture capsids following HIV-1 cellular entry. HEK293T cells were infected with virus envelop glycoprotein G (VSV-G)-pseudotyped WT or CA mutants K203A or E45A, which represent intermediate, unstable, and hyper-stable capsid phenotypes, respectively.^38^^,^^47^ At 4 h post-infection, cells were lysed with a hypotonic buffer, and CDR capture was used to enrich intracellular capsids. Immunoblot analysis of cell-derived cores (Figure S2B) revealed distinct CA/p24 signals corresponding to HIV-1 capsid stability: strong enrichment for hyper-stable E45A, moderate enrichment for WT, and lack of enrichment for the unstable K203A mutant. These results further validate the specificity of affinity capture to selectively enrich stable, assembled, mature capsids, both from permeabilized virions and from infected cells.

### Enriched HIV-1 cores have authentic conical morphology

To determine the morphology of affinity captured capsids, we used cyclosporin A (CsA), a small molecule that displaces CypA from capsid,^38^ to release the CCs from CDR-coated beads (Figure 1A). Immunoblot analysis showed that ∼50–100 ng of CA/p24 protein was captured from ∼250 ng of input virus material, and that CsA efficiently released most of the bound cores (Figure 1E). Negative-stain EM of CsA-released cores (Figure S3A) confirmed the selective enrichment of mature capsid structures as aggregated groups. To obtain a more detailed view of the eluted cores, we used high-magnification and high-contrast cryo-EM imaging to visualize the individual capsid structures within aggregated groups (Figures 1F and S3B). Structural classification and analysis of cryo-EM images revealed that ∼86% of eluted cores were conical in shape (Figure S3C). Aside from a few presumably broken cones, the majority (∼95%, *n* = 123) exhibited well-defined morphologies^6^ with distinct measurements of ∼65 and ∼30 nm for the broad and narrow ends, respectively, and overall average length of ∼120 nm (Figure S3D). The remaining cores (∼14%) corresponded to apparent tubular structures (Figure S3C), which is consistent with fractions of conical and tubular capsid morphologies identified in core preparations using conventional approaches.^51^

### Enriched HIV-1 cores are competent for ERT

To determine the functional integrity of isolated cores, we evaluated their ability to carry out endogenous reverse transcription (ERT)^23^^,^^52^ and synthesize full-length viral DNA (vDNA) *in vitro*. Quantitative PCR (qPCR) analysis revealed that both CCs immobilized on beads and CsA-eluted cores synthesized full-length vDNA products at levels ∼3-fold lower than early reverse transcription products (Figure 1G), which was reproducible in four independent experiments (Figure S3E) and comparable with prior studies.^23^^,^^52^^,^^53^ As expected, vDNA products were not observed in the absence of added dNTPs (Figure 1G). These results demonstrate that HIV-1 cores enriched by affinity capture are functionally competent for full-length vDNA synthesis, which is thought to occur within the confines of a morphologically intact capsid.^54^

### Contents of enriched HIV-1 cores

**Figure 2:**
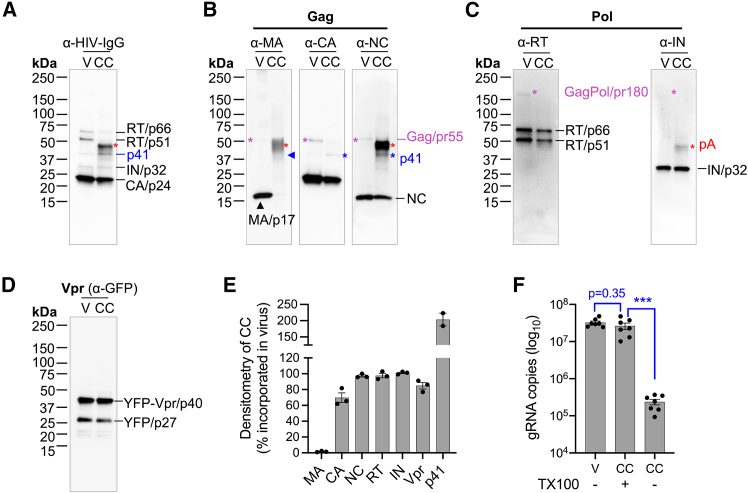
Contents of HIV-1 cores (A–D) Immunoblots of input virus (V) and contents of affinity captured cores (CC) probed with (A) anti-HIV serum, (B) antibodies against Gag products: MA, CA and NC, (C) antibodies against Pol products: RT and IN, and (D) antibodies against GFP to detect YFP-Vpr. Mature viral proteins (black), unprocessed Gag-/Gag-Pol (pink stars), p41 Gag-processing intermediate (blue star), and non-specific protein-A carried over from beads (red star) are overlayed on the blots for clarity. (E) Densitometry analysis showing the normalized fraction of viral proteins detected in affinity captured cores (CC). Data are mean ± SEM of *n* = 3 independent capture experiments. See also related Figure S4 and Table S1. (F) RT-qPCR analysis of gRNA-copy numbers in input virus (V) and captured cores (CC) highlight specific enrichment of gRNA in CC in the presence of TX100 permeabilization but not its absence. Data are mean ± SEM of *n* = 7 independent capture experiments. Student’s *t* test was used to determine significance (F); ∗∗∗*p* < 0.001.

We next further analyzed the contents of enriched capsids using protein-specific antibodies. As before (Figures 1C and 1E), anti-HIV serum identified multiple species corresponding to RT (p66/51), IN (p32), CA/p24, and the ∼41 kDa Gag processing intermediate^35^ in both input virions and captured capsids (Figure 2A). Immunoblotting for Gag components—MA, CA, and NC—revealed several key features (Figure 2B): (1) MA/p17 was detected only in virions and was excluded from CC; (2) full-length Pr55 Gag precursor was enriched in virions, with little-to-none detected in CC; (3) NC was present in CC; and (4) the ∼41 kDa Gag processing intermediate, which was enriched in CC, was detected with anti-CA and anti-NC antibodies, but not with anti-MA antibodies.

For a more specific identification of the ∼41 kDa product, we analyzed an in-gel band (Figure S4A) by trypsinization and mass spectrometry. Peptide enrichment analysis showed uniquely abundant peptides corresponding to CA, SP1, and NC-SP2 segments of the Gag precursor protein (Figure S4B; Table S1), and, consistent with the immunoblotting results, peptides corresponding to MA were comparatively underrepresented. Although others have indicated the processing intermediate is composed of MA-CA,^34^ based on the detection of NC in immunoblots and significantly higher enrichment (∼21-fold) of NC-SP2 peptides compared to MA by mass spectrometry (Figures 2B and S4B; Table S1) we surmise the p41 product may also contain p6 (which was not probed here) to compose CA-SP1-NC-SP2-p6, with a theoretical molecular mass of 41 kDa.

Immunoblotting for Pol components confirmed the retention of proteolytically mature RT p66/p51 and IN in CC (Figure 2C). By contrast, the uncleaved Gag-Pol precursor (∼160 kDa) was preferentially excluded from CC and detected only in virions. To assess Vpr incorporation, we generated yellow fluorescence protein (YFP)-Vpr-labeled virions^55^^,^^56^ and detected the fusion protein in CC using anti-GFP antibodies, which additionally confirmed Vpr as a bona fide core component (Figure 2D), in agreement with prior reports.^31^^,^^34^^,^^36^

Comparative densitometry analysis (Figure 2E) revealed that ∼70% of input CA/p24 and >80% of NC, RT, IN, and Vpr were associated with CC, while the p41 Gag intermediate product was ∼2-fold enriched. The detection of ∼70% CA/p24 in cores is consistent with the ∼1,600 of total ∼3,000 CA proteins that assemble into capsids^5^^,^^6^^,^^42^^,^^57^; comparatively higher retention (>80%) of NC, RT, IN, Vpr, and p41 (Figure 2E) is consistent with their selective incorporation into HIV-1 cores. As expected, quantitative reverse-transcription PCR (RT-qPCR) assessment of gRNA content (Figure 2F) revealed practically full recovery in CC as compared to input virions (*p* = 0.35). Importantly, these gRNA levels were significantly (∼100-fold) enriched in CC compared to control samples with non-permeabilized virions, ruling out nonspecific binding of gRNA to CDR-coated beads and confirming selective capture of genome-containing cores. These results (Figures 1 and 2) validate and confirm that our CDR-affinity capture procedure isolates *bona fide* HIV-1 cores.

### AllostericIN inhibitors selectively deplete IN, NC and gRNA from HIV-1 capsids

We next asked whether our affinity-capture approach can detect the previously established disruption of IN-, NC-, and gRNA incorporation into HIV-1 cores from virions produced in the presence of ALLINIs.^9^ Toward this goal, we generated HIV-1 particles in cells cultured in the presence of KF116, a potent pyridine-based ALLINI,^58^ and used CDR to capture the resulting viral cores. Controls included viruses treated with DMSO (vehicle) or the IN strand transfer inhibitor raltegravir (RAL),^59^ which affects integration but is not expected to affect maturation.^39^

We noted that neither DMSO nor RAL treatment impacted virus production (Figure S5A) and that PEG precipitation of virions effectively removed RAL (which would otherwise block subsequent integration) from solution, resulting in equal infectivity of RAL- and DMSO-treated virions (Figure S5B). By comparison, KF116 treatment, which also had no effect on virus production, led to a severe infectivity defect (Figures S5A and S5B), which is consistent with the known effects of ALLINIs on HIV-1 maturation.^11^^,^^39^^,^^58^^,^^60^

**Figure 3:**
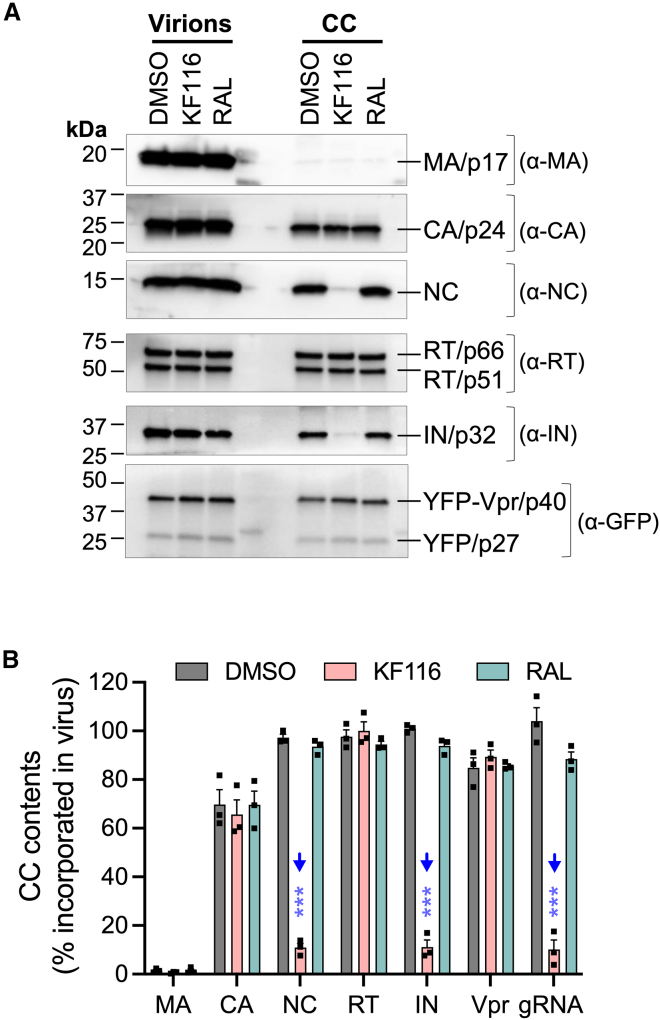
Allosteric IN inhibitors selectively deplete IN, NC, and gRNA, without affecting RT and Vpr incorporation into HIV-1 cores (A and B) Immunoblots (A) and content analysis (B) of virions and captured cores produced in the presence of DMSO, KF116 (1 μM), or RAL (10 μM). Blue arrows in B point to vRNP components (NC, IN, and gRNA) specifically depleted in CC. Densitometry of viral proteins (mean ± SEM for *n* = 3 independent experiments) and qPCR-gRNA (mean ± SEM for *n* = 3) analysis, shown in (B), were normalized as fractions of input virus proteins and gRNA. Statistical significance in (B) was determined by pairwise Student’s *t* test (vs. DMSO); ∗∗∗*p* < 0.001 (other comparisons to DMSO, *p* > 0.05, were considered not significant). See also related Figure S5.

Immunoblot analysis of virions revealed comparable levels of MA, CA, NC, RT, IN, and Vpr across conditions (Figure 3A, left lanes). However, CC analysis (Figure 3A, right lanes) confirmed selective depletion of IN and NC from KF116-treated, but not from control DMSO- or RAL-treated cores. Interestingly, RT and Vpr were retained in CCs at similar levels across all conditions (Figure 3A, right lanes), indicating that their incorporation into capsids was unaffected by KF116 treatment. Densitometry (Figure 3B) confirmed exclusion of MA- and retention of ∼70% of CA/p24 signals in CC, compared to input levels. Furthermore, while >90% of virion-incorporated IN and NC was retained following DMSO- and RAL-treatments, <12% of each protein remained in capsids following KF116 treatment, eliciting an ∼7.5-fold inhibition of IN- and NC incorporation. By contrast, RT (∼97%) and Vpr (∼80%) were consistently recovered in CC across all treatments.

In comparison to DMSO and RAL controls, KF116 significantly depleted (>5-fold reduction) gRNA incorporation into capsids (Figure 3B). Negative stain EM of cores isolated from DMSO, KF116, and RAL-treated samples revealed canonical capsid structures (Figure S5C). Cryo-EM further revealed an apparent lack of vRNP electron density inside canonically assembled capsids isolated from virions produced in the presence of KF116, when compared to DMSO (Figures S5D and S5E). These results confirm the known effects of ALLINIs in disrupting the incorporation of IN, NC, and gRNA into the viral core, leading to formation of eccentric virus particles.^7^^,^^60^

### RT incorporates into capsids independent of IN, NC, and gRNA

**Figure 4:**
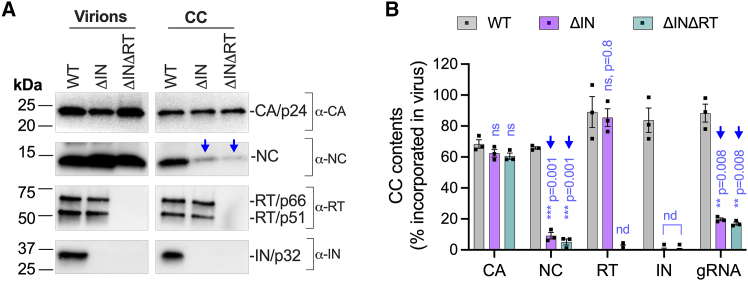
RT is incorporated into capsids independent of IN, NC, and gRNA (A and B) Immunoblots (A) and capsid content analysis (B) of WT, IN-deleted (ΔIN), or RT and IN-deleted (ΔINΔRT) virions and captured capsids. Blue arrows in (A and B) highlight the proteins and gRNA present in virions but depleted in CC. Densitometry of viral proteins (mean ± SEM for *n* = 3 independent experiments) and qPCR-gRNA (mean ± SEM for *n* = 3) analysis, shown in (B), were normalized as fractions of input virus proteins and gRNA. Statistical significance in (B) was determined by pairwise Student’s *t* test (vs. WT), *p* values ∗∗*p* < 0.01, and ∗∗∗*p* < 0.001 were considered significant; *p* > 0.05 was considered not significant (ns). See also related Figure S6 gRNA copies in virions vs. CC.

The accessory protein Vpr is incorporated into mature virions and capsids *via* direct interaction with Gag p6, independent of IN or gRNA.^35^^,^^36^ In contrast, RT is an essential component of vRNPs that is required for converting gRNA into vDNA during infection. RT accordingly binds gRNA during reverse transcription and is also known to interact with IN.^61^^,^^62^^,^^63^ It therefore seemed possible that RT interactions with gRNA and/or IN might mediate its incorporation into HIV-1 CA cores. The unexpected observation that RT is retained in ALLINI-treated capsids independently of other vRNP components prompted us to further investigate the role of IN in RT incorporation. We accordingly analyzed the contents of CC from HIV-1 particles lacking IN (ΔIN)^8^ or both RT and IN (ΔINΔRT).^7^ Immunoblotting and densitometry analysis confirmed unperturbed proteolytic processing of polyproteins (Figure 4A left, and 4B) and, as expected, RT and IN absence from respective deletion mutants; gRNA packaging into virions was also unaffected by either deletion (Figure S6). However, captured capsids from IN- and RT-IN-deleted viruses showed, as expected, a marked depletion of IN, NC, and gRNA (Figures 4A right, 4B; Figure S6). Importantly, when compared to the WT, an equal proportion of RT heterodimers (p66/p51) were present in cores from IN-deleted virions (Figures 4A right, and 4B), suggesting that RT incorporates into capsids independently of IN, NC, and gRNA.

### Lenacapavir selectively depletes RT and NC from IN-gRNA containing HIV-1 cores

We next examined how LEN affected the structural maturation of HIV-1 cores.^15^^,^^20^^,^^26^ Toward this goal, we produced VSV-G pseudotyped HIV-1 particles in the presence of varied concentrations of LEN (0–100 nM), followed by PEG-precipitation of virions to remove the drug from solution.^26^ We then examined the concentration-dependence of late-stage effects of LEN on HIV-1 particle release, maturation, infectivity, and capsid content.

**Figure 5:**
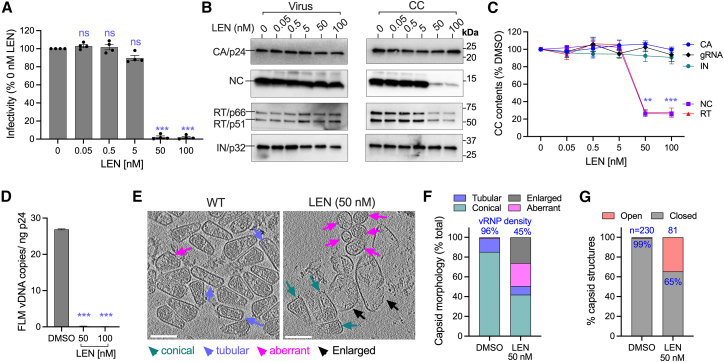
LEN impairs HIV-1 maturation by reshaping the structure and composition of viral cores (A) Quantification of LEN effects on infectivity of VSV-G pseudotyped HIV-1 produced in the presence of the indicated drug concentrations. LEN was washed out by PEG-precipitation of virus, and equal RT-inputs of the different viral preparations were used to determine infectivity in TZM-bl target cells. Data are mean ± SEM for *n* = 4 experiments. See related Figure S7A for LEN’s effect on virus release. (B and C) Immunoblots (B) and capsid content analysis (C) of virus and captured cores (CC) produced in the presence of indicated LEN concentrations. Data in (C) densitometry of viral proteins (mean ± SEM for *n* = 3 independent experiments) and qPCR-gRNA (mean ± SEM for *n* = 3) analysis, were normalized as fractions of DMSO (0 nM LEN) control. See related Figure S7B for densitometry of proteins in virions related to (C). (D) ERT assay of full-length vDNA by purified capsids produced in the presence of DMSO and indicated LEN concentrations. FLM vDNA copies (mean ± SEM for *n* = 3 experiments) were normalized to input CA/p24 concentrations. (E–G) Representative projection of 10 slices through cryo-ET volumes (E), quantification of morphology (F), and intactness (G) of purified capsids produced in the presence of 0 or 50 nM LEN. Colored arrows in (E) points to different conical (cyan), tubular (purple), aberrant (magenta) and enlarged (black) structures detected and quantified in (F) from the cryo-ET datasets. The fraction of cores retaining internal vRNP density is shown in (F), the number of structures (“*n*”) analyzed are in (F and G), including the fraction of closed cores in the cryo-ET datasets in (G). See related (Figure S8 and Videos S1 and S2) for more examples. Scale bars: 100 nm in (E). Statistical significance in (A), (C), and (D) was determined by pairwise Student’s *t* test (vs. DMSO), *p* values ∗∗*p* < 0.01, and ∗∗∗*p* < 0.001 were considered significant. Note, *p* values in (C) for NC and RT were 0.002 (∗∗) and <0.001 (∗∗∗) for 50 and 100 nM LEN, respectively. All other comparisons yielded *p* > 0.05, which was considered not significant.

Consistent with prior reports,^15^^,^^20^ we noted a marked reduction in virus particle release and RT activity in viral supernatants associated with increasing concentrations of LEN treatment (Figure S7A). After normalizing virus input, single-round infectivity assays showed that LEN concentrations ≥50 nM potently inhibited the production of infectious virions (Figure 5A), revealing the concentration-dependent effects of LEN to inhibit the late stages of HIV-1 replication. However, immunoblot analysis of virions showed that LEN had no noticeable effect on proteolytic processing, as the mature forms of CA, NC, RT, and IN were equally detected across all conditions (Figure 5B, left).

In stark contrast to the lack of effect on virion protein content, at inhibitory concentrations RT and NC levels were significantly depleted from CC (Figures 5A and 5B, right), which was quantified by densitometry (∼4-fold, Figure 5C). Interestingly, despite the depletion of NC from cores, the levels of IN and, importantly, of gRNA incorporation into capsids remained constant (Figure 5C). Furthermore, corroborating the selective partitioning of IN-gRNA inside, and of RT and NC outside, of the assembled capsids, HIV-1 cores produced in the presence of ≥50 nM LEN showed a marked reduction in full-length vDNA synthesis (Figure 5D). These findings highlight that LEN inhibits the encapsulation of vRNP components, specifically RT and NC, through a mechanism that is distinct from the effects observed by ALLINI treatment and IN deletion (Figures 3 and 4).

### Lenacapavir reshapes capsids by promoting the formation of flatter and enlarged structures

To explore whether the changes in core content and infectivity (Figures 5A–5D) correlated with the reported effects of LEN on promoting aberrant capsid assembly and morphology,^20^^,^^25^^,^^26^ we eluted CC with CsA and analyzed their structures by negative-stain EM. At low LEN concentrations (0–0.5 nM), the eluted cores, as expected, retained the WT conical morphology (Figure S8A). In contrast, at ≥50 nM, LEN induced the formation of readily visible abnormal, flatter, and enlarged hexagonal shaped capsid structures (Figure S8A). Morphological classification confirmed a concentration-dependent shift from conical to enlarged aberrant forms starting at 5 nM, with predominantly aberrant morphologies at ≥50 nM LEN (Figure S8B).

To gain a deeper insight into the morphological intactness of enriched cores we used cryo-electron tomography (cryo-ET) to analyze the 3-dimensional (3D) architecture of the enlarged capsids produced in the presence of 50 nM LEN. In control DMSO-treated samples, cryo-ET and sub-volume analysis of tomograms showed enrichment of canonical capsid structures (Figure 5E and Video S1). By contrast, cores produced in the presence of 50 nM LEN showed equal presence of both canonical and aberrant enlarged structures (Figure 5E and Video S2). Morphological classification of capsid shapes (Figure 5F) showed a 2-fold reduction in the fraction of conical (∼85% DMSO; and ∼42% LEN) and tubular capsids (∼16%, DMSO; and 8%, LEN) following LEN treatment. By contrast, the aberrant (1% DMSO; and ∼24% LEN) and enlarged (0% DMSO; and ∼26% LEN) capsid populations were specifically enriched by LEN treatment (Figure 5F).

Video S1. Cryo-ET reconstruction showing canonical capsid structures of DMSO control cores, related to Figure 5 scale bars, 100 nm.

Video S2. Cryo-ET reconstruction showing aberrant capsid morphology of cores produced in the presence of 50 nM LEN, related to Figure 5 scale bars, 100 nm.

Importantly, nearly all (∼96%) DMSO-treated capsids retained discernable internal electron densities, presumably corresponding to vRNPs, which was reduced 2-fold (∼45%) by LEN treatment (Figure 5F). 3D-analysis of tomographic reconstructions showed that over ∼98% of conical and tubular capsids remained closed in both DMSO- and LEN-treated samples (Figure 5G; Videos S1 and S2). By contrast, nearly all of the enlarged structures, and half of the aberrant structures (totaling ∼35%), showed some sort of abnormality, with open/broken ends and curved continuous lattices (Figure 5E and Video S2), which is consistent with prior observations.^20^^,^^25^ Given that ∼65% of LEN-treated capsids were deemed closed in our analysis (Figure 5G), we surmised that the excessive ∼80% loss of RT and NC from these cores (Figure 5C) was likely due to incorporation defects, rather than leakage of these proteins from broken structures.

To further determine if NC and RT were excluded during capsid assembly rather than lost during the purification process, we treated cores with LEN after virus production and analyzed the contents of captured capsids. Notably, CC-content analysis showed complete retention of RT and NC proteins, as well as IN-gRNA, in cores treated with up to 100 nM LEN (Figures S9A and S9B), at levels comparable to those observed in no-LEN treatment controls. Although LEN treatment has been reported to disrupt the morphological intactness of cores,^13^^,^^22^^,^^24^ we note that LEN (up to 100 nM) did not affect the overall intactness of cores purified through the CC-approach (Figures S9C and S9D), perhaps due to the presence of IP6 in the reaction. Conversely, these findings indicate that the reduction in RT and NC observed during HIV-1 capsid assembly in the presence of LEN (Figure 5) reflects a failure to incorporate these components into nascent cores rather than their loss from capsid breakage during the core purification procedure.

Together (Figures 5 and S7–S9), our results highlight: (1) concentration-dependent effects of LEN on HIV-1 assembly, release, and maturation, (2) LEN-mediated formation of abnormal, flatter, enlarged and open-ended hexagonal capsids at the expense of canonical capsid assembly, (3) retention of IN and gRNA by LEN-induced abnormal capsids, and (4) the ability of LEN and of the assembling capsid architecture to regulate the incorporation and/or retention of RT and NC in viral cores.

### Enforced cross-linking of CA hexamers prevents RT and NC incorporation into cores

Finally, we asked if the impaired recruitment of RT and NC into LEN-treated viral cores could be related to the predominantly hexameric capsid assembly promoted by LEN.^25^^,^^26^^,^^41^ Toward this goal, we analyzed the contents of cysteine-crosslinked A14C/E45C CA mutant cores that are known to assemble comparatively flat hexameric capsids *in vitro.*^40^

**Figure 6:**
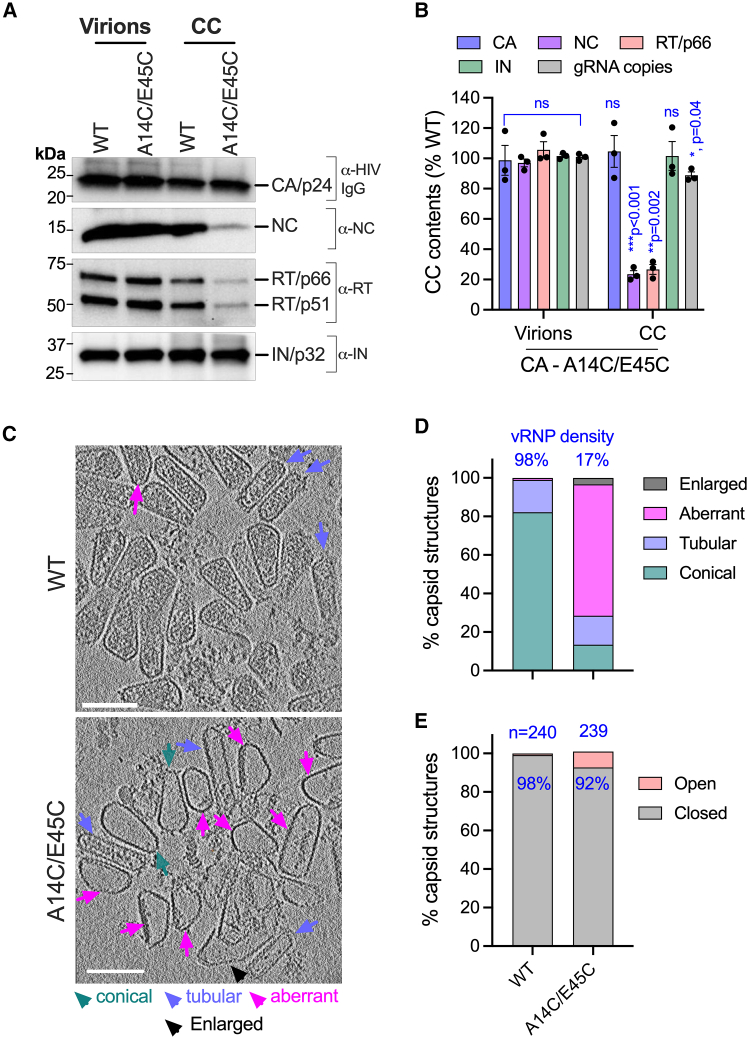
Cross-linked A14C/E45C capsids fail to incorporate RT and NC (A and B) Immunoblots (A) and capsid content analysis (B) of WT and A14C/E45C CA mutant virions and captured cores (CC). Densitometry of viral proteins and qPCR-gRNA in (B) (mean ± SEM for *n* = 3 independent experiments) shown as normalized fractions of WT virus and CC. Statistical significance was determined by pairwise Student’s *t* test of A14C/E45C vs. WT. ∗∗∗*p* < 0.001; ∗∗*p* < 0.01; ns, *p* > 0.05. (C–E) Representative projection of 10 slices through cryo-ET volumes (C), quantification of morphology (D), and intactness (E) of purified WT and cross-linked A14C/E45C cores. Colored arrows in (C) point to different conical (cyan), tubular (purple), and aberrant (magenta) structures, including enlarged (black) entities detected and quantified in (D). The fraction of structures containing electron densities inside capsid is shown in (D), and the number (“*n*”) of cores analyzed, including the fraction of closed cores in the cryo-ET datasets, in (E). See related (Videos S3 and S4) for more examples. Scale bars: 100 nm in (C).

Immunoblot analysis of virions showed equal packaging and proteolytic processing of CA, NC, RT, and IN in A14C/E45C and WT virions (Figure 6A, virions). Importantly, by contrast to the equal presence of CA and IN in both WT and A14C/E45C mutant CC, the captured cross-linked hexameric cores were significantly depleted for RT and NC (Figure 6A, *CC*). Compared to the WT, densitometry and gRNA-qPCR analysis (Figure 6B) revealed that whereas the incorporation of IN- and gRNA into capsids was unaffected by the A14C/E45C cross-linking mutations, RT and NC were significantly (∼3-fold) depleted. Furthermore, ERT assays (Figure S10) revealed the inability of A14C/E45C cores to synthesize full-length vDNA *in vitro*, supporting the partitioning of gRNA inside and RT outside of these capsids.

By contrast to the predominant conical capsid morphology—with clearly discernable internal vRNP electron densities—in CsA-eluted WT cores (Figure 6C, top and Video S3), cryo-ET imaging revealed cross-linked A14C/E45C capsids as pleomorphic with varied conical, tubular, aberrant, and enlarged shapes, with inner volumes transparent and devoid of electron density (Figure 6C, bottom and Video S4). Morphological classification (Figure 6D) showed similar fractions of tubular forms (∼16% WT; and 15% A14C/E45C) and a strong depletion of conical forms among A14C/E45C mutant cores (13%) compared to the WT (82%), including associated enrichments of aberrant (∼68%) and enlarged (∼3%) A14C/E45C capsids (Figure 6D). Notably, only ∼17% of the A14C/E45C cores showed some form of electron density internal to the capsids, which was significantly lower than the ∼98% detected in WT cores (Figure 6D). Finally, analysis of capsid closure showed that the overwhelming majority (∼92%) of the A14C/E45C crosslinked capsids were closed (Figure 6E, and Video S4), suggesting that the lack of RT and NC from these cores was likely due to incorporation defects.

Video S3. Cryo-ET reconstructions of captured wild-type capsids, related to Figure 6 scale bars, 50 nm.

Video S4. Cryo-ET reconstructions of captured A14C/E45C mutant capsids, related to Figure 6 scale bars, 50 nm.

## Discussion

Understanding the molecular mechanisms governing the incorporation of vRNPs into the mature HIV-1 capsid is critical for elucidating the processes that lead to the formation of an infectious viral particle. Here we introduce a rapid affinity capture method to streamline HIV-1 capsid purification steps for mechanistic and structural studies. Key advantages of the approach are: (1) specificity of CDR for mature, assembled capsids, (2) requirements for a small starting volume of viral supernatant (<2 mL) alongside a greatly diminished experimental time frame (∼2 h), and (3) yields consistent with high resolution structural analyses by cryo-EM and cryo-ET. Our method bypasses time-consuming gradient ultracentrifugation steps and alleviates the need for large-scale virus production used in conventional HIV-1 core purification approaches.^7^^,^^31^^,^^32^^,^^33^^,^^49^^,^^64^ Owing to its simplicity, robustness, and scalability, our rapid affinity capture approach may prove useful for future capsid-related studies, including core content analysis, ERT assays,^23^^,^^52^ and structural determinations.^12^

Affinity-capture identified the known canonical components of the HIV-1 core, including CA, NC, RT, IN, Vpr, and gRNA.^31^^,^^34^^,^^35^ Notably, we found the ∼41 kDa Gag intermediate cleavage product to be selectively enriched in cores. Earlier studies of HIV-1 cores, which also identified the Gag p41 kDa cleavage product, tentatively assigned this as MA-CA.^34^^,^^35^ However, our immunoblot and mass spectrometry data (Figures 2 and S4) showed that the ∼41 kDa product contains CA, NC and SP2 domains and very little, if any, MA. Whereas the p6 domain was not detected here, we propose that p6 is likely part of this precursor to make up the estimated 41 kDa mass. Together, these findings suggest that non-canonical Gag cleavage intermediates can also associate with the core during maturation. Future studies will be needed to address the extent and role of these intermediate cleavage products in the structural maturation of HIV-1 cores.

The ability to separate functionally distinct capsid populations and directly assess their structure and composition allowed us to investigate the effects of antiviral compounds, IN-deletion, and capsid cross-linking on HIV-1 maturation and functional core formation. Notably, ALLINIs and LEN impaired the structural maturation of HIV-1 cores (Figures 3A and 5B) and produced non-infectious virus particles.^7^^,^^15^^,^^26^^,^^33^^,^^39^ While it was known that ALLINIs block the incorporation of IN-gRNA and NC into cores,^7^^,^^9^^,^^11^^,^^33^^,^^39^^,^^60^ we found that the capsid inhibitor LEN can reshape the capsid structure and impair recruitment of RT and NC into aberrant capsids. These findings reveal that ALLINIs and LEN selectively impair distinct steps of HIV-1 core morphogenesis.

The results presented here further reinforce the essential role of IN in gRNA incorporation into the viral core and morphogenesis of infectious HIV-1 particles.^9^ Our observations that packaging of gRNA into viral particles was unaffected by deletion of IN and RT (Figure S6) suggest that as a part of the precursor Gag, NC in its immature form is sufficient for gRNA packaging during virus assembly. However, consistent with previous studies, deletion^8^ or aberrant multimerization of IN by ALLINIs^7^^,^^8^^,^^11^^,^^33^^,^^39^^,^^58^^,^^60^ led to the exclusion of NC and gRNA from captured capsids (Figure 3). These results recapitulate the eccentric morphology observed in electron microscopy studies and corroborate a model, wherein IN and its proper oligomerization is required for chaperoning the gRNA, together with NC, into the assembling capsid.^9^^,^^10^

At the same time, our results show that RT, an integral component of vRNPs, is retained within IN, NC and gRNA-depleted capsids (Figures 3 and 4). This is in general agreement with the quantitation of RT activity in IN/NC/gRNA-depleted cores isolated from class II IN mutant virions.^33^ Together, these findings establish that RT is not part of the eccentric condensates found outside the capsids of ALLINI-treated or IN-deleted virions,^7^^,^^11^^,^^33^^,^^39^^,^^60^ implicating alternative mechanisms for its incorporation into the viral core. The observation that RT fails to incorporate into and remain associated with aberrant capsid structures promoted by LEN and by hexamer cross-linking indicate that capsid lattice geometry, its curvature-, and/or proper oligomerization—during canonical capsid assembly—may play a role in RT core incorporation. The mechanisms underlying RT incorporation into HIV-1 cores will need to be fully explored in future studies.

Interestingly, “off-pathway” capsid assembly^25^ promoted by LEN and CA hexamer cross-linking also impaired the incorporation of NC into cores (Figures 5 and 6). Notably, despite the altered morphology and loss of NC, these aberrant capsids still contained IN and gRNA. These results highlight the distinct and non-redundant roles of IN and the assembling capsid structure in regulating viral core morphogenesis, including efficient IN-gRNA incorporation into flatter hexameric capsid assemblies (Figures 5 and 6), which is especially preserved in the absence of NC. Our findings reveal for the first time that NC can be stripped from the gRNA (perhaps through competitive IN-gRNA interactions), albeit during the assembly of aberrant flatter hexameric capsid lattice promoted by cross-linking CA mutations or by LEN-treatment. Future research will be needed to determine whether IN may displace or compete with NC for binding to gRNA during the normal course of HIV-1 core structural maturation.

**Figure 7:**
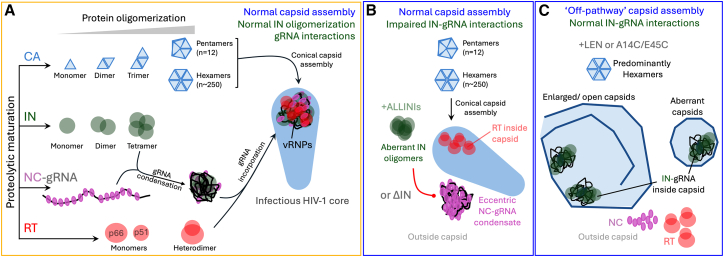
Model and consequences of IN- and capsid assembly pathways for HIV-1 core morphogenesis Following proteolytic maturation and release of individual viral proteins, protein oligomerization initiates the structural maturation aspects of HIV-1 core assembly. (A) Canonical capsid assembly progresses through mono-, di-, tri-, penta- and hexameric CA-oligomerization; pentameric and hexameric CA capsomeres polymerize to form the conical capsid shell. Simultaneously, IN oligomerization proceeds through monomer-, dimer-, and tetramerization, which is required to bind the gRNA, for its condensation and incorporation, alongside RT, and NC proteins, into capsids. Ultimately, the incorporation of all components (IN-gRNA, NC, and RT) into capsids completes assembly of the functional viral core. (B) Impairment of IN-gRNA interactions by aberrant IN-oligomerization during ALLINI-treatment, or by IN-deletion (ΔIN), leads to the exclusion of NC-gRNA from capsids, but the retention of RT during the normal pathway of capsid assembly (shown in A). (C) By contrast, alteration of capsid assembly steps by promoting hexamer formation (LEN treatment or A14C/E45C mutations) leads to the formation of flatter, aberrant, and enlarged capsids. These aberrant capsids harbor normal IN-gRNA content but fail to incorporate RT and NC proteins.

Taken together, our results highlight that HIV-1 capsid assembly and vRNP incorporation are tightly coordinated but mechanistically distinct processes (Figure 7). IN plays a central role to recruit gRNA, which chaperones NC into the core—perhaps by interacting with hexameric lattices.^12^^,^^65^ By contrast, the recruitment of RT and NC are sensitive to the proper course of capsid assembly, which progresses through the incorporation of CA-hexamers and pentamers into the conical viral core (Figure 7A). Antivirals, like ALLINIs, aberrantly oligomerize IN to impair IN-gRNA interactions.^7^^,^^33^^,^^39^ In this case, the gRNA is more efficiently bound by NC,^7^ leading to its condensation, and exclusion from capsids. Ultimately, ALLINIs result in the mislocalization of IN and NC-gRNA outside of the assembled conical capsid shell, while RT incorporation remains unaffected (Figure 7B). These findings underscore the inability of ALLINI-treated viruses to support reverse transcription in HIV-1-infected cells.^9^^,^^10^ By contrast, alteration of capsid assembly steps by promoting hexamer formation by LEN and A14C/E45C mutations impairs RT and NC incorporation into the viral core, while supporting IN-gRNA incorporation (Figure 7C).

These unexpected findings challenge the dogmatic view that RT and NC are incorporated into cores strictly through gRNA-interactions and highlights essential apposing roles of capsid and IN during HIV-1 core morphogenesis. More work will be needed to elucidate the mechanisms by which RT and NC are recruited into capsids, including how and when RT associates with the gRNA to initiate vDNA synthesis. These future studies will be needed to clarify the exact mechanisms of vRNP assembly and incorporation into capsids during infectious HIV-1 morphogenesis and how antivirals inhibit this process. We expect that the rapid affinity capture capsid purification approach described here will accelerate and enable these studies.

### Limitations of the study

Our study provides a robust and streamlined framework for isolating and interrogating HIV-1 cores. While biochemical and structural analyses identified the presence and relative enrichments of viral components, we have not comprehensively addressed the temporal dynamics or mechanistic sequence of vRNP incorporation. Similarly, the roles of non-canonical Gag cleavage intermediates and the precise mechanisms governing RT and NC incorporation remain to be delineated. Another limitation is that we have exclusively worked with lab-adapted HIV-1 strains produced from transfected HEK293T cells. Importantly, these limitations do not detract from the utility of the method; rather, they underscore its value as a foundation for future studies integrating complementary imaging, structural, and functional approaches to more fully define HIV-1 core morphogenesis.

## Resource availability

### Lead contact

Requests for further information and resources should be directed to and will be fulfilled by the lead contact, Ashwanth C. Francis (acfrancis@bio.fsu.edu).

### Materials availability

This study did not generate new unique reagents.

### Data and code availability

• All datasets generated during this study, including information required to reanalyze the data reported in this paper are available from the lead contact upon reasonable request.
• Image analysis was performed using publicly accessible open-source Fiji/ImageJ and ICY software.
• No new codes were developed.

## Acknowledgments

This work was supported by 10.13039/100000002NIH grants R01 AI181627, R01 AI148382 (to A.C.F), R21 AI174879 (to A.C.F and S.M.S.), U54 AI170701 and R01 AI157842 (to C.A.), R01 AI052014, and R37 AI039394 (to A.N.E.). The work was also supported by the Deans Post-Doctoral Fellowship to A.R. from the Dean of Arts and Science at Florida State University.

We thank Gregory Melikyan, Mariana Marin (Emory University), Sebla Kutluay (Washington University in St. Louis), and Dmitry Lyumkis (Salk Institute), for critical reading of our manuscript and for thoughtful comments. We thank Gregory Melikyan (Emory University), Mamuka Kvaratskhelia (Colorado University), Stefan Sarafianos (Emory University), Karen Kirby (Emory University), William McFadden (Emory University), Jacek Skowronski (Case Western Reserve University), Dmitry Lyumkis and Zaida Rodriguez (Salk Institute) for valuable reagents, protocols and thoughtful discussions. We thank the Biological Sciences Imaging Resource supported by Florida State University and The Southeastern Center for Microscopy of Macro Molecular Machines (SECM4) NIH R24 GM145964, NIH S10 RR025080 and S10 RR024564, for instrument access and support. We thank Dr. Huan He (Institute of Molecular Biophysics) for assistance with mass spectrometry experiments, Dr. Xiaofeng Fu (BSIR), including SECM4 staff Dr. Jiawei Li and Dr. Nebojša Bogdanović for assistance with cryo-EM data collection. We thank the NIH/NIAID AIDS reagent program and BEI resources for antibodies and recombinant protein reagents that were instrumental for this work.

## Author contributions

Conceptualization, A.C.F.; methodology, A.C.F., A.R., and J.R.A.-M., with help from S.M.S., and C.A.; investigation, A.C.F., A.R, J.R.A.-M., S.P.S, and S.E.D.; writing – original draft, A.C.F.; writing – review & editing, C.A., A.N.E., J.R.A. and A.R.; funding acquisition, A.C.F., S.M.S., C.A., and A.N.E.; supervision, A.C.F., S.M.S., and C.A.

## Declaration of interests

The authors declare no competing interests.

## STAR★Methods

### Key resources table

**Table:** 

REAGENT or RESOURCE	SOURCE	IDENTIFIER
**Antibodies**		
Anti-HIV IgG	NIH AIDS Reagent Program	Cat#ARP-3957
Anti-HIV CA	NIH AIDS Reagent Program	Cat#ARP-4121
Anti-HIV RT	NIH AIDS Reagent Program	Cat#ARP-7373
Anti-HIV IN	NIH AIDS Reagent Program	Cat#ARP-7374
Anti-HIV MA	NIH AIDS Reagent Program	Cat#ARP-4811
Anti-NC antibody	Gift (J. Skowronski)	N/A
Anti-GFP-HRP	VWR	Cat#600-103-215
Anti-mCherry antibody	Biosenses	Cat#R-1654-100

**Chemicals, peptides, and recombinant proteins**		

Dulbecco’s Modified Eagle Medium	Corning	Cat#MT10013CV
Dulbecco’s Modified Eagle Medium without phenol red	Corning	Cat#MT17205CV
Fetal Bovine Serum	Corning	Cat#MT35016CV
Penicillin-Streptomycin	Corning	Cat#MT30002CI
G418 sulfate	Corning	Cat#61-234-RG
jetPRIME DNA Transfection Reagent	VWR	Cat#89129-924
Lenti-X concentrator	Takara Bio	Cat #631231
Raltegravir	NIH AIDS Reagent Program	Cat#HRP-11680
Saquinavir	NIH AIDS Reagent Program	Cat#HRP-4658
KF116 (ALLINI)	Kind Gift from Dr. Mamuka Kvaratskhelia (University of Colorado)	Koneru et al.^58^
Lenacapavir	Kind Gift from Dr. Mamuka Kvaratskhelia (University of Colorado)	Faysal et al.^21^; Huang et al.^26^
Protein A Magnetic Beads	Thermo Fisher Scientific	Cat#10001D
IP6 (Phytic acid)	TCI Chemicals	Cat# P0409
Cyclosporin A (CsA)	Thermo Scientific	Cat#J63191.03

**Critical commercial assays**		

Bright-Glow Luciferase Assay System	Promega	Cat#E2620
HIV1 p24 ELISA kit	Abcam	Cat#ab218268

**Experimental models: Cell lines**		

Lenti-X HEK293T cells	Takara Bio	Cat#632180; RRID:CVCL_4401
CPSF6-CRISPR/Cas9 knockout HEK293T cells (CKO cells)	Sowd et al.^66^	N/A
TZM-bl cells	NIH AIDS Reagent Program	Cat#ARP-8129; RRID:CVCL_B478

**Oligonucleotides**		

MSS Fwd: GCCTCAATAAAGCTTGCCTTGA	Christensen et al.^23^; Sowd et al.^52^	N/A
MSS Rev: TGACTAAAAGGGTCTGAGGGATCT	Christensen et al.^23^; Sowd et al.^52^	N/A
FLM Fwd: CTAGAACGATTCGCAGTTAATCCT	Christensen et al.^23^; Sowd et al.^52^	N/A
FLM Rev: CTATCCTTTGATGCACACAATAGAG	Christensen et al.^23^; Sowd et al.^52^	N/A

**Recombinant DNA**		

pR9ΔEnv	Francis et al.^38^	N/A
pNLxΔEnv	Francis et al.^38^	N/A
pR9ΔEnv CA A14C/E45C	This study	N/A
pHIVeGFPΔEnv	Francis et al.^38^	N/A
pYFP-Vpr	Sood et al.^56^	N/A
pVpr-IN-mNeonGreen	Francis et al.^45^^,^^47^	N/A
VSV-G envelope plasmid	Francis et al.^38^	N/A
CypA-DsRed (CDR)	Francis et al.^38^	N/A
pNLxΔEnvΔIN	Kessl et al.^7^; Engelman et al.^8^	N/A
pNLxΔEnvΔRTΔIN	Kessl et al.^7^; Engelman et al.^8^	N/A

**Software and algorithms**		

Fiji/ImageJ	Open source	RRID:SCR_002285
ICY image analysis software	Institut Pasteur, Open source de Chaumont et al.^67^	RRID:SCR_010587

### Experimental model and study participant details

All cell lines have been authenticated by short tandem repeat (STR) profiling and tested negative for mycoplasma contamination.

#### Cell lines

Lenti-X HEK293T (TaKaRa Bio, Cat#632180), CPSF6-CRISPR/Cas9 Knockout (CKO) HEK293T cells^66^ and Tzmbl cells were grown in complete high-glucose Dulbecco’s Modified Eagle Medium (DMEM, Corning, Cat#MT10013CV) supplemented with 10% Fetal Bovine Serum (FBS, Corning, Cat#MT35016CV), 100 U/mL penicillin-streptomycin (Corning, Cat#MT30002CI), and 0.5 mg/mL G418 sulfate (Corning, Cat#61-234-RG). All cells were maintained at 37°C in a humidified incubator with 5% CO_2_.

No animals or human participants were involved in this study.

### Method details

#### Plasmid vectors and antivirals

Plasmids encoding HIV-1 vector sequences (pR9ΔEnv, pR9ΔEnv CA A14C/E45C, pNLxΔEnv and pHIVeGFPΔEnv), CypA-DsRed (CDR), pVpr-IN-mNeonGreen (INmNG), and vesicular stomatitis virus envelop glycoprotein G (VSV-G) were described previously.^38^^,^^45^^,^^47^ Plasmid pYFP-Vpr was described in.^56^ Plasmids with truncated Pol proteins, pNLxΔEnvΔIN and pNLxΔEnvΔRTΔIN, described in,^7^ ALLINI KF116(−) described in,^58^ and Lenacapavir described in^18^ were kind gifts from Dr. Mamuka Kvaratskhelia, University of Colorado, CO. All plasmid sequences were verified by whole plasmid sequencing (Primordium, USA). The IN-strand transfer inhibitor Raltegravir (RAL, HRP-11680) and protease inhibitor Saquinavir (SAQ, HRP-4658) were obtained through BEI-Resources, NIAID.

#### Pseudovirus production and characterization

This protocol is adapted from.^38^ Briefly, on day-1, HEK293T producer cells were seeded in 6-well plates at 80% confluency. On day-2, the cells were transfected with 2 μg of plasmids expressing the HIV-backbone (pR9ΔEnv, pR9ΔEnv CA A14C/E45C, pHIVeGFPΔEnv or pNLxΔEnv, including ΔIN and ΔRTΔIN) for capsid capture experiments. For infectivity experiments, where indicated 2 μg of HIV-backbone and 0.5 μg of VSV-G plasmids were co-transfected. Fluorescently labeled viruses were generated using the same protocol, by co-transfecting pR9 or pHIVeGFPΔEnv (2 μg) with 0.8 μg of pYFP-Vpr or pVpr-INmNG. The indicated concentration of plasmids were mixed in 200 μL JetPrime buffer and 6 μL JetPrime reagent (VWR, Cat#89129–924) and incubated for 13 min, followed by addition to HEK293T cells. After 6 h, the medium was replaced with 2 mL of fresh complete DMEM without phenol red (Corning, Cat#MT17205CV). Where indicated drugs (SAQ, KF116, RAL, or LEN) were added at the indicated concentrations during this change of media. Transfected cells were further incubated for 36 h in a cell-culture incubator maintained at 37°C and supplemented with 5% CO_2._ On day-4, 2 mL viral supernatants were collected, filtered through a 0.45 μm filter, and concentrated using the polyethylene glycol-based Lenti-X concentrator (Takara, Cat#631231) to remove serum, antivirals, and other impurities from cellular media. Concentrated virus preparations were quantified for RT activity (RTU) using the SG-PERT protocol^68^ or for CA/p24 content by ELISA, aliquoted, and stored at −80°C until use. Fresh stocks of LEN (1 mM), SAQ (10 mM), KF116 (10 mM), and RAL (10 mM) compounds in DMSO were thawed and used for each experiment. SAQ and KF116 were used at 1 μM and RAL was used at 10 μM final concentrations, whereas a titration of LEN was used as indicated. Comparable volumes of DMSO were used as vehicle controls.

#### Infectivity assays

Ten thousand TZM-bl cells were plated in triplicate wells of a 96-well plate and infected with normalized input of serially diluted (5-fold) virus supernatants. VSV-G pseudotyped HIV-1 produced in the presence of inhibitors was normalized by using equal RTU (KF116, RAL, LEN and DMSO- treated virus) or CA/p24 content (pNLx WT, pNLx-ΔIN and ΔRTΔIN viruses). Virus infection was enhanced by 30 min centrifugation at 1,500×g at 16°C. Cells were incubated at 37°C in a CO_2_ incubator for an additional 48 h, lysed, and luciferase activity was measured using the Bright-Glow luciferase substrate (Promega), as per the manufacturer’s protocol.

#### Cytosol extraction of CDR protein

CKO cells were used for CDR preparation. The rationale was to capture capsids independently of CPSF6, which engages the FG pocket of capsid^17^ with similar high affinity as CDR^69^ and is not released by CsA. CKO cells were seeded in 6-well plates at 80% confluency and transfected with 2 μg of CDR plasmid using JetPrime transfection reagent, as above. Six h post-transfection, the medium was replaced with 2 mL of fresh DMEM supplemented with 10% FBS and lacking phenol red. Cells were incubated for an additional 36 h at 37°C in a 5% CO_2_ incubator. After incubation, the cellular media was discarded, and the cells were washed twice with PBS via centrifugation at 1600xg for 5 min.

Subsequently, CDR-containing cytosol extraction was performed as described previously.^38^ In brief, cell pellets were permeabilized using 100 μg/mL digitonin in the presence of protease inhibitor cocktail (Cell Signaling Technology, Cat#5871 S) using the following mixture: 490 μL PBS, 5 μL of 10 mg/mL digitonin, and 5 μL of 100× protease inhibitors. The cells were incubated on ice for 30 min with occasional vortexing. After incubation, the supernatant containing CDR-cytosol, which appeared as a slightly pink solution, was collected after pelleting cellular debris at 5,000 xg for 5 min. The concentration of CDR was determined by comparison to a standard curve constructed using recombinant mCherry protein (Abcam, Cat#ab199750) and a Tecan Plate-reader. Extracted cytosols consistently contained 50–100 ng/μL CDR protein. The CDR-containing cytosol was aliquoted and stored at −80°C until further use.

#### CDR binding to fluorescent HIV 1 cores immobilized on glass

Fluorescent INmNG-labelled HIV-1 particles (WT mature or immature (+SAQ) virions or G89V capsid mutant) were immobilized onto a poly-L-lysine coated glass surface of a 8-well chambered slide for 30 min at 4°C. Slides were washed twice with Dulbecco’s PBS to remove unbound viruses, and the immobilized particles were permeabilized on the microscope stage by adding detergent Saponin (SAP, 100 μg/mL). Subsequently, CDR binding (50 nM) to INmNG-labeled cores was visualized on a Leica SP8 laser scanning confocal microscope using a C-Apo 63×/1.4NA oil-immersion objective. Adaptive focus control (Leica Microsystems) was utilized to maintain focus during data collection and to correct for axial drift in 2D images. Time series image datasets were collected at 512 × 512 frame size, 180 nm pixel sizes (2× digital zoom) with 1.54 μs pixel dwell times, 2-line averaging, and at 5 s intervals. Images were collected using 488 and 561 nm laser lines to excite the fluorophores with respective emissions collected between 502 and 560 nm (INmNG) and 572–630 nm (CDR) using GaSP-HyD detectors. 2D-time series images were processed offline using ICY image analysis software.

#### Antibodies

The following antibodies were tested for the affinity capture of HIV-1 cores: rat polyclonal anti-RFP antibody (Cat#104272–05, and 104272–07, from Chromotek), rabbit polyclonal antibody to mCherry (Cat #R-1654-100, from Biosenses) and (Cat#26765-1-AP, Proteintech). The following primary antibodies, obtained through BEI Resources, NIAID, were used at the indicated dilutions: anti-HIV-IgG (Polyclonal Anti-HIV Immune Globulin, Pooled Inactivated Human Sera, ARP-3957 contributed by NABI and National Heart Lung and Blood Institute (Dr. Luiz Barbosa), RRID:AB_2890264; 1:2000), anti-CA^70^ (Monoclonal Anti- HIV-1 p24 (AG3.0), ARP-4121, contributed by Dr. Marie-Claire Gauduin, RRID:AB_2734137; 1:1000), anti-RT (Monoclonal Anti-HIV-1 Reverse Transcriptase (8C4), ARP-7373; 1:1000), anti-IN (Monoclonal Anti- HIV-1 Integrase (2C11), ARP-7374; 1:1000), and anti-MA^71^ (Polyclonal Anti-HIV-1 p17 Protein (antiserum, Rabbit), ARP-4811, contributed by Dr. Paul Spearman and Dr. Lingmei Ding; 1:2000). The anti-NC antibody (1:5000) was a kind gift from Jacek Skowronski (Case Western Reserve University) and the primary anti-GFP antibody conjugated to HRP (for YFP-Vpr) was purchased from VWR (Cat#600-103-215, 1:1000). Secondary antibodies, anti-human conjugated to HRP (Cat#A18847, Invitrogen 1:2000), anti-mouse conjugated to HRP (Cat#AC2115 Azure Biosystems Inc. 1:5000), and anti-rabbit conjugated to HRP (Cat#4050–05, Southern Biotech 1:1000), were obtained from commercial vendors.

#### Affinity capture of native capsids from membrane-permeabilized virions

Following preliminary testing, for the remainder of studies we used rabbit polyclonal antibody to mCherry (Biosenses, Cat#R-1654-100) to capture CDR onto Protein A-coated beads, which showed optimal performance versus other tested antibodies (Figure S1B).

Step 1 Bead Calibration – A 1.5 mL Eppendorf tube containing 15 μL of Protein A magnetic beads (DynaBeads Protein A, Thermo Fisher Scientific, Cat#10001D or #10002D) was placed on a magnetic rack, and the supernatant was carefully removed using a pipette. The 15 μL bead volume was chosen for capsid capture as it yielded HIV-1 protein signals of optimal intensity, neither too faint nor overly saturated, in subsequent immunoblot analysis. The beads were washed once with 100 μL of PBS by gentle tapping, followed by magnetic separation to remove the wash buffer. Next, the beads were washed a second time with 3% BSA in PBS, to block non-specific interactions, and subsequently washed twice more with PBS alone (total 3× washes).

Step 2 Antibody binding – To conjugate the antibody, 15 μL of PBS (equal to the initial bead volume) and 0.5 μL of antibody solution (1 mg/mL) were added to the washed beads. The mixture was incubated with end-over-end rotation for 15 min at room temperature. After incubation, the beads were washed twice with PBS by magnetic separation to remove unbound antibody.

Step 3 Capture of CDR on antibody coated magnetic beads: The antibody-coated beads were incubated with 15 μL of PBS and 1 μL of cytosol containing ∼50–100 ng of CDR for 30 min at room temperature with end-over-end rotation (or by occasional tapping). Following incubation, the beads were thoroughly washed five times with 100 μL of PBS to remove any unbound CDR and other cellular contaminants.

Step 4 Affinity capture of HIV-1 capsids on CDR-coated beads: An aliquot of 2–5 μL of 10× concentrated virus preparation (∼5 RTU or 100–250 ng of CA/p24) was placed in a separate tube. IP6 (final concentration 100 μM in PBS) and TX-100 (final concentration 0.5% in PBS) were added to permeabilize the viral envelope and expose viral capsids.

The following mixture was typically used for capsid capture (CC): 5 μL of 10× concentrated R9 virus mixed with permeabilizing solution containing: 17.4 μL PBS, 2.8 μL of 1 mM IP6, and 2.8 μL of 5% TX-100, to make a total volume of 28 μL. The permeabilized virus mixture was then added to CDR-coated beads separated and incubated at room temperature for 5–10 min, with occasional tapping to prevent bead settling. After incubation, the supernatant was discarded, and the beads were washed three times with 100 μL wash buffer (PBS containing 100 μM IP6). For immunoblotting experiments, CC-containing beads were analyzed directly (without CsA-mediated elution).

Step 5 CC elution for structural studies: The volume of the elution buffer was adjusted depending on the concentration of capsids required for downstream applications. For EM studies, CCs from 15 μL of viral supernatant (250 ng p24) were eluted from 75 μL of CDR-coated beads by treatment with 20 μL elution buffer (10 μM CsA, 100 μM IP6 in PBS) for 5 min with thorough mixing by pipetting up and down. A fresh aliquot of CsA (stock at 50 mM in DMSO) was used each time.

#### CDR-capture of capsids from infected cells

On day-1, HEK293T producer cells were seeded in a 6-well plate at 80% confluency. On day-2, cells were transduced with 10× concentrated VSV-G pseudotyped WT or capsid mutant E45A or K203A viruses (∼40 RTU per well, multiplicity of infection (MOI) ∼40). The plate was centrifuged at 1,500 × g for 30 min at room temperature to enhance virus binding and subsequently incubated under standard culture conditions (37°C, 5% CO_2_) to synchronize entry into cells. 4 h after transduction, the culture media were aspirated, and 500 μL of 1× trypsin was added to each well to remove membrane-bound virus and detach cells. After a 5-min incubation at 37°C, 1 mL of DMEM containing FBS was added to neutralize the trypsin. The cells were collected into 2 mL microcentrifuge tubes, centrifuged, and washed three times with ice-cold PBS. The cell pellets were resuspended in 400 μL of hypotonic lysis buffer (10 mM Tris-HCl pH 8.0, 10 mM KCl, 1 mM EDTA) with protease inhibitor (Protease Inhibitor Cocktail, Cat # 5871 S, Cell Signaling Technology). The suspensions were incubated on ice for 15 min, followed by mild vortexing for 30 s to facilitate cell lysis. Cellular debris was removed by centrifugation at 1,000 × g for 7 min at 4°C, and the supernatants (total lysates) were collected. For the capsid capture assay, 360 μL of cell lysate was combined with 40 μL of 1 mM IP6 (10× stock) to achieve the desired final working 100 μM IP6. Capsids were subsequently captured using 15 μL of CDR bound magnetic beads, as above.

#### Capsid content analysis by immunoblot

Input virus (one-fifth of the amount used in the CC experiment) and 15 μL of CC sample, corresponding to ∼1 RTU, were loaded onto an SDS–polyacrylamide gel (Mini-PROTEAN TGX Stain-Free Gels, 4–15% 12 well comb, Cat # 4568085, BioRad) for electrophoresis (SDS–PAGE) and transferred to a PVDF membrane (Cat # 1620219, BioRad). Membrane was blocked with 5% skim milk (Cat #C6961, Hardy diagnostics) in PBS containing 0.025% Tween 20 and incubated with the indicated primary antibodies. After incubation, membranes were washed three times with PBST (PBS with 0.05% Tween 20; Cat #76371–736, VWR), followed by incubation with a horseradish peroxidase (HRP)-conjugated secondary antibody. Signal detection was performed using Immobilon Western Chemiluminescent HRP Substrate (Cat#WBKLS0050, Millipore), and images were acquired using a Bio-Rad imaging system (Biorad, ChemiDoc MP Imaging system). Unsaturated immunoblots were quantified using Fiji/ImageJ software version 2.16.0/1.54n. Protein band densitometry was normalized to the respective internal control (WT virions or WT CC); for RT, only the p66 subunit, which was the dominant signal on blots, was quantified and plotted.

#### Quantitative PCR for gRNA analysis

Equal (∼1 RTU) input of virions and CC products were placed in separate Eppendorf tubes, and an equal volume of 2× lysis buffer (0.25% Triton X-100, 50 mM KCl, 100 mM TrisHCl pH 7.4, 40% glycerol with RNAse inhibitor) was added to each. The samples were centrifuged at 5,000 × g for 10 min at room temperature to complete the lysis. Following lysis, each sample was brought to a final volume of 100 μL with molecular-grade water and vortexed for 10 s to ensure homogeneity. From the diluted lysates, 2.5 μL was transferred to a 96-well PCR plate and mixed with an equal volume of 2× reaction buffer (SYBR green master mix with 0.2 mg/mL BSA, 1 μM forward primer, 1 μM reverse primer, 2000 units/mL M-MuLV Reverse Transcriptase with RiboLock RNase Inhibitor). Minus-strand strong stop (MSS) primers were used to amplify gRNA (MSS fwd: 5’ – GCCTCAATAAAGCTTGCCTTGA – 3′, and MSS rev: 5’ – TGACTAAAAGGGTCTGAGGGATCT – 3′). The qPCR was performed under the following cycling conditions: [42°C 20 min, 95°C 2 min, 95°C 5 s/55°C 5 s/72°C 15 s (40 cycles), 82°C 5 s]. Quantification cycle (Cq) values were determined and compared to a standard curve generated using MS2 RNA templates, which relates Cq values to RNA copy number.

#### ERT assay

ERT reactions^23^^,^^52^ were carried out for virions, CC, or CsA-eluted cores, with equal input normalized based on RT activity (∼1 RTU). Where indicated, 0.1 mM (final concentrations) of each dNTP or ddH_2_O (no dNTPs) was added to the input sample to make a final volume of 25 μL. ERT reactions were initiated by the addition of equal volume (25 μL) of a 2× ERT reaction buffer (40 mM Tris-HCl (pH 7.6), 2 mg/mL BSA, 300 mM NaCl, 4 mM MgCl_2_, 2 mM dithiothreitol (DTT), 20 μM IP_6_, and 0.2% Triton X-100) and incubated at 37°C for 16 h. The reverse transcribed DNA was purified using PCR clean-up columns and used for qPCR amplification of ERT products. Early and late reverse transcription products were quantified using MSS (above) and full-length minus strand (FLM) [FLM fwd: 5’ – CTAGAACGATTCGCAGTTAATCCT – 3′, and FLM rev 5’ – CTATCCTTTGATGCACACAATAGAG – 3’] primer sets, respectively. qPCR was performed under the following cycling conditions: 95°C 1 min, [95°C 15 s/60°C 30 s] (40 cycles). Cq values were determined and compared to a standard curve generated using serial dilution of proviral pR9-plasmid DNA templates, which relates Cq values to DNA copy number. ERT of pelleted LEN-treated and A14C/E45C cores were performed as described previously.^53^^,^^54^

#### Electron microscopy of eluted capsids

For negative stain EM, CsA-eluted cores (5 μL or ∼100 ng of CA/p24) were applied to glow-discharged gold grids with lacey carbon with a continuous layer of 2 nm carbon film (EMS #LC200-Au-CC, Electron Microscopy Sciences, Hatfield, PA) and incubated for 2 min. The grid with core samples was blotted and stained with 2% uranyl acetate for 2 min and blotted dry. EM was performed using a Hitachi HT7800 transmission electron microscope (Hitachi High-Tech, Tokyo, Japan) at 120 kV at 30,000–50,000× magnification.

For cryo-EM studies, glow-discharged gold grids (lacey carbon with a continuous layer of 2 nm carbon film (EMS #LC200-Au-CC, Electron Microscopy Sciences, Hatfield, PA) with core samples were plunge frozen in liquid ethane using a Gatan CryoPlunger 3 (CP3) semi-automatic blotter, using a backside blotting scheme.^24^ The grids were subsequently clipped and examined using a Titan Krios 300 KeV cryo-transmission electron microscope. Micrographs were collected at 37,000× magnification, pixel sizes 1.23 Å using a total dose of 45.00 e^–^/Å^2^ at −3 μm defocus.

For Cryo-ET studies, cryo-grids were transferred to a 300 keV Titan Krios transmission electron microscope (Thermo Fisher Scientific) equipped with a DE Apollo direct detector. Cryo-ET tilt-series datasets were collected at a magnification of 22,500×, corresponding to a nominal pixel size of 1.98 Å at a defocus of −10 μm. Tilt-series were acquired using the Leginon software. Each tilt series was collected using a sequential acquisition scheme over a tilt range of −63° to +63° with 2° increments. The total accumulated electron dose per tilt series was 70.4 e^−^/Å^2^. Collected tilt series were processed directly from tilt images. Tilt series alignment, contrast transfer function (CTF) correction, and tomogram reconstruction were automatically performed using AreTomo3 software.^72^ Tomograms were reconstructed by weighted back-projection (WBP) at a binning factor of 2, resulting in a final pixel size of 3.96 Å. Cryo-EM/ET images were analyzed on ImageJ (Fiji) image analysis software for morphology classification, dimension measurements, and particle counting, by 2 independent operators.

### Quantification and statistical analysis

Statistical analysis, unpaired Student’s t test was performed using GraphPad Prism software v. 10.6.1. The statistical method used, including samples size are indicated in associated figure legends. Significance, *p*-values <0.05, ∗; <0.01, ∗∗, and <0.001∗∗∗, *p* > 0.05 not-significant and denoted in figure panels, and legends.

## Supplemental information

Document S1. Figures S1–S10

Table S1. Peptide enrichment analysis by mass spectrometry, related to Figure S4

Data S1. Uncropped western blots of main and supplementary Figures

## References

[1] Freed E.O. (2015). HIV-1 assembly, release and maturation. Nat. Rev. Microbiol..

[2] Sumner C., Ono A. (2024). The “basics” of HIV-1 assembly. PLoS Pathog..

[3] Sundquist W.I., Kräusslich H.G. (2012). HIV-1 Assembly, Budding, and Maturation. Cold Spring Harb. Perspect. Med..

[4] Rein A. (2019). RNA Packaging in HIV. Trends Microbiol..

[5] de Marco A., Müller B., Glass B., Riches J.D., Kräusslich H.G., Briggs J.A.G. (2010). Structural analysis of HIV-1 maturation using cryo-electron tomography. PLoS Pathog..

[6] Briggs J.A.G., Wilk T., Welker R., Kräusslich H.G., Fuller S.D. (2003). Structural organization of authentic, mature HIV-1 virions and cores. EMBO J..

[7] Kessl J.J., Kutluay S.B., Townsend D., Rebensburg S., Slaughter A., Larue R.C., Shkriabai N., Bakouche N., Fuchs J.R., Bieniasz P.D., Kvaratskhelia M. (2016). HIV-1 Integrase Binds the Viral RNA Genome and Is Essential during Virion Morphogenesis. Cell.

[8] Engelman A., Englund G., Orenstein J.M., Martin M.A., Craigie R. (1995). Multiple effects of mutations in human immunodeficiency virus type 1 integrase on viral replication. J. Virol..

[9] Engelman A.N., Kvaratskhelia M. (2022). Multimodal Functionalities of HIV-1 Integrase. Viruses.

[10] Elliott J.L., Kutluay S.B. (2020). Going beyond Integration: The Emerging Role of HIV-1 Integrase in Virion Morphogenesis. Viruses.

[11] Fontana J., Jurado K.A., Cheng N., Ly N.L., Fuchs J.R., Gorelick R.J., Engelman A.N., Steven A.C. (2015). Distribution and Redistribution of HIV-1 Nucleocapsid Protein in Immature, Mature, and Integrase-Inhibited Virions: a Role for Integrase in Maturation. J. Virol..

[12] Singer M.R., Li Z., Rey J.S., Hope J., Chenavier F., Cook N.J., Punch E., Smith J., Zhou Z., Maslen S. (2026). Integrase anchors viral RNA to the HIV-1 capsid interior. Nature.

[13] Bester S.M., Wei G., Zhao H., Adu-Ampratwum D., Iqbal N., Courouble V.V., Francis A.C., Annamalai A.S., Singh P.K., Shkriabai N. (2020). Structural and mechanistic bases for a potent HIV-1 capsid inhibitor. Science.

[14] Singh K., Gallazzi F., Hill K.J., Burke D.H., Lange M.J., Quinn T.P., Neogi U., Sönnerborg A. (2019). GS-CA Compounds: First-In-Class HIV-1 Capsid Inhibitors Covering Multiple Grounds. Front. Microbiol..

[15] Link J.O., Rhee M.S., Tse W.C., Zheng J., Somoza J.R., Rowe W., Begley R., Chiu A., Mulato A., Hansen D. (2020). Clinical targeting of HIV capsid protein with a long-acting small molecule. Nature.

[16] Bhattacharya A., Alam S.L., Fricke T., Zadrozny K., Sedzicki J., Taylor A.B., Demeler B., Pornillos O., Ganser-Pornillos B.K., Diaz-Griffero F. (2014). Structural basis of HIV-1 capsid recognition by PF74 and CPSF6. Proc. Natl. Acad. Sci. USA.

[17] Price A.J., Jacques D.A., McEwan W.A., Fletcher A.J., Essig S., Chin J.W., Halambage U.D., Aiken C., James L.C. (2014). Host cofactors and pharmacologic ligands share an essential interface in HIV-1 capsid that is lost upon disassembly. PLoS Pathog..

[18] Wei G., Iqbal N., Courouble V.V., Francis A.C., Singh P.K., Hudait A., Annamalai A.S., Bester S., Huang S.W., Shkriabai N. (2022). Prion-like low complexity regions enable avid virus-host interactions during HIV-1 infection. Nat. Commun..

[19] Deshpande A., Bryer A.J., Andino-Moncada J.R., Shi J., Hong J., Torres C., Harel S., Francis A.C., Perilla J.R., Aiken C., Rousso I. (2024). Elasticity of the HIV-1 core facilitates nuclear entry and infection. PLoS Pathog..

[20] Faysal K.M.R., Walsh J.C., Renner N., Márquez C.L., Shah V.B., Tuckwell A.J., Christie M.P., Parker M.W., Turville S.G., Towers G.J. (2024). Pharmacologic hyperstabilisation of the HIV-1 capsid lattice induces capsid failure. eLife.

[21] Briganti L., Annamalai A.S., Bester S.M., Wei G., Andino-Moncada J.R., Singh S.P., Kleinpeter A.B., Tripathi M., Nguyen B., Radhakrishnan R. (2025). Structural and mechanistic bases for resistance of the M66I capsid variant to lenacapavir. mBio.

[22] Li C., Burdick R.C., Siddiqui R., Janaka S.K., Hsia R.C., Hu W.S., Pathak V.K. (2025). Lenacapavir disrupts HIV-1 core integrity while stabilizing the capsid lattice. Proc. Natl. Acad. Sci. USA.

[23] Christensen D.E., Ganser-Pornillos B.K., Johnson J.S., Pornillos O., Sundquist W.I. (2020). Reconstitution and visualization of HIV-1 capsid-dependent replication and integration in vitro. Science.

[24] Rodriguez Z.K., Andino-Moncada J.R., Buth S.A., Mehrani A., Ranaweera A., Shi J., Andrade L.R., Singh S.P., Strutzenberg T.S., Marin M. (2025). Time-Resolved Fluorescence Imaging and Correlative Cryo-Electron Tomography to Study Structural Changes of the HIV-1 Capsid. ACS Nano.

[25] Gupta M., Waltmann C., Renner N., Wang Y., James L., Jacques D.A., Böcking T., Voth G.A. (2026). Mechanistic Insights into Lenacapavir-Induced Off-Pathway HIV-1 Capsid Assembly. Proc. Natl. Acad. Sci. USA..

[26] Huang S.W., Briganti L., Annamalai A.S., Greenwood J., Shkriabai N., Haney R., Armstrong M.L., Wempe M.F., Singh S.P., Francis A.C. (2025). The primary mechanism for highly potent inhibition of HIV-1 maturation by lenacapavir. PLoS Pathog..

[27] Kelley C.F., Acevedo-Quiñones M., Agwu A.L., Avihingsanon A., Benson P., Blumenthal J., Brinson C., Brites C., Cahn P., Cantos V.D. (2025). Twice-Yearly Lenacapavir for HIV Prevention in Men and Gender-Diverse Persons. N. Engl. J. Med..

[28] McKellar M.S. (2025). Lenacapavir: a first-in-class capsid inhibitor for HIV treatment and prevention. Curr. Opin. Infect. Dis..

[29] Jamey N., Udayanga D.M.N., Zhang H., Ravichandran S.M., Cai X., Lorson Z.C., McFadden W.M., Ryu W.H., Katekar R., Xie J. (2025). Design, synthesis and profiling of highly potent antivirals targeting emerging drug-resistant HIV-1 variants. bioRxiv.

[30] Kirby K.A., McFadden W.M., Wang L., Du H., Zhang H., Castaner A.E., Lorson Z.C., Nafisi A., Luchsinger C., Hachiya A. (2025). Structural, biophysical, and virological mechanistic characterization of HIV-1 capsid-targeting antivirals. bioRxiv.

[31] Forshey B.M., Aiken C. (2003). Disassembly of human immunodeficiency virus type 1 cores in vitro reveals association of Nef with the subviral ribonucleoprotein complex. J. Virol..

[32] Forshey B.M., von Schwedler U., Sundquist W.I., Aiken C. (2002). Formation of a human immunodeficiency virus type 1 core of optimal stability is crucial for viral replication. J. Virol..

[33] Elliott J.L., Eschbach J.E., Koneru P.C., Li W., Puray-Chavez M., Townsend D., Lawson D.Q., Engelman A.N., Kvaratskhelia M., Kutluay S.B. (2020). Integrase-RNA interactions underscore the critical role of integrase in HIV-1 virion morphogenesis. eLife.

[34] Welker R., Hohenberg H., Tessmer U., Huckhagel C., Kräusslich H.G. (2000). Biochemical and structural analysis of isolated mature cores of human immunodeficiency virus type 1. J. Virol..

[35] Accola M.A., Ohagen A., Göttlinger H.G. (2000). Isolation of human immunodeficiency virus type 1 cores: retention of Vpr in the absence of p6(gag). J. Virol..

[36] Paxton W., Connor R.I., Landau N.R. (1993). Incorporation of Vpr into human immunodeficiency virus type 1 virions: requirement for the p6 region of gag and mutational analysis. J. Virol..

[37] Francis A.C., Cereseto A., Singh P.K., Shi J., Poeschla E., Engelman A.N., Aiken C., Melikyan G.B. (2022). Localization and functions of native and eGFP-tagged capsid proteins in HIV-1 particles. PLoS Pathog..

[38] Francis A.C., Marin M., Shi J., Aiken C., Melikyan G.B. (2016). Time-Resolved Imaging of Single HIV-1 Uncoating In Vitro and in Living Cells. PLoS Pathog..

[39] Desimmie B.A., Schrijvers R., Demeulemeester J., Borrenberghs D., Weydert C., Thys W., Vets S., Van Remoortel B., Hofkens J., De Rijck J. (2013). LEDGINs inhibit late stage HIV-1 replication by modulating integrase multimerization in the virions. Retrovirology.

[40] Pornillos O., Ganser-Pornillos B.K., Banumathi S., Hua Y., Yeager M. (2010). Disulfide bond stabilization of the hexameric capsomer of human immunodeficiency virus. J. Mol. Biol..

[41] Ni T., Zhu Y., Yang Z., Xu C., Chaban Y., Nesterova T., Ning J., Böcking T., Parker M.W., Monnie C. (2021). Structure of native HIV-1 cores and their interactions with IP6 and CypA. Sci. Adv..

[42] Briggs J.A.G., Simon M.N., Gross I., Kräusslich H.G., Fuller S.D., Vogt V.M., Johnson M.C. (2004). The stoichiometry of Gag protein in HIV-1. Nat. Struct. Mol. Biol..

[43] Mallery D.L., Márquez C.L., McEwan W.A., Dickson C.F., Jacques D.A., Anandapadamanaban M., Bichel K., Towers G.J., Saiardi A., Böcking T., James L.C. (2018). IP6 is an HIV pocket factor that prevents capsid collapse and promotes DNA synthesis. eLife.

[44] Dick R.A., Zadrozny K.K., Xu C., Schur F.K.M., Lyddon T.D., Ricana C.L., Wagner J.M., Perilla J.R., Ganser-Pornillos B.K., Johnson M.C. (2018). Inositol phosphates are assembly co-factors for HIV-1. Nature.

[45] Francis A.C., Marin M., Singh P.K., Achuthan V., Prellberg M.J., Palermino-Rowland K., Lan S., Tedbury P.R., Sarafianos S.G., Engelman A.N., Melikyan G.B. (2020). HIV-1 replication complexes accumulate in nuclear speckles and integrate into speckle-associated genomic domains. Nat. Commun..

[46] Shah V.B., Aiken C. (2011). In vitro uncoating of HIV-1 cores. J. Vis. Exp..

[47] Francis A.C., Melikyan G.B. (2018). Single HIV-1 Imaging Reveals Progression of Infection through CA-Dependent Steps of Docking at the Nuclear Pore, Uncoating, and Nuclear Transport. Cell Host Microbe.

[48] Fricke T., Brandariz-Nuñez A., Wang X., Smith A.B., Diaz-Griffero F. (2013). Human cytosolic extracts stabilize the HIV-1 core. J. Virol..

[49] Kutluay S.B., Perez-Caballero D., Bieniasz P.D. (2013). Fates of retroviral core components during unrestricted and TRIM5-restricted infection. PLoS Pathog..

[50] Stremlau M., Perron M., Lee M., Li Y., Song B., Javanbakht H., Diaz-Griffero F., Anderson D.J., Sundquist W.I., Sodroski J. (2006). Specific recognition and accelerated uncoating of retroviral capsids by the TRIM5alpha restriction factor. Proc. Natl. Acad. Sci. USA.

[51] Hou Z., Shen Y., Fronik S., Shen J., Shi J., Xu J., Chen L., Hardenbrook N., Engelman A.N., Aiken C., Zhang P. (2025). HIV-1 nuclear import is selective and depends on both capsid elasticity and nuclear pore adaptability. Nat. Microbiol..

[52] Sowd G.A., Shi J., Aiken C. (2021). HIV-1 CA Inhibitors Are Antagonized by Inositol Phosphate Stabilization of the Viral Capsid in Cells. J. Virol..

[53] Jennings J., Shi J., Varadarajan J., Jamieson P.J., Aiken C. (2020). The Host Cell Metabolite Inositol Hexakisphosphate Promotes Efficient Endogenous HIV-1 Reverse Transcription by Stabilizing the Viral Capsid. mBio.

[54] Jennings J., Bracey H., Hong J., Nguyen D.T., Dasgupta R., Rivera A.V., Sluis-Cremer N., Shi J., Aiken C. (2024). The HIV-1 capsid serves as a nanoscale reaction vessel for reverse transcription. PLoS Pathog..

[55] Desai T.M., Marin M., Sood C., Shi J., Nawaz F., Aiken C., Melikyan G.B. (2015). Fluorescent protein-tagged Vpr dissociates from HIV-1 core after viral fusion and rapidly enters the cell nucleus. Retrovirology.

[56] Sood C., Francis A.C., Desai T.M., Melikyan G.B. (2017). An improved labeling strategy enables automated detection of single-virus fusion and assessment of HIV-1 protease activity in single virions. J. Biol. Chem..

[57] Lanman J., Lam T.T., Emmett M.R., Marshall A.G., Sakalian M., Prevelige P.E. (2004). Key interactions in HIV-1 maturation identified by hydrogen-deuterium exchange. Nat. Struct. Mol. Biol..

[58] Koneru P.C., Francis A.C., Deng N., Rebensburg S.V., Hoyte A.C., Lindenberger J., Adu-Ampratwum D., Larue R.C., Wempe M.F., Engelman A.N. (2019). HIV-1 integrase tetramers are the antiviral target of pyridine-based allosteric integrase inhibitors. eLife.

[59] Mouscadet J.F., Tchertanov L. (2009). Raltegravir: molecular basis of its mechanism of action. Eur. J. Med. Res..

[60] Jurado K.A., Wang H., Slaughter A., Feng L., Kessl J.J., Koh Y., Wang W., Ballandras-Colas A., Patel P.A., Fuchs J.R. (2013). Allosteric integrase inhibitor potency is determined through the inhibition of HIV-1 particle maturation. Proc. Natl. Acad. Sci. USA.

[61] Wu X., Liu H., Xiao H., Conway J.A., Hehl E., Kalpana G.V., Prasad V., Kappes J.C. (1999). Human immunodeficiency virus type 1 integrase protein promotes reverse transcription through specific interactions with the nucleoprotein reverse transcription complex. J. Virol..

[62] Hehl E.A., Joshi P., Kalpana G.V., Prasad V.R. (2004). Interaction between human immunodeficiency virus type 1 reverse transcriptase and integrase proteins. J. Virol..

[63] Tekeste S.S., Wilkinson T.A., Weiner E.M., Xu X., Miller J.T., Le Grice S.F.J., Clubb R.T., Chow S.A. (2015). Interaction between Reverse Transcriptase and Integrase Is Required for Reverse Transcription during HIV-1 Replication. J. Virol..

[64] Shi J., Zhou J., Shah V.B., Aiken C., Whitby K. (2011). Small-molecule inhibition of human immunodeficiency virus type 1 infection by virus capsid destabilization. J. Virol..

[65] Gupta M., Pak A.J., Voth G.A. (2023). Critical mechanistic features of HIV-1 viral capsid assembly. Sci. Adv..

[66] Sowd G.A., Serrao E., Wang H., Wang W., Fadel H.J., Poeschla E.M., Engelman A.N. (2016). A critical role for alternative polyadenylation factor CPSF6 in targeting HIV-1 integration to transcriptionally active chromatin. Proc. Natl. Acad. Sci. USA.

[67] de Chaumont F., Dallongeville S., Chenouard N., Hervé N., Pop S., Provoost T., Meas-Yedid V., Pankajakshan P., Lecomte T., Le Montagner Y. (2012). Icy: an open bioimage informatics platform for extended reproducible research. Nat. Methods.

[68] Pizzato M., Erlwein O., Bonsall D., Kaye S., Muir D., McClure M.O. (2009). A one-step SYBR Green I-based product-enhanced reverse transcriptase assay for the quantitation of retroviruses in cell culture supernatants. J. Virol. Methods.

[69] Jang S., Bedwell G.J., Singh S.P., Yu H.J., Arnarson B., Singh P.K., Radhakrishnan R., Douglas A.W., Ingram Z.M., Freniere C. (2024). HIV-1 usurps mixed-charge domain-dependent CPSF6 phase separation for higher-order capsid binding, nuclear entry and viral DNA integration. Nucleic Acids Res..

[70] Sanders-Beer B.E., Eschricht M., Seifried J., Hirsch V.M., Allan J.S., Norley S. (2012). Characterization of a monoclonal anti-capsid antibody that cross-reacts with three major primate lentivirus lineages. Virology.

[71] Varthakavi V., Browning P.J., Spearman P. (1999). Human immunodeficiency virus replication in a primary effusion lymphoma cell line stimulates lytic-phase replication of Kaposi's sarcoma-associated herpesvirus. J. Virol..

[72] Peck A., Yu Y., Paraan M., Kimanius D., Ermel U.H., Hutchings J., Serwas D., Siems H., Hill N.S., Ali M. (2025). AreTomoLive: Automated reconstruction of comprehensively-corrected and denoised cryo-electron tomograms in real-time and at high throughput. bioRxiv.

