# Supplementary material for: Capsid and integrase play essential apposing roles in viral ribonucleoprotein assembly during HIV-1 core morphogenesis
Source: iScience. 2026 Jun 22;29(7):116378. doi: 10.1016/j.isci.2026.116378 (PMC13316281; doi:10.1016/j.isci.2026.116378)
Supplement: Document S1. Figures S1–S10 [file mmc1.pdf]

iScience, Volume *29*

Supplemental information

Capsid and integrase play essential apposing roles in viral ribonucleoprotein assembly during HIV-1 core morphogenesis

Ahinsa Ranaweera, Jonathan R. Andino-Moncada, Sarah E. Dillon, Satya P. Singh, Scott M. Stagg, Alan N. Engelman, Christopher Aiken, and Ashwanth C. Francis

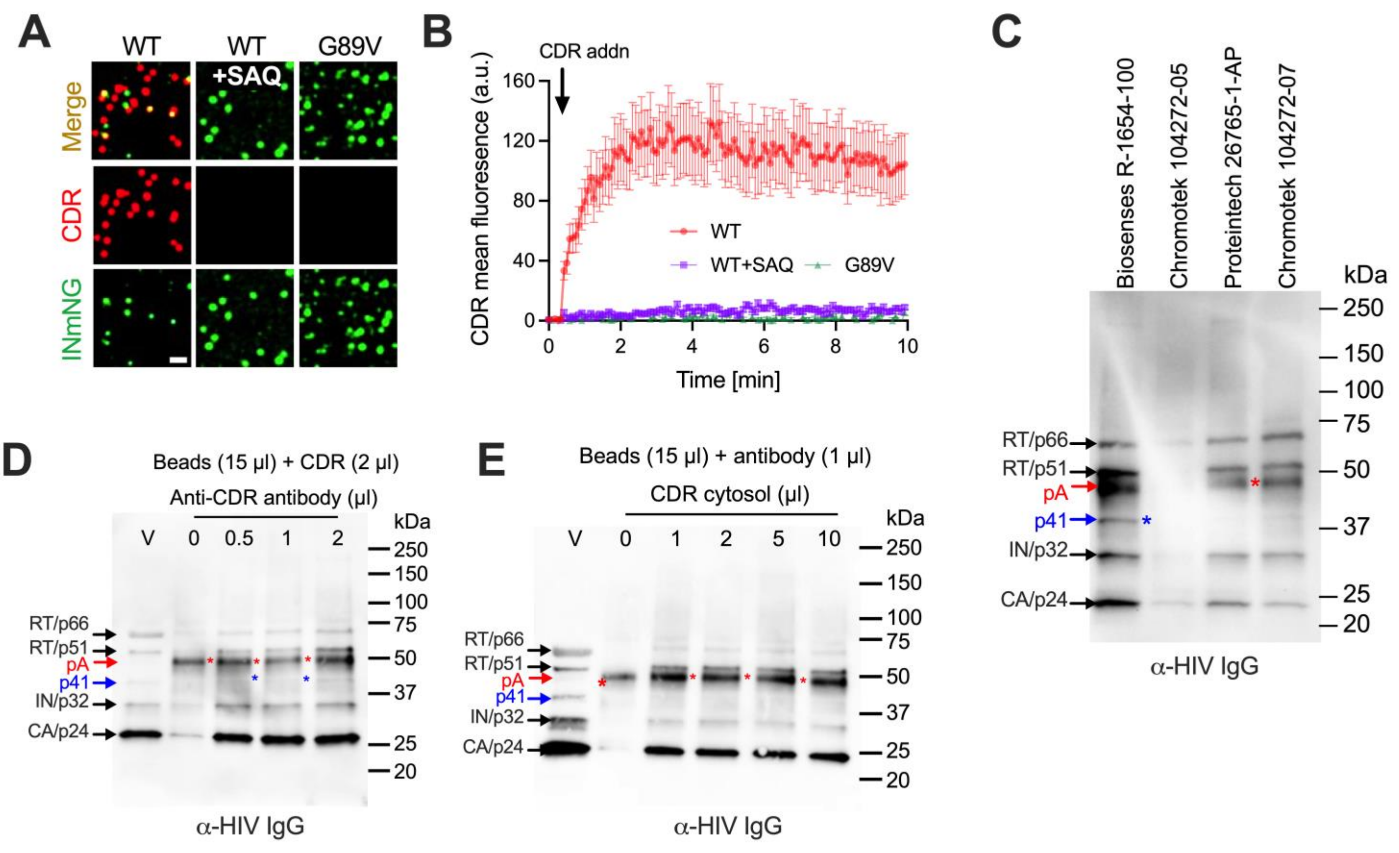

Fig. S1. CDR binding and affinity capture of mature HIV-1 cores. (A) Images show that CDR (red) co-localized with saponin-permeabilized WT viruses labeled with INmNG (green) but not the G89V CA mutant or immature viruses produced in the presence of saquinavir (+SAQ). **(B)** Fluorescence intensity traces show the binding of 50 nM CDR to WT cores (DMSO) reaching a plateau by 2 min after CDR addition, and poor binding of CDR to G89V/CA and SAQ-treated cores. Data in (B) averaged intensities of >50-single binding events, and error bars are SEM. **(C)** Immunoblots showing capture of HIV-1 cores using different mono- and polyclonal antibodies; low recovery in lane 2 indicates rat-anti-RFP chromotek antibody does not bind CDR-protein efficiently. **(D, E)** Titration experiments showing efficient capture with as little as 0.5 µl (1 µg/µl) of antibody (D) or 1 µl (0.1 µg) of CDR in cytosol extracts (E). Note the absence of captured cores and the presence of ~50 kDa protein A (pA) from beads in ‘0 µl’ control lanes in (D and E). Putative viral proteins (*black*), pA (*red arrows, and stars*), and ~p41 kDa Gag-processing intermediate (*blue arrows and stars*) are overlaid for clarity.

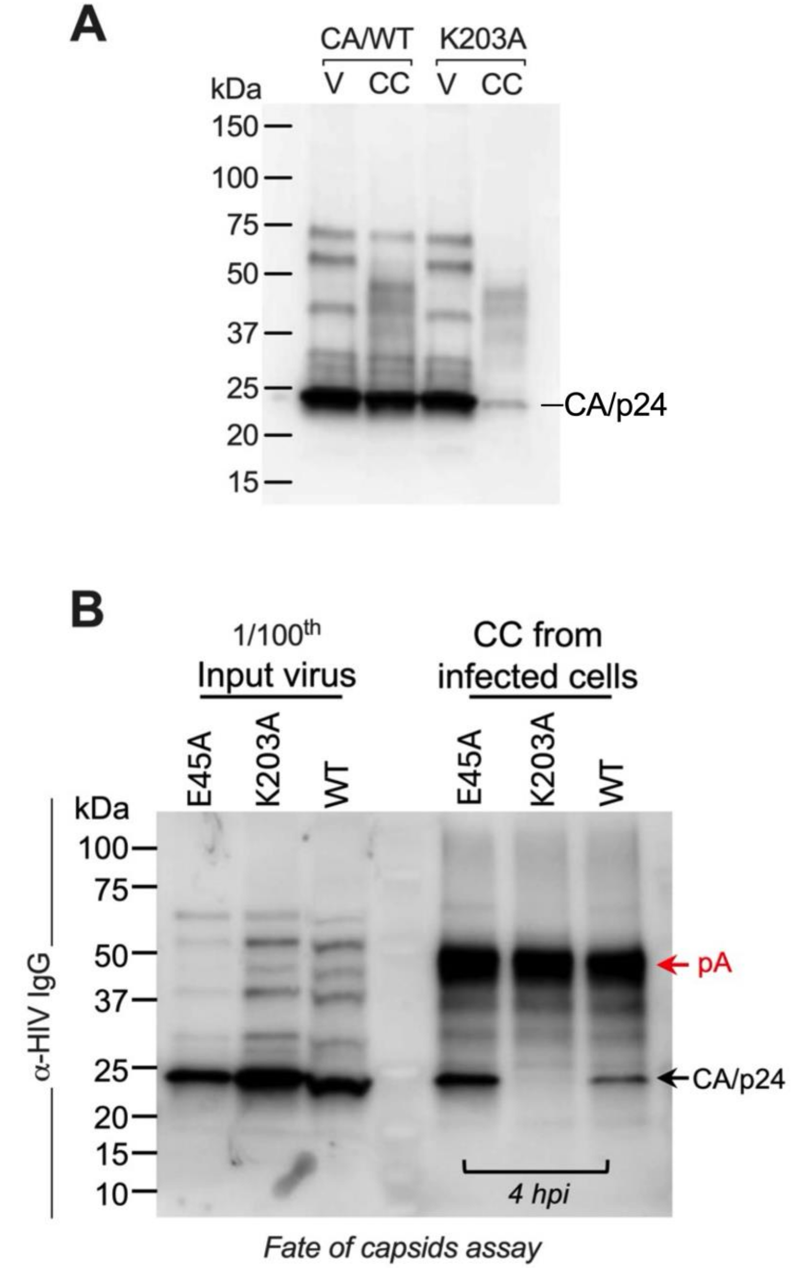

Fig. S2. Affinity capture enriches stable, assembled HIV-1 cores from membrane permeabilized virions and infected cells. (A) An additional example (related to Fig. 1D-E) with parallel loading of WT and K203A input and CC products. **(B)** A representative immunoblot (n=3 experiments) from 'fate-of-capsid' experiments. HEK293T cells were infected for 4 h (MOI-40) with VSV-G-pseudotyped HIV-1 containing the indicated WT or mutant capsids, and cellular lysates were used for affinity capture of cores. Immunoblots of captured cores were probed with anti-HIV serum and show the capture of stable capsids (WT and E45A), but not unstable K203A cores.

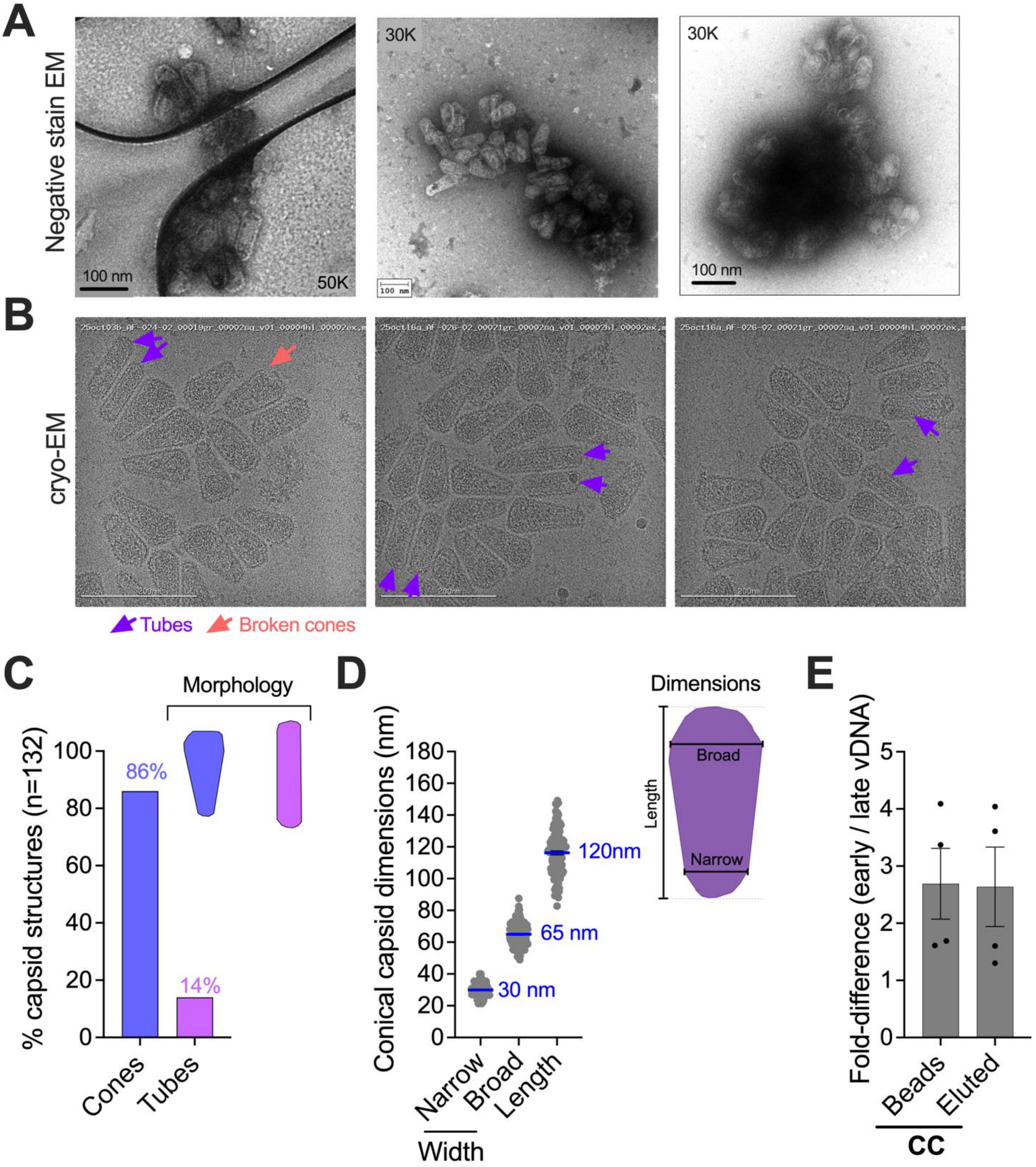

Fig. S3. Affinity capture purifies authentic HIV-1 capsids. (A) Negative stain EM and **(B)** cryo-EM micrographs of CsA-eluted cores. Example negative stain EM images, collected at 50,000x (left) and 30,000x magnification, show clustered conical shaped HIV-1 capsids. Example high-magnification cryo-EM images, collected at 37,000x magnification, show well-separated capsids structures. Arrows in (B) point to tubes (purple) and presumably broken conical capsids (pink). Scale bars are 100 nm (A) and 200 nm (B). **(C)** Morphological classification of CsA-eluted cores and a cartoon representation of conical and tubular structures detected in cryo-EM datasets collected from 2 independent experiment. **(D)** Measurements of indicated dimensions of isolated CsA-eluted cores (n=103) as shown in the accompanying cartoon. **(E)** Fold-difference of early and late ERT products from 4 independent experiments is shown.

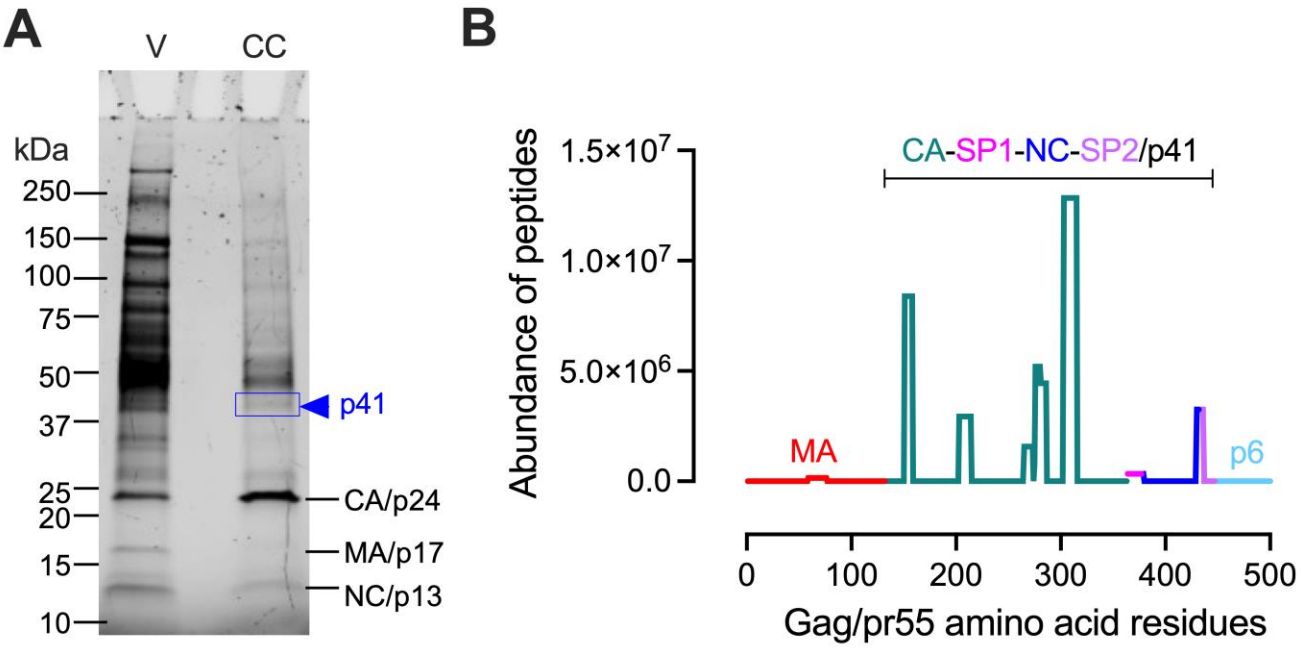

Fig. S4. Mass spectrometry analysis of p41 kDa Gag processing intermediate. (A) Stain free gel image of input virions (V) and CsA eluted cores (CC) with identification of putative viral proteins with expected molecular weight. Blue boxed region shows the ~41 kDa band that was trypsinized and used for mass spectrometry studies. **(B)** Mass-spectrometry peptide enrichment analysis showing the abundance of peptide segments corresponding to Gag/Pr55 precursor protein from 2 independent experiment. Data shows enrichment of central CA residues, SP1, and NC/SP2 cleavage sites, with poor enrichment of the MA domain .

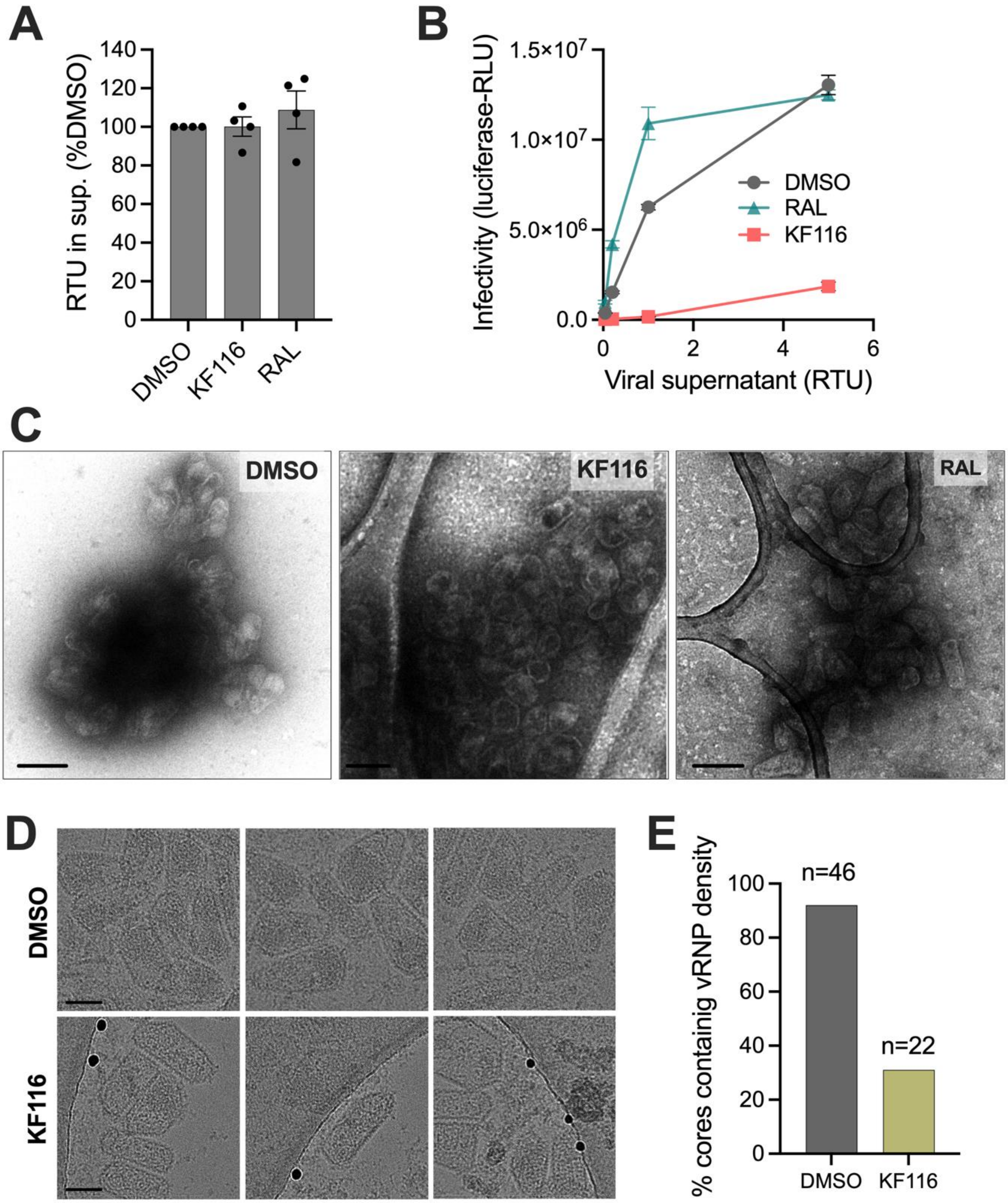

Fig. S5. Effects of ALLINI KF116 on HIV-1 particle release, infectivity, and capsid structure. (A) SG-PERT RT quantitation of viral supernatants and **(B)** normalized infectivity of virions produced in the presence of KF116 (1 µM), DMSO, or RAL (10 µM). Data is mean and standard error from 4 independent virus production (A) and 3 independent infectivity experiments (B). **(C)** Negative stain EM images of CsA-eluted cores from DMSO, KF116, or RAL-treated virions show clusters of canonical capsid structures. Scale bars are 100 nm. **(D)** Examples of cryo-EM images of cores produced in the presence of DMSO (top panels), and KF116 (bottom panels). All images are of same dimension, and scale bar (50 nm) is overlaid on left most panels. **(E)** Classification of cryo-EM structures with vRNP densities. The number of cores analyzed (n) from a single experiment is overlaid for clarity.

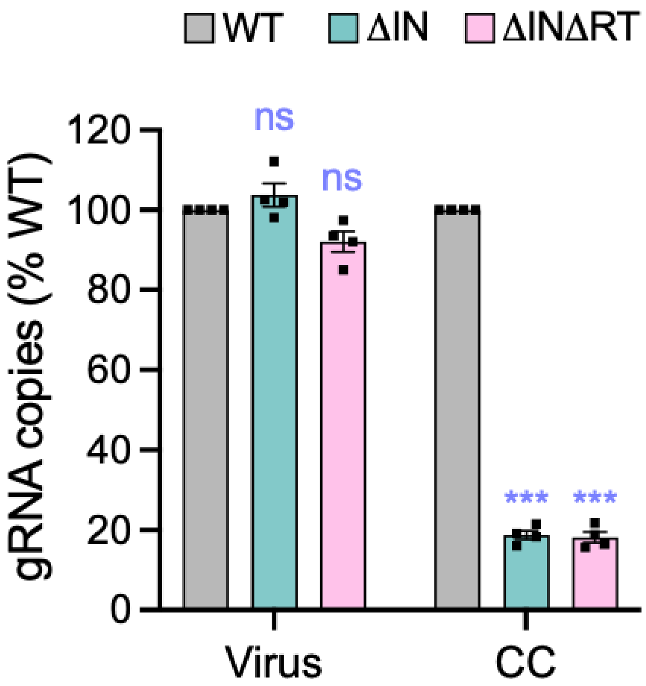

Fig. S6. HIV-1 gRNA encapsidation in IN- and RT deleted virions. Q-PCR analysis of gRNA in full-length (WT), or IN deleted (ΔIN), or RT and IN deleted (ΔINΔRT) virions and captured cores. gRNA copies were normalized to WT from 4 independent experiments. Statistical significance *(vs.* WT*)*, $p<0.001$ (***) and $p>0.05$ not significant (ns), was determined by Student's t-test. Data is related to main Fig. 4.

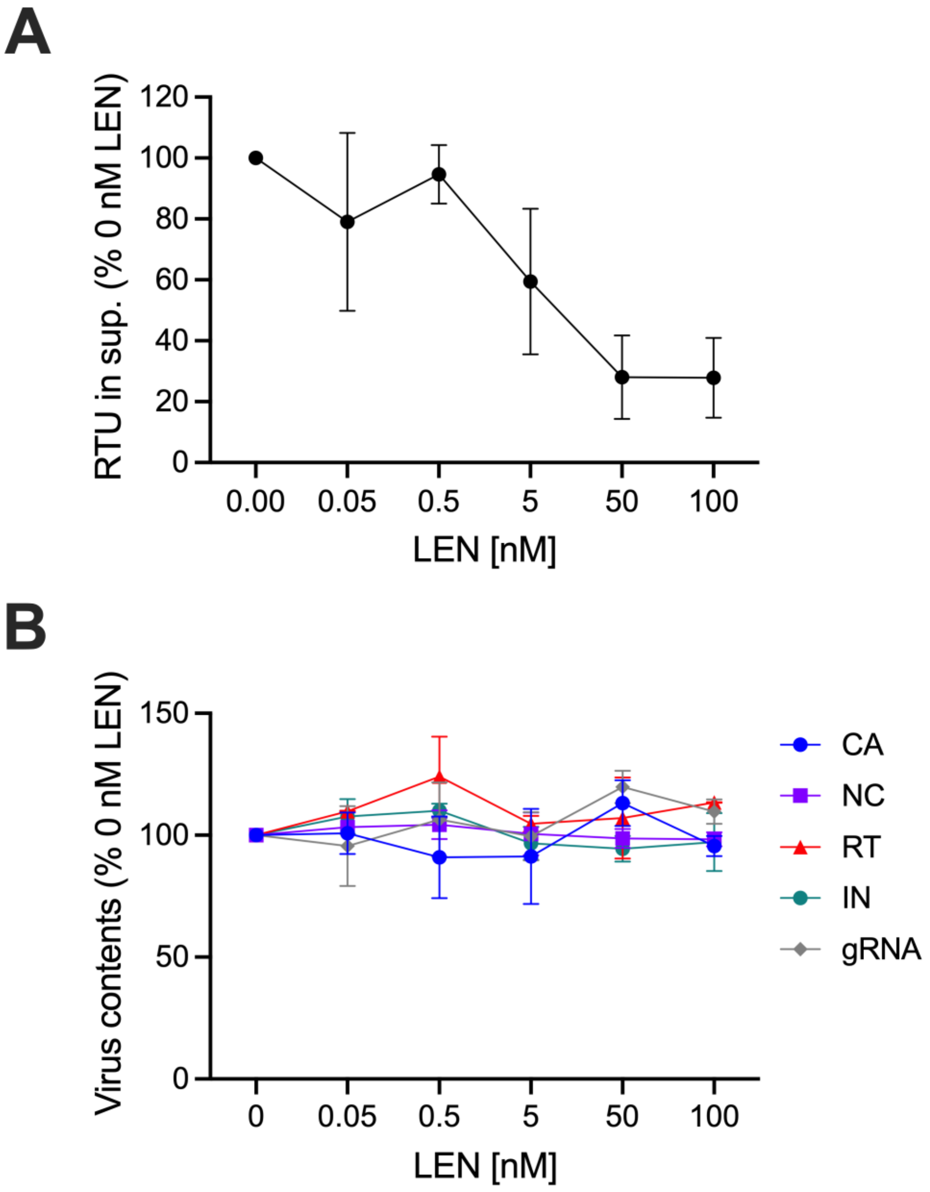

Fig. S7 – LEN impairs HIV-1 particle release but does not affect content packaging into virions. (A) SG-PERT RT quantitation of viral supernatants produced in the presence of indicated concentrations of LEN. **(B)** Densitometry analysis of content incorporation into virus (related to Fig. 5B, C), illustrates that LEN does not affect the proteolytic processing of CA, NC, RT and IN in virions. Data is mean and standard error from n=4 experiments normalized to DMSO (0 nM) control .

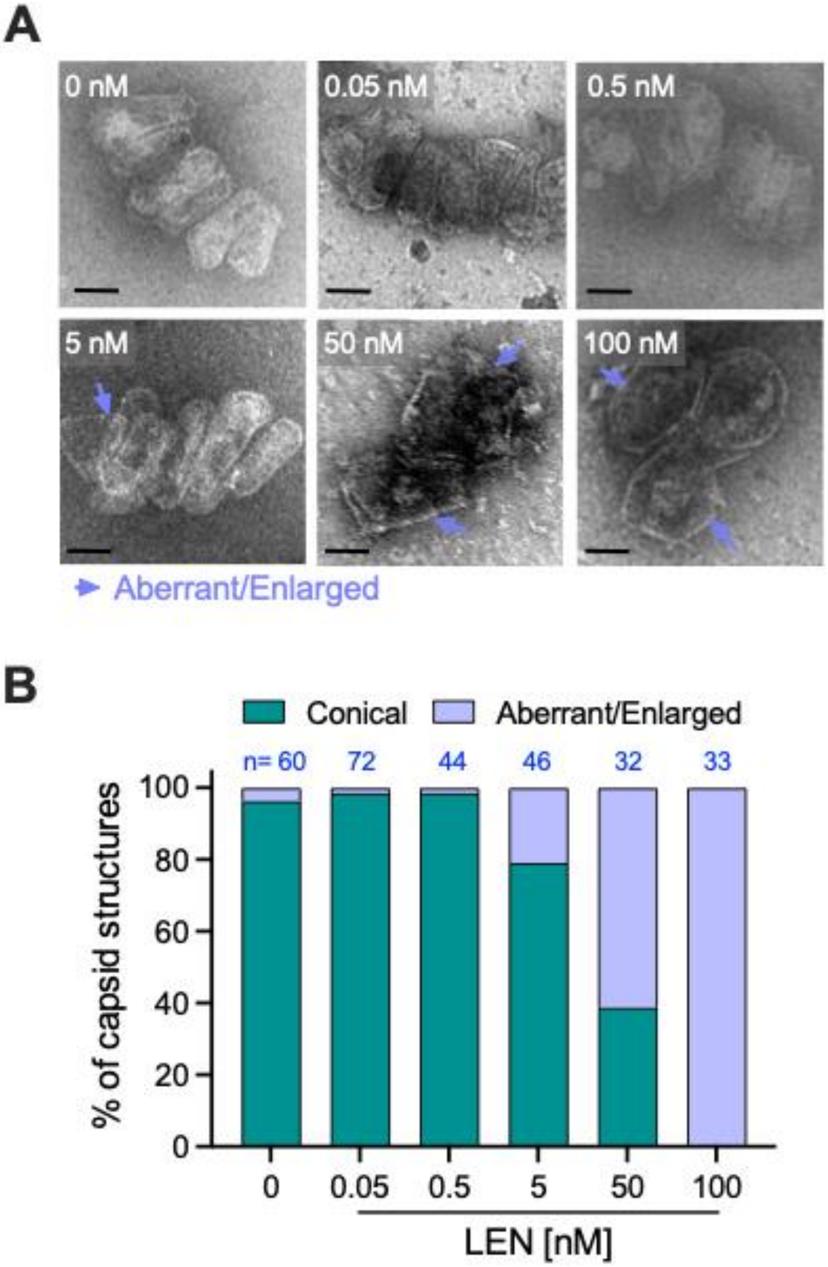

Fig. S8 – LEN reshapes the structure of HIV-1 cores. (A) Negative stain EM images and **(B)** quantification shows a shift from conical appearance to aberrant, flatter- and enlarged (*blue arrow*) capsid morphology. The number of capsid structures imaged in each condition is overlaid on (B) for clarity. Scale bar in (A) is 50 nm .

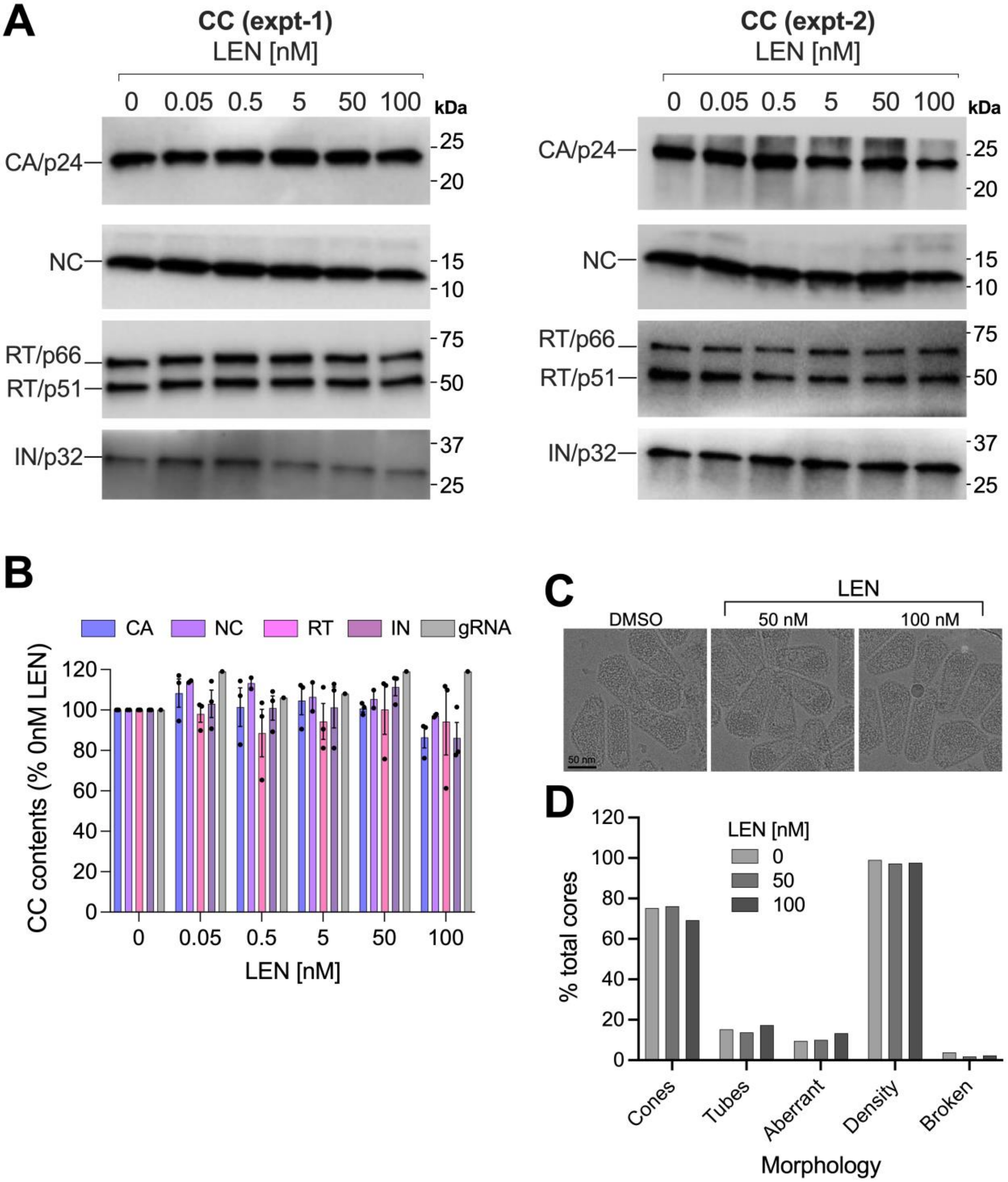

Fig. S9 – Effects of LEN treatment of cores after virus production. HIV-1 virions were treated with indicated concentrations of LEN in the presence of TX100 virus membrane permeabilizing reagent and IP6 (100 μM) for 45 min at room temperature, and the contents (CC) and structure of affinity-captured cores were determined. **(A)** Immunoblots of CC from two independent experiments and **(B)** Densitometry and qPCR-gRNA analysis of CC contents. Note, NC was probed in 2 experiments, gRNA in one, all other proteins were quantified in 3 independent experiments. **(C)** Representative cryo-EM images (scale bar is 50 nm, and image sizes are same across the panel), and **(D)** quantification of the morphology of capsids treated with 0, 50 and 100 nM of LEN from (A)

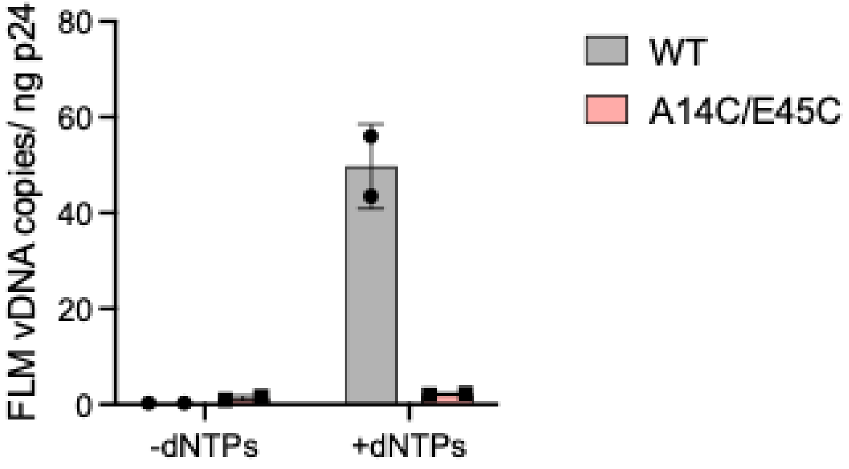

Fig. S10 – Endogenous reverse transcription of full-length vDNA by purified WT and A14C/E45C cores. Sucrose pelleted WT and A14C/E45C cores were incubated with or without dNTPs and FLM vDNA (copies mean ± SD for n=2 experiments) synthesis was assayed by qPCR and is normalized to input (ng p24).
