# Supplementary material for: Capsid and integrase play essential apposing roles in viral ribonucleoprotein assembly during HIV-1 core morphogenesis
Source: iScience. 2026 Jun 22;29(7):116378. doi: 10.1016/j.isci.2026.116378 (PMC13316281; doi:10.1016/j.isci.2026.116378)
Supplement: Data S1. Uncropped western blots of main and supplementary Figures [file mmc3.zip › iScience_DataS1.pdf]

Figure 1C

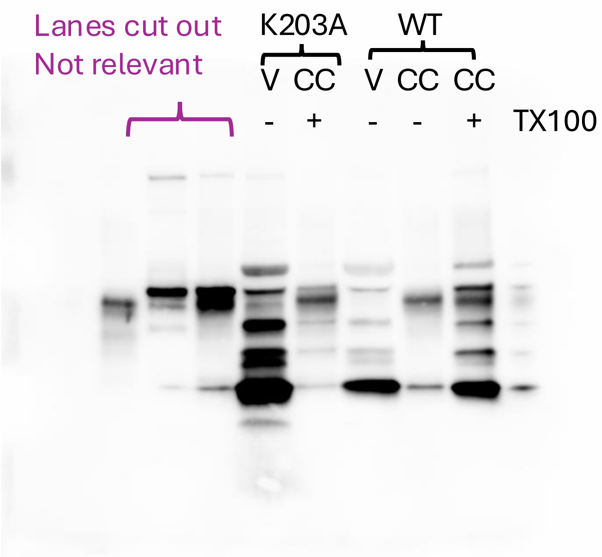

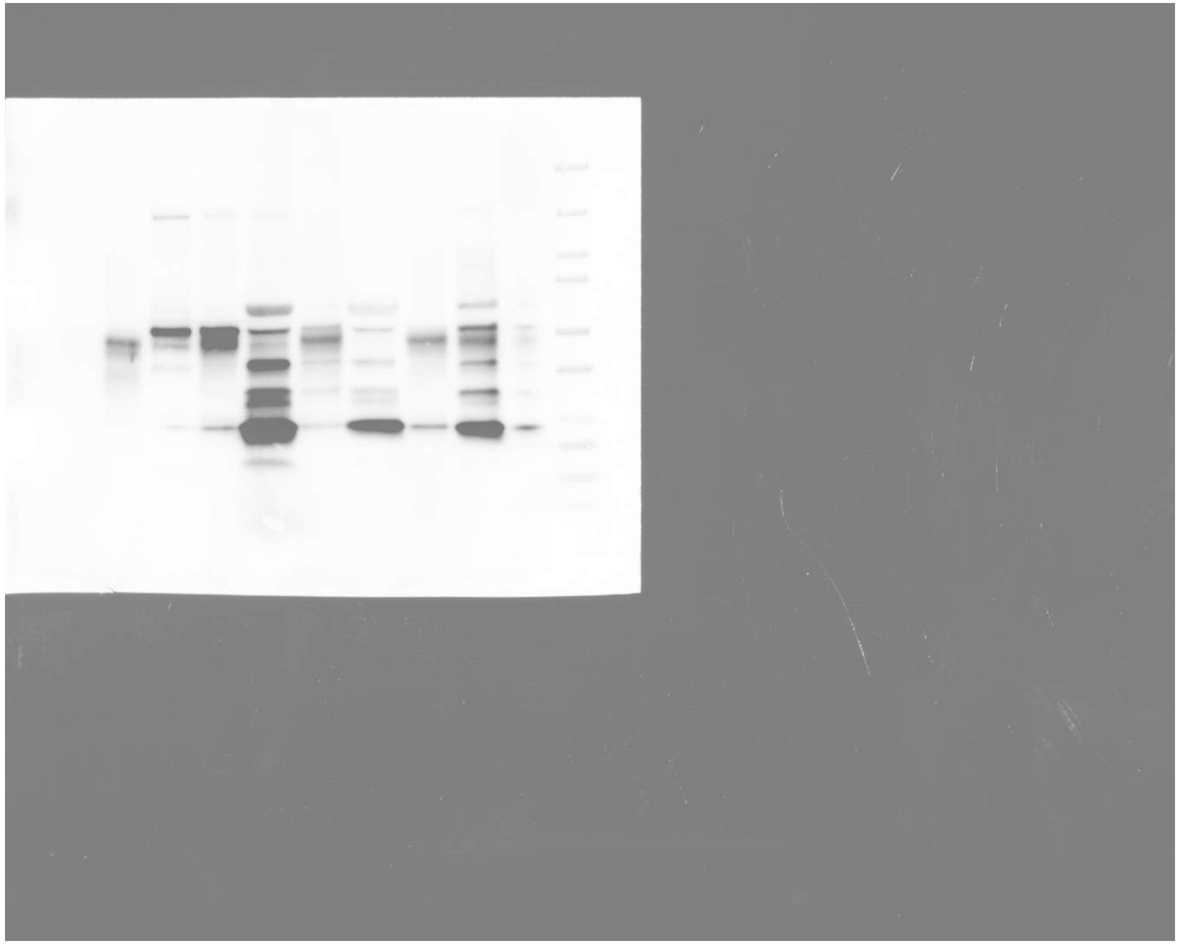

Figure 1E

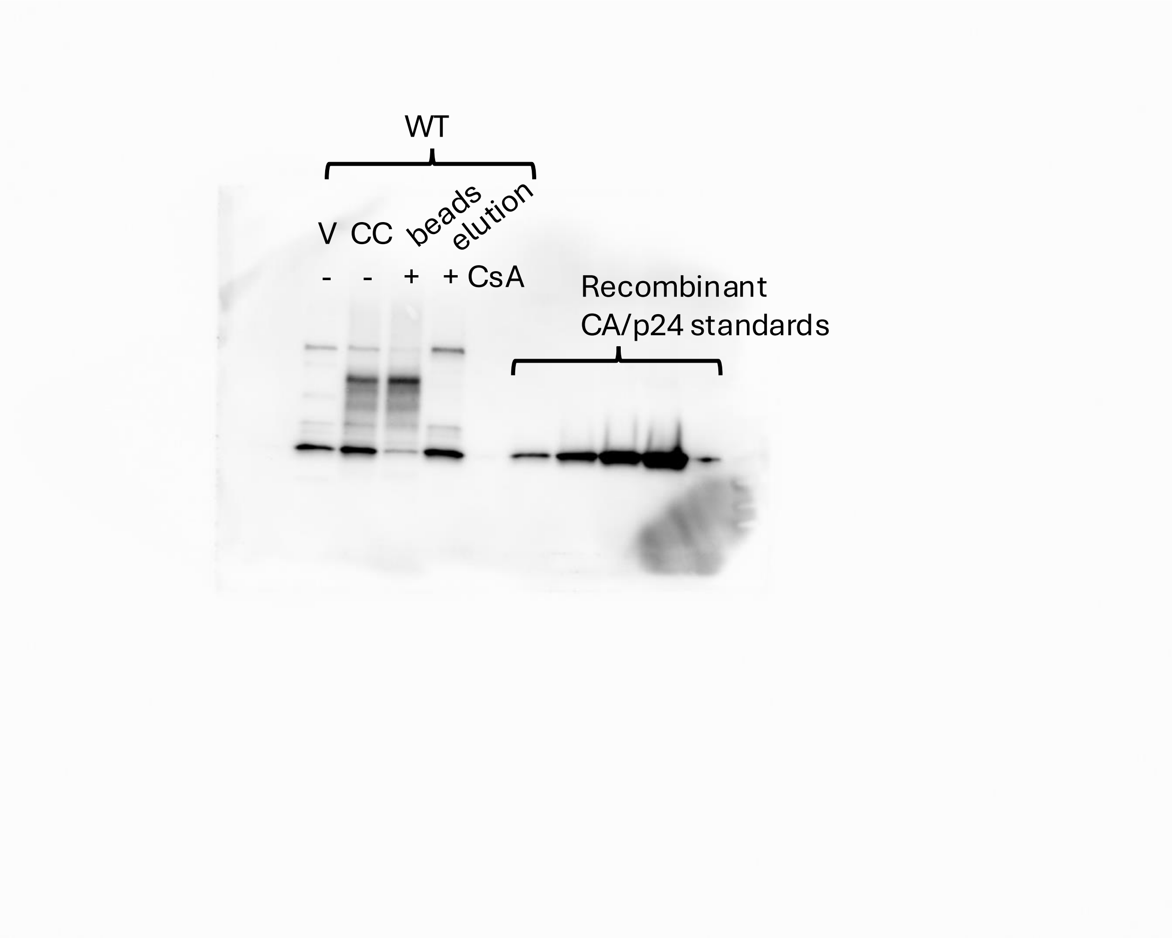

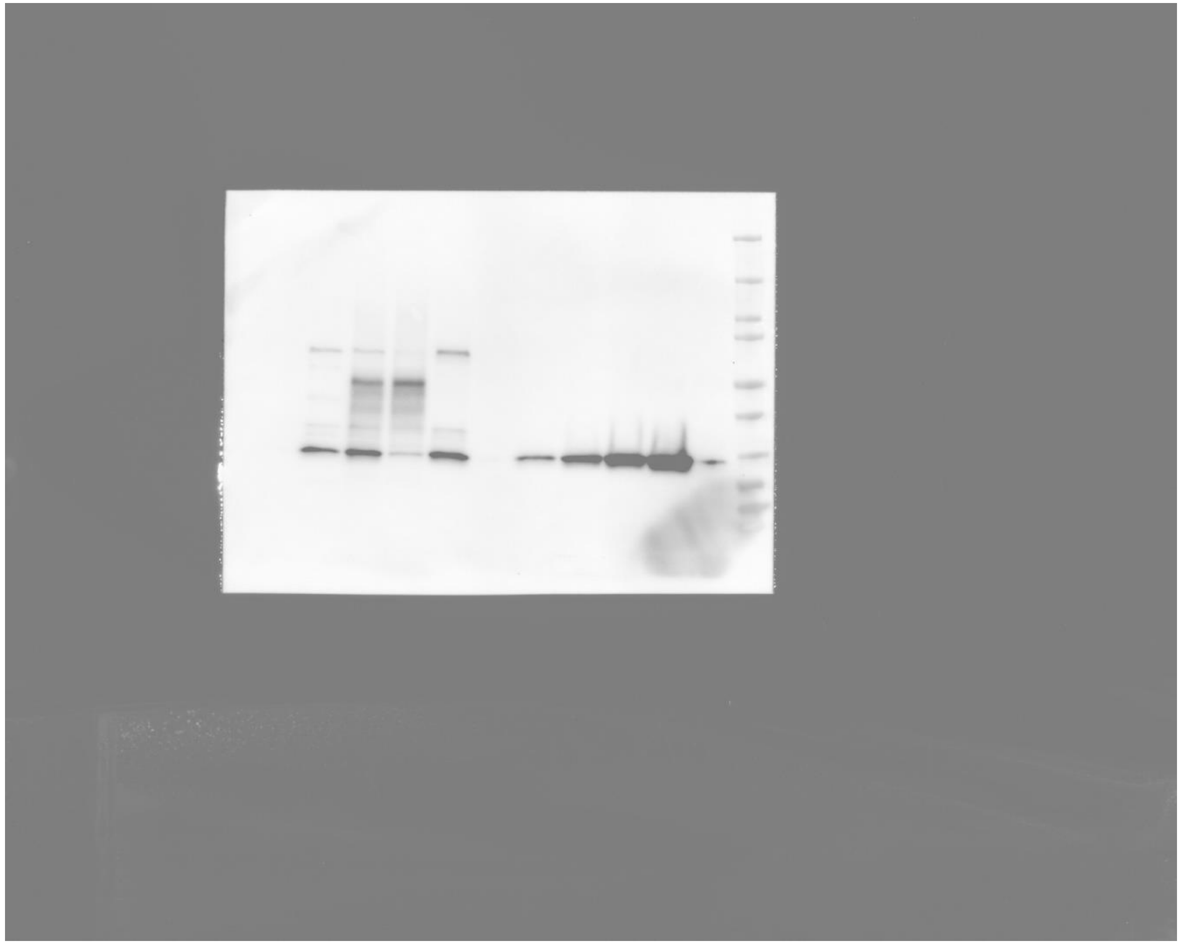

Figure 2A

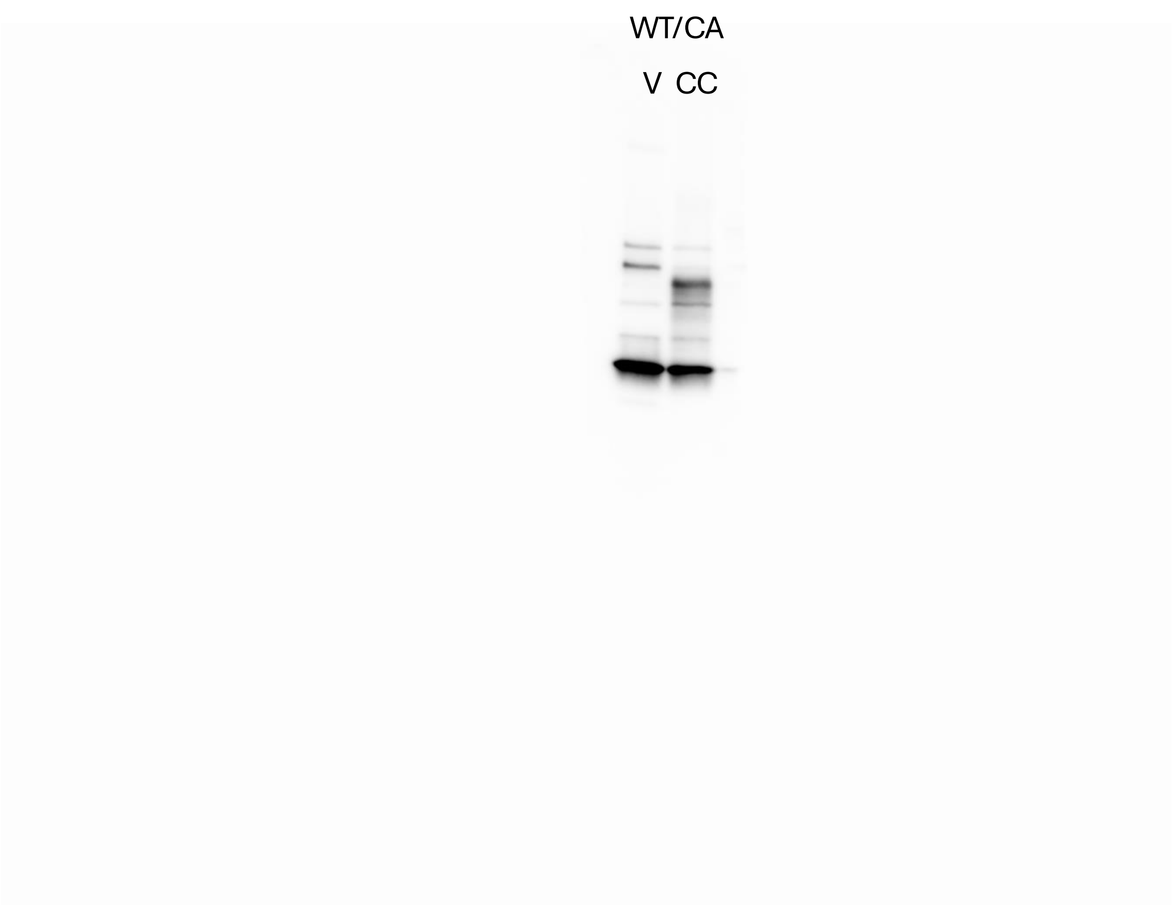

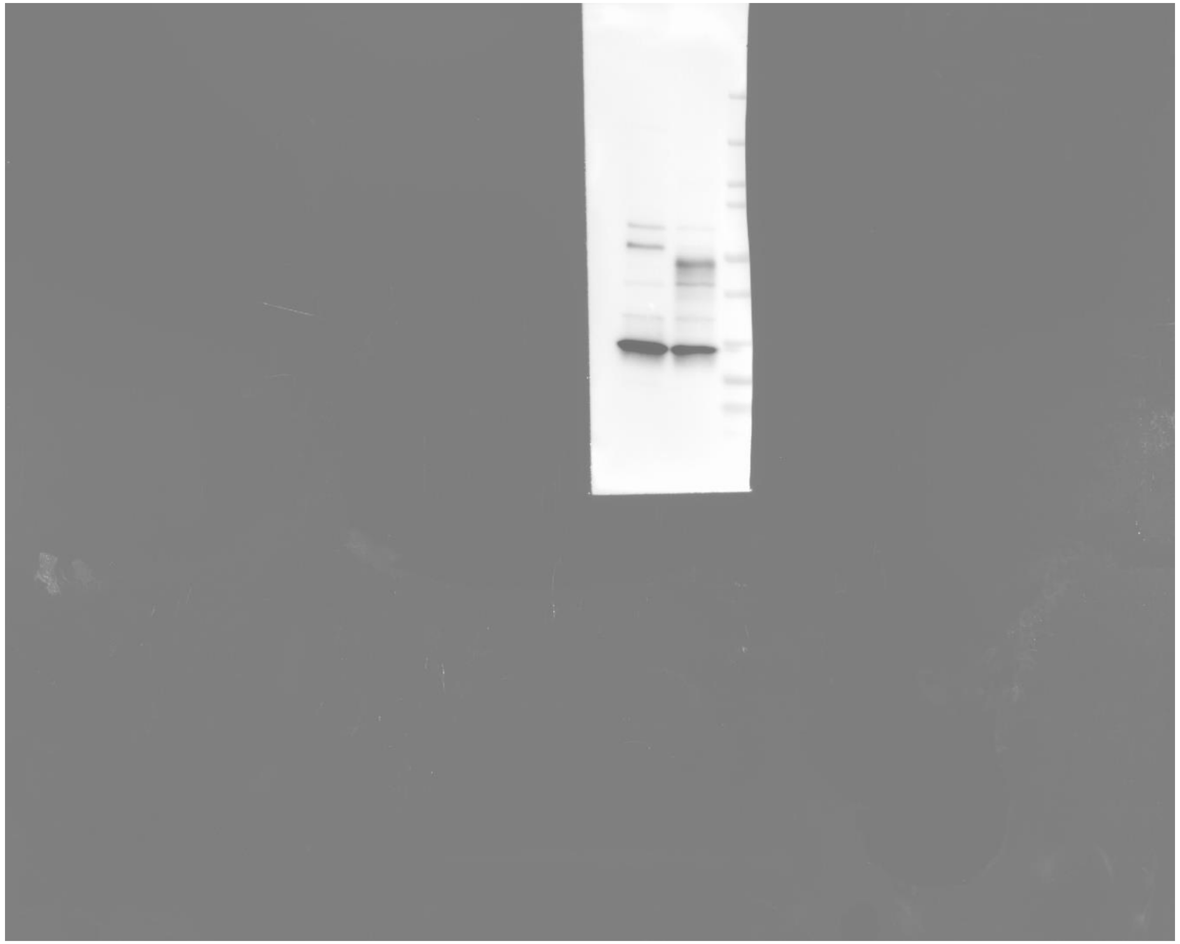

Anti-HIV IgG
Detection of viral proteins

Figure 2B MA

WT/CA

V CC

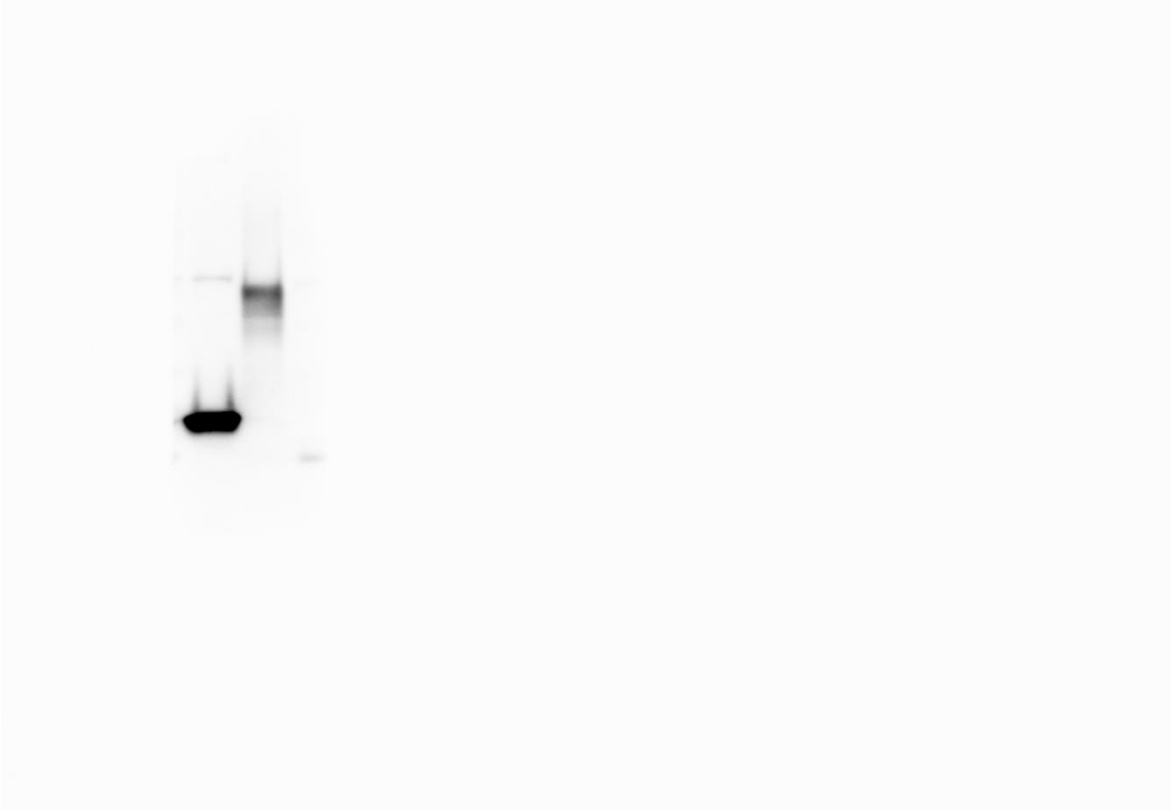

Anti-MA
Detection of MA/p17

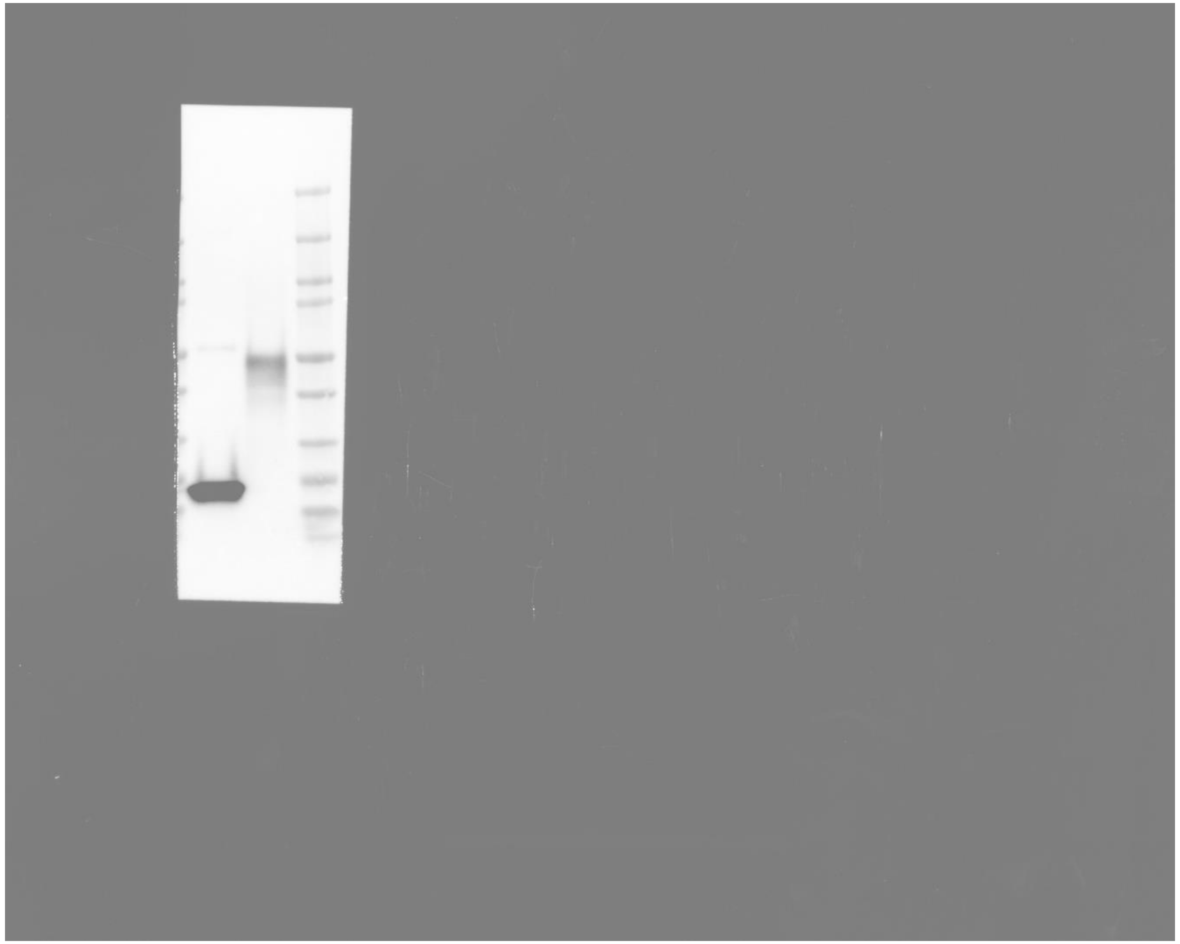

Figure 2B CA

WT/CA

V CC

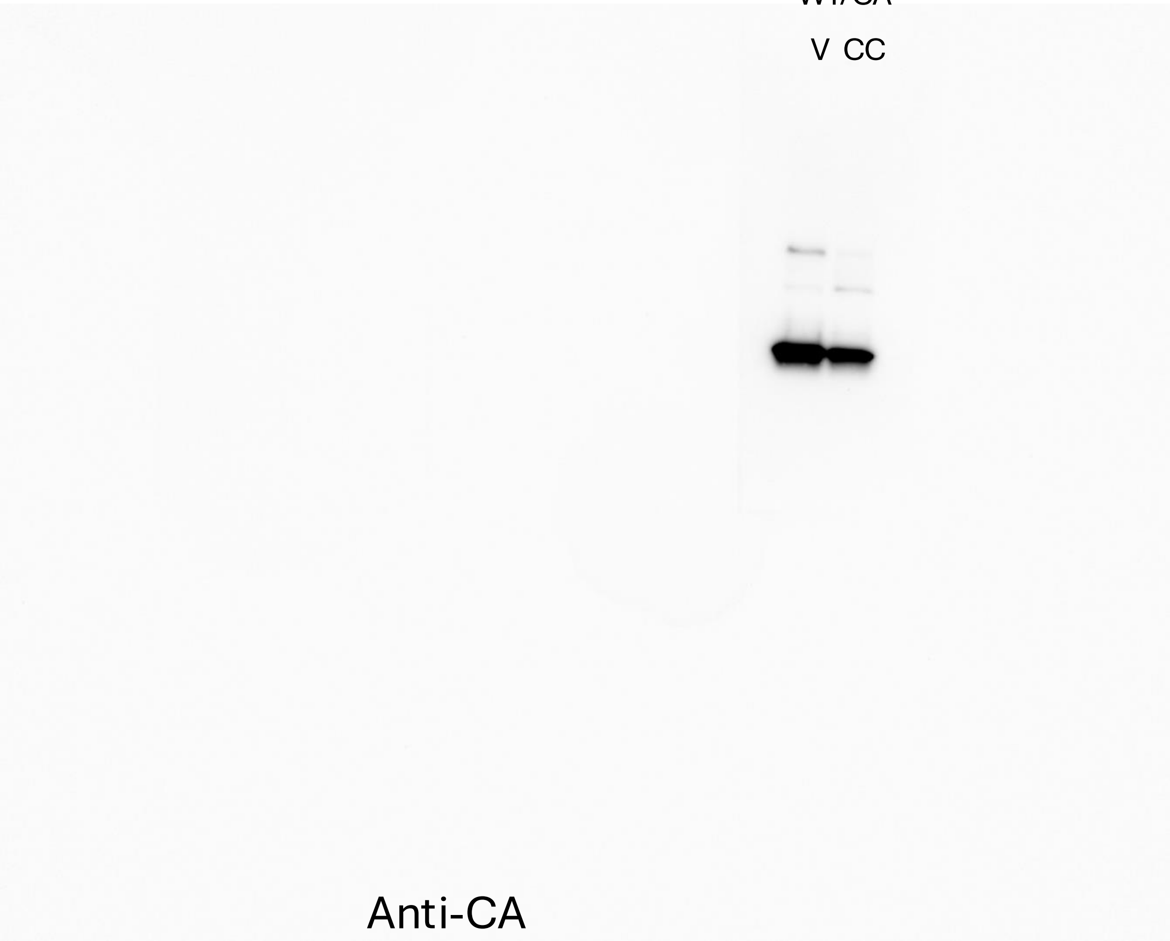

Anti-CA

Detection of CA/p24

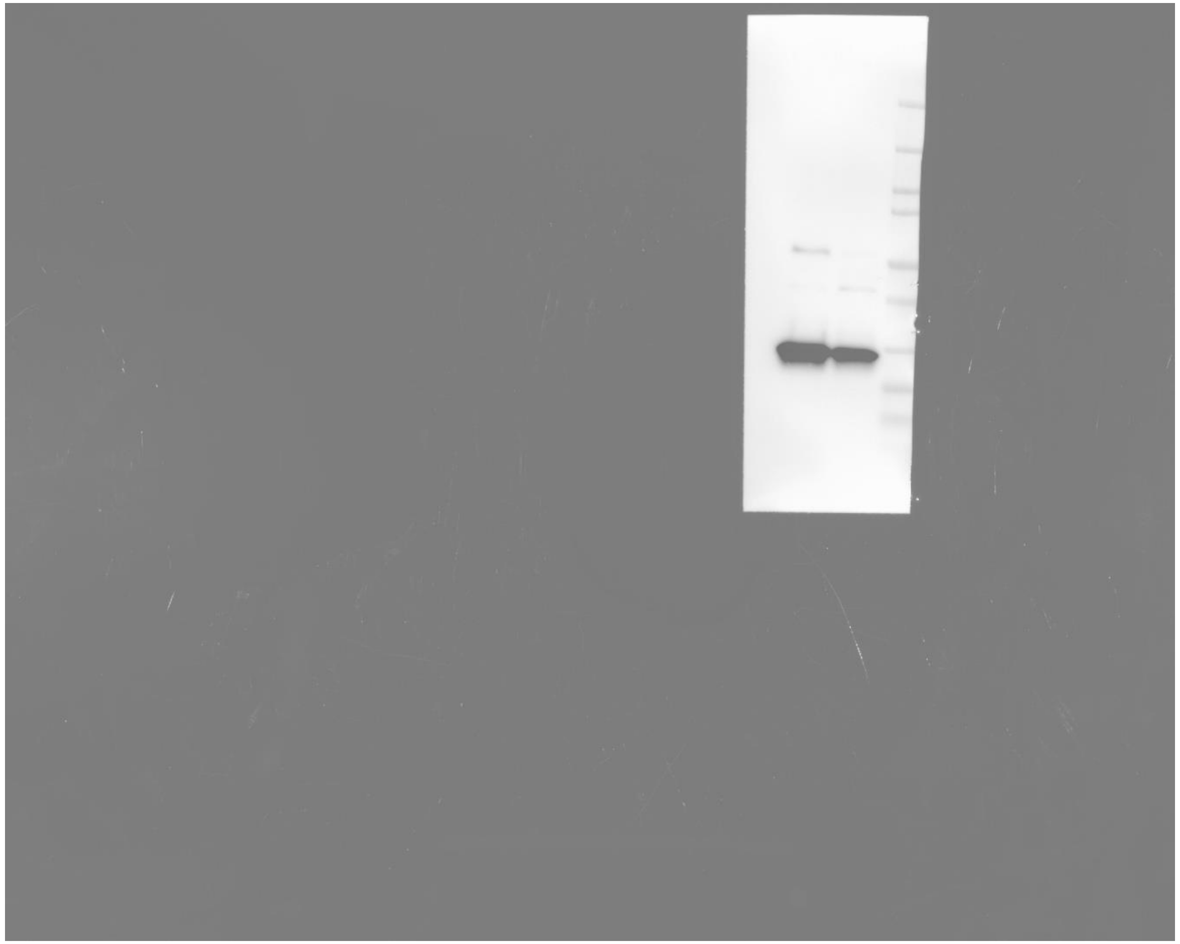

Figure 2B NC

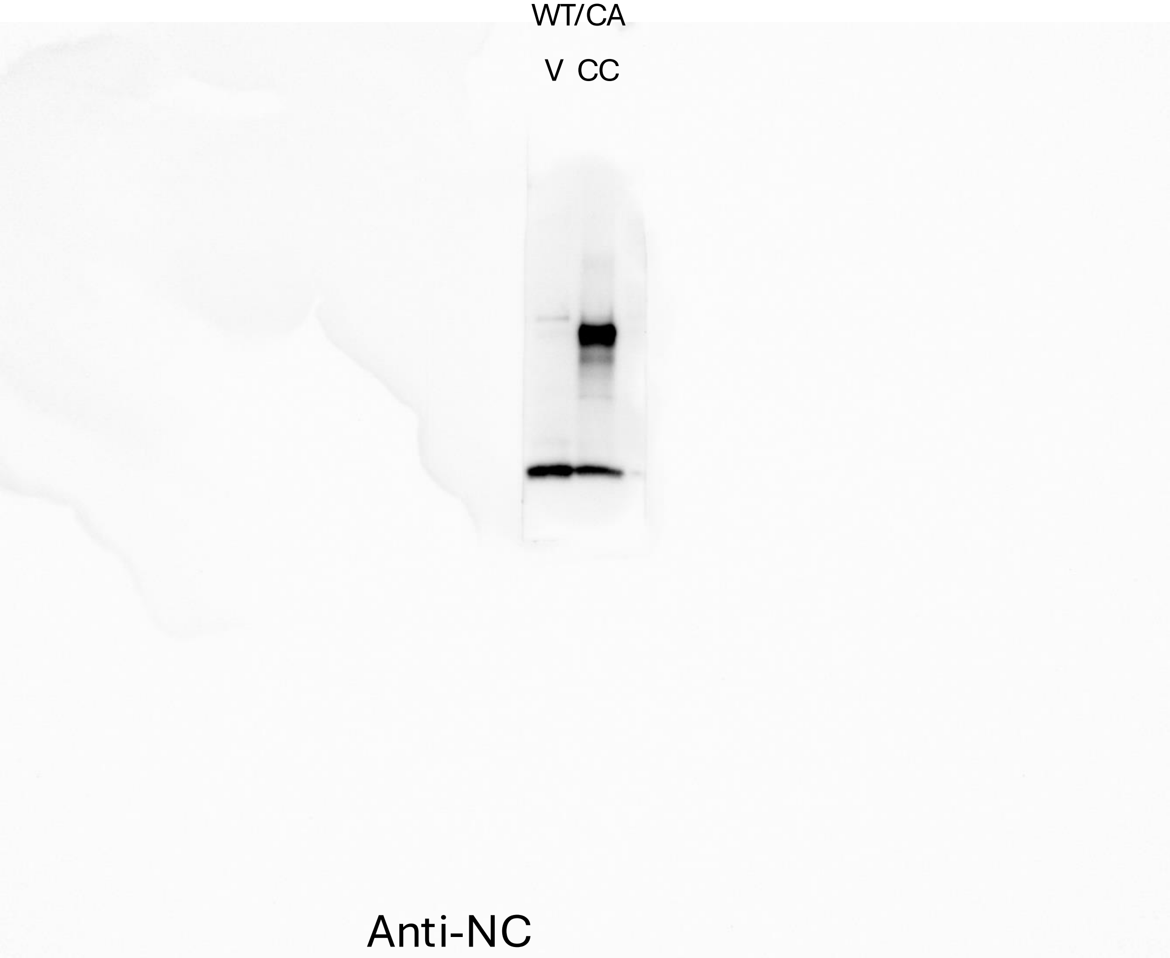

Anti-NC
Detection of NC

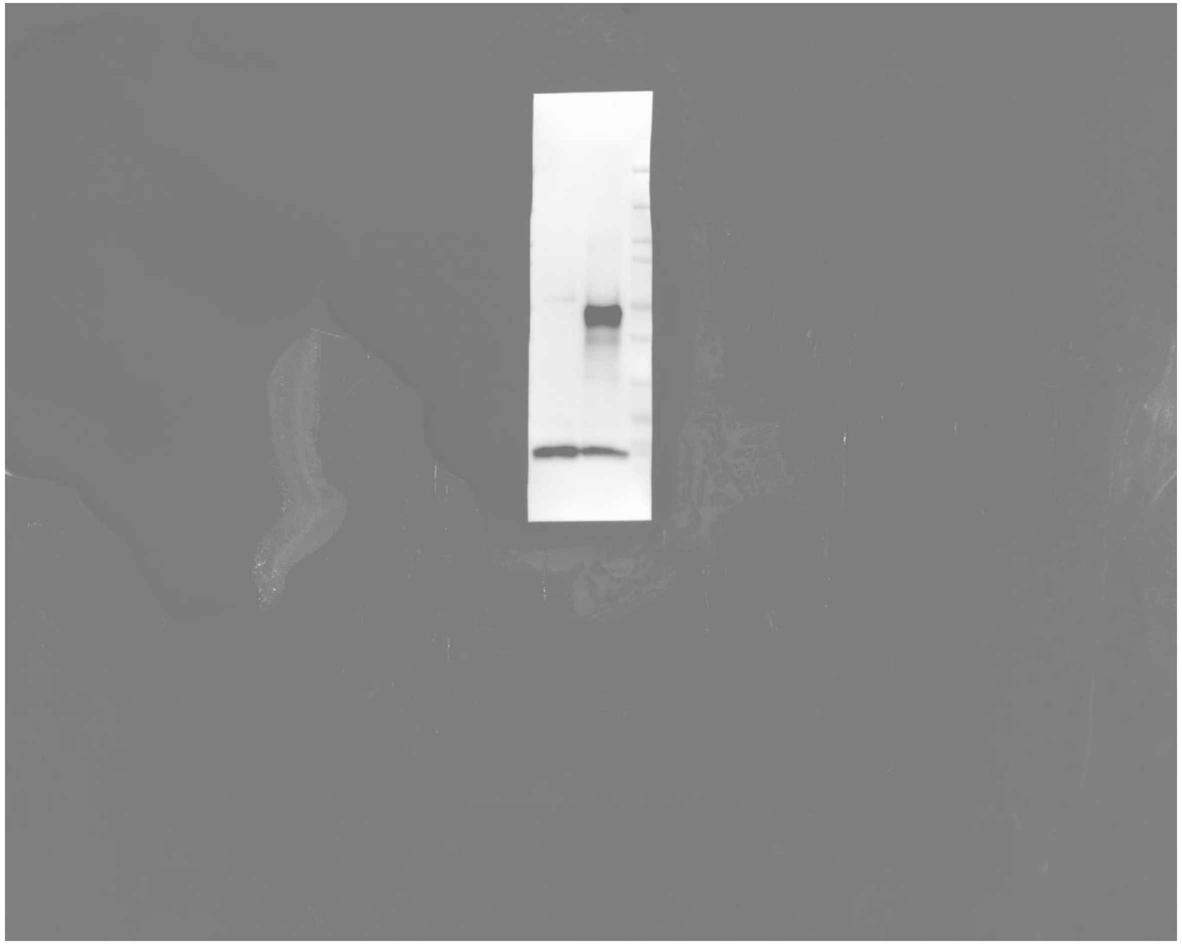

Figure 2C RT

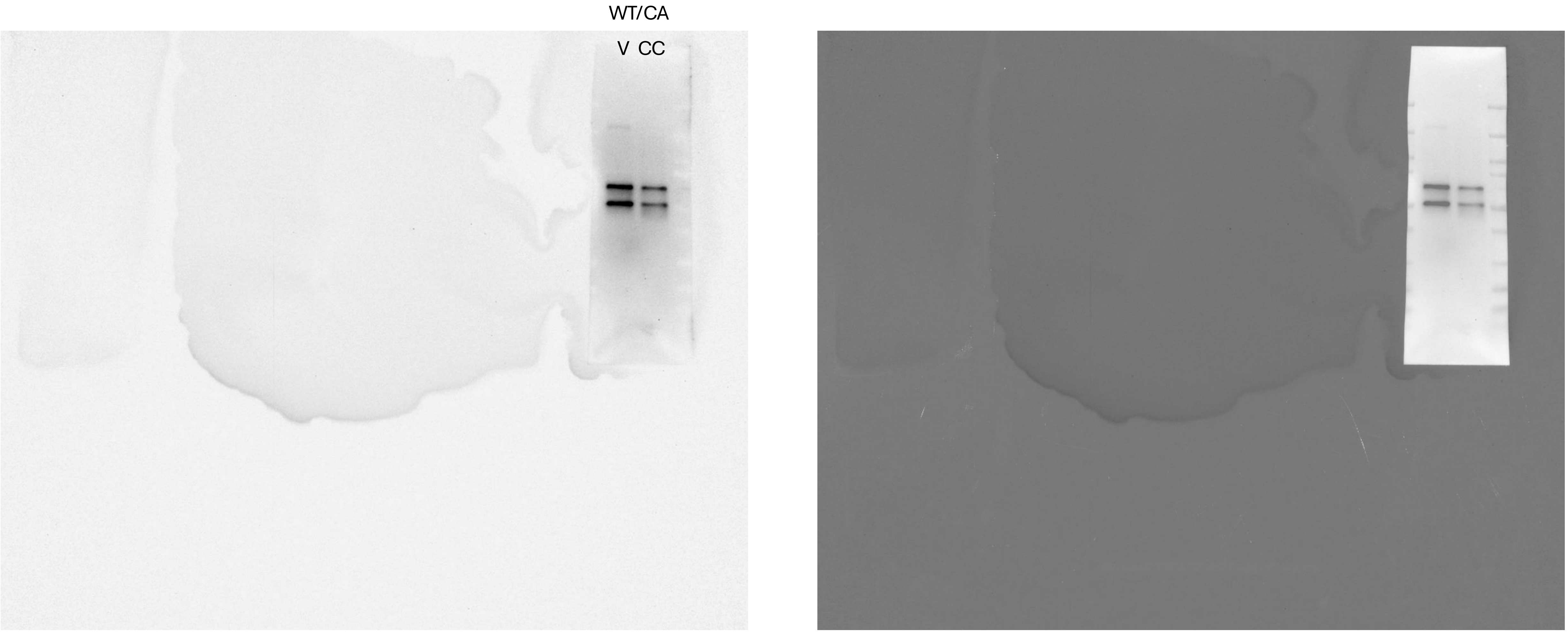

Anti-RT
Detection of RT heterodimers p66/p51

Figure 2C IN

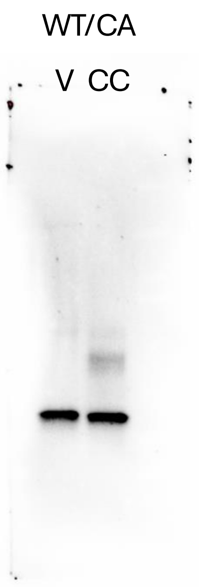

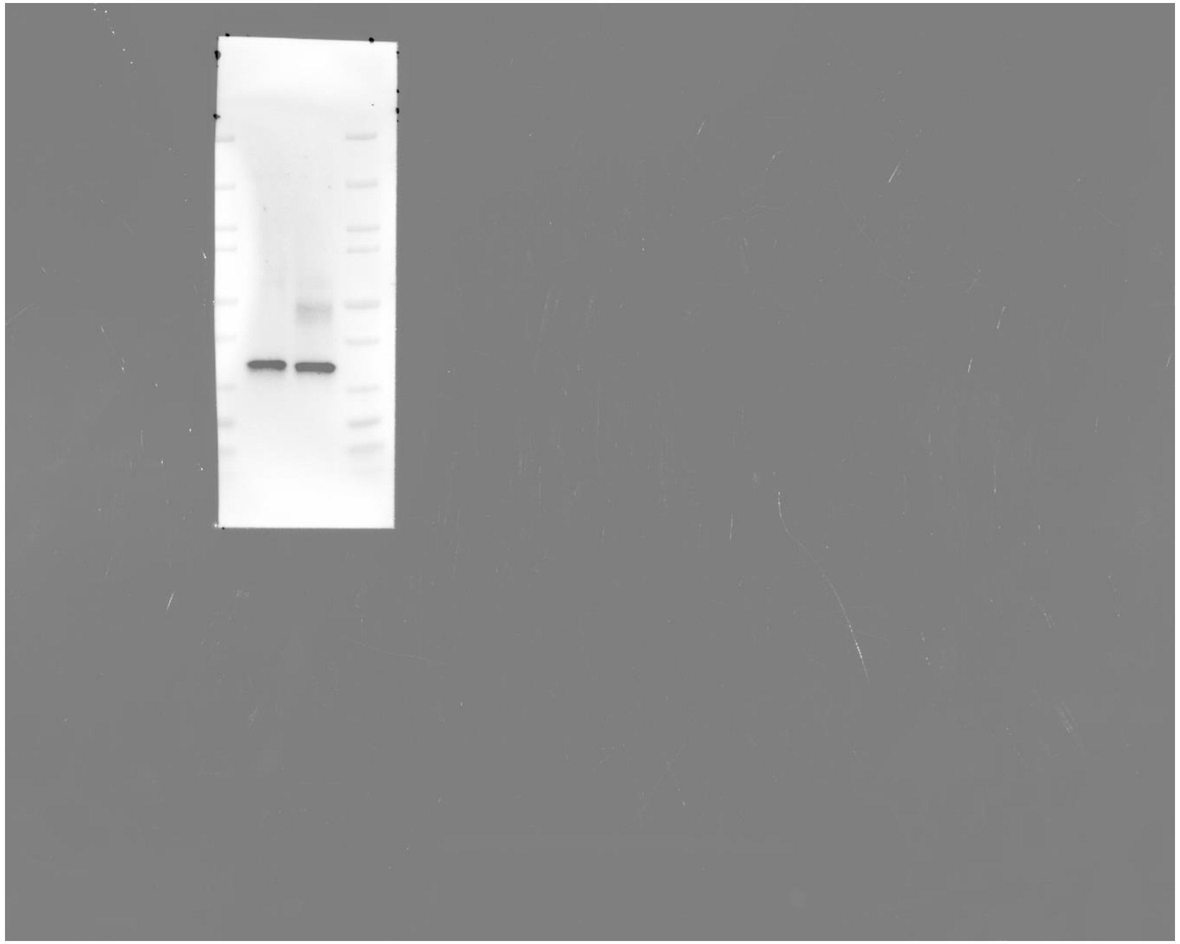

Anti-IN
Detection of IN/p32

Figure 2D

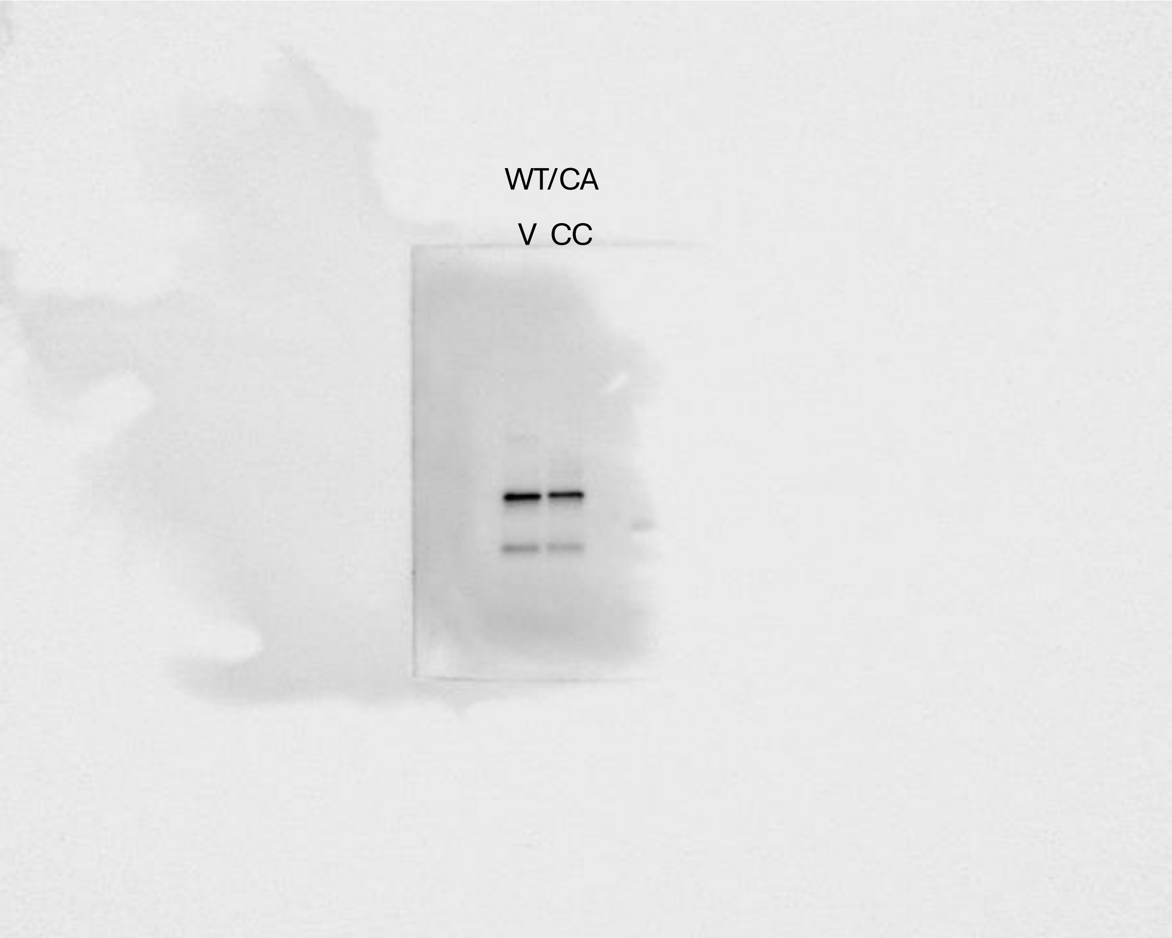

Detection of YFP-Vpr with anti-GFP antibodies

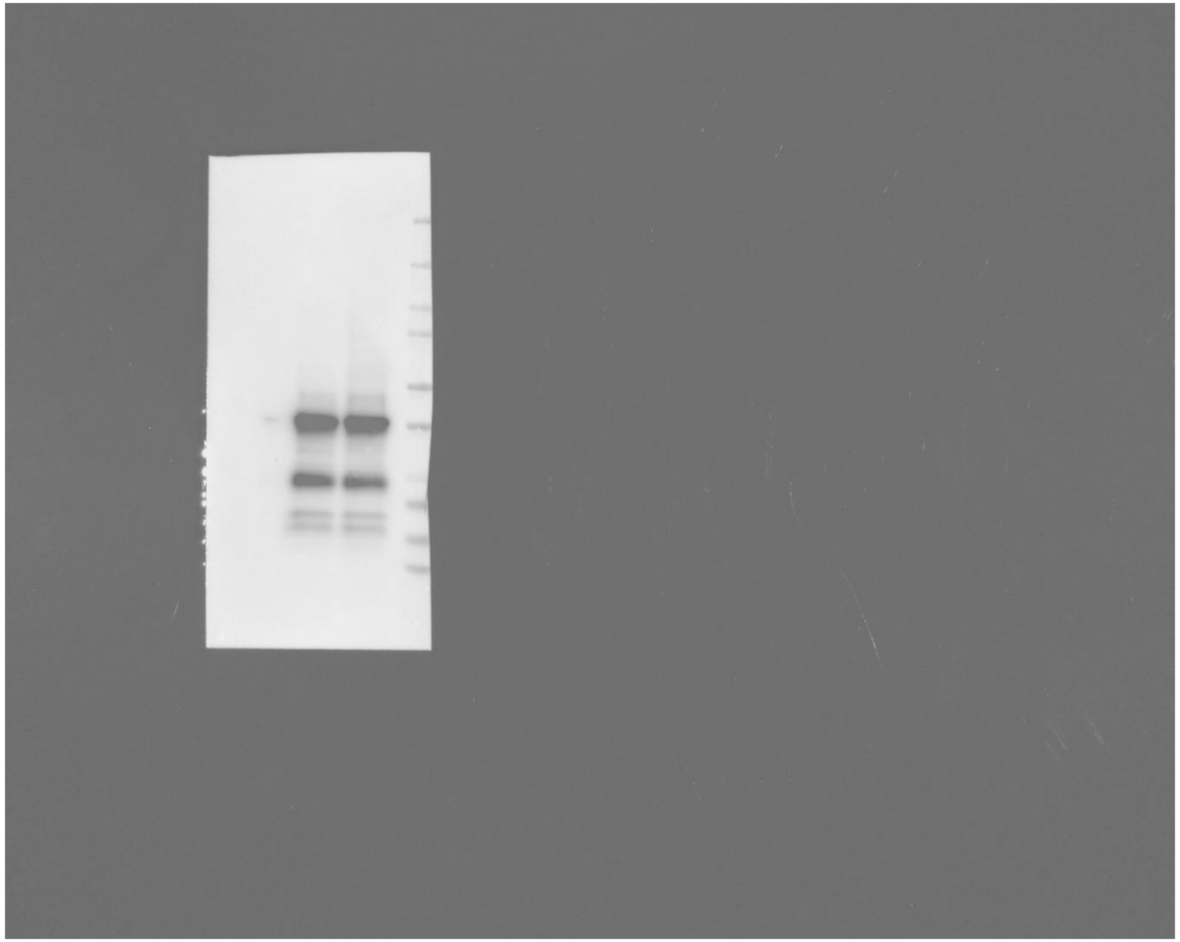

Higher exposure overlaid with marker

Figure 3A MA

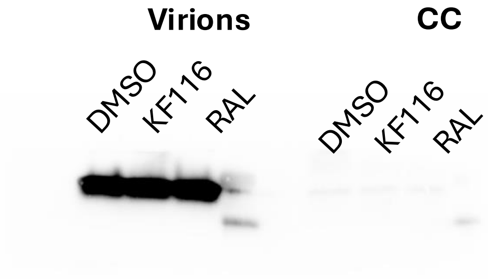

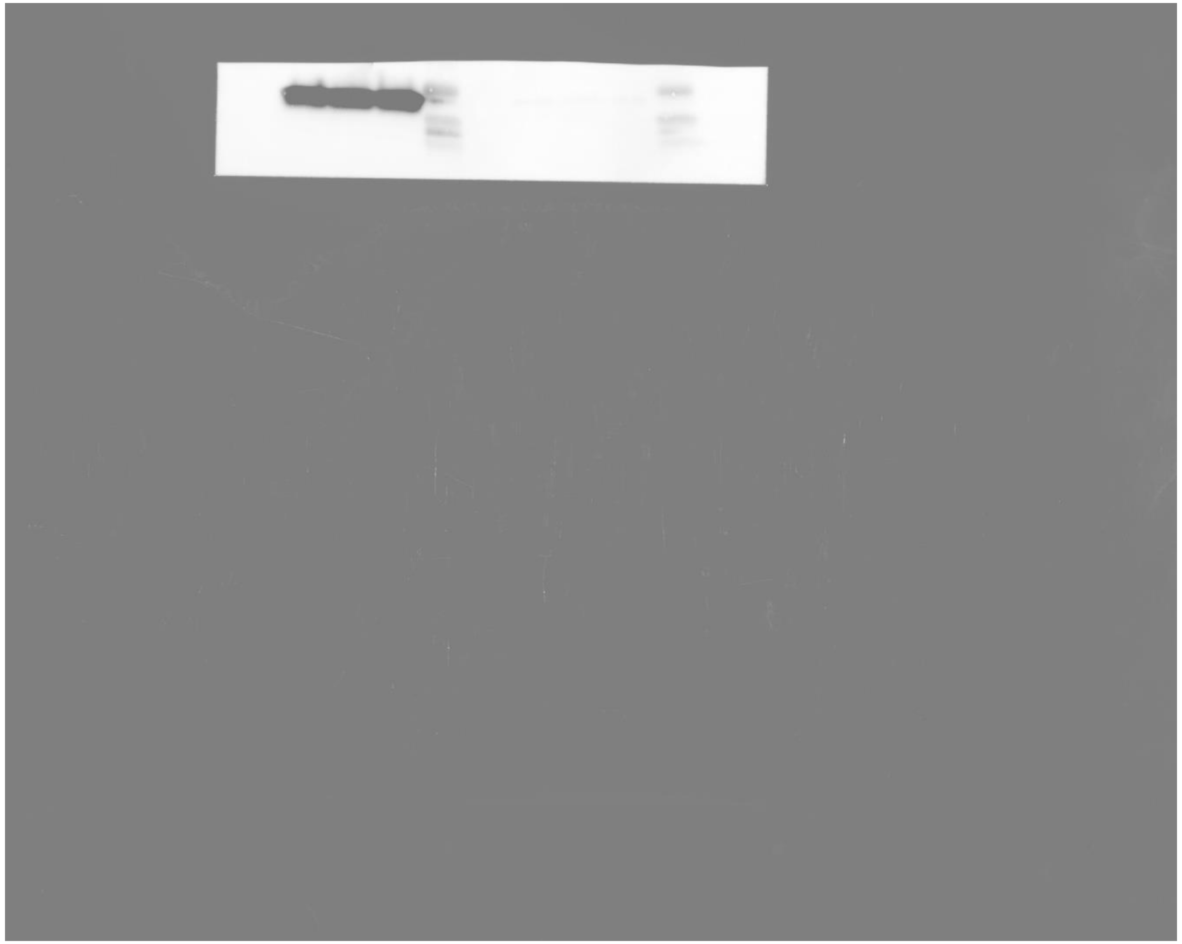

Anti-MA
Detection of MA/p17

Figure 3A CA

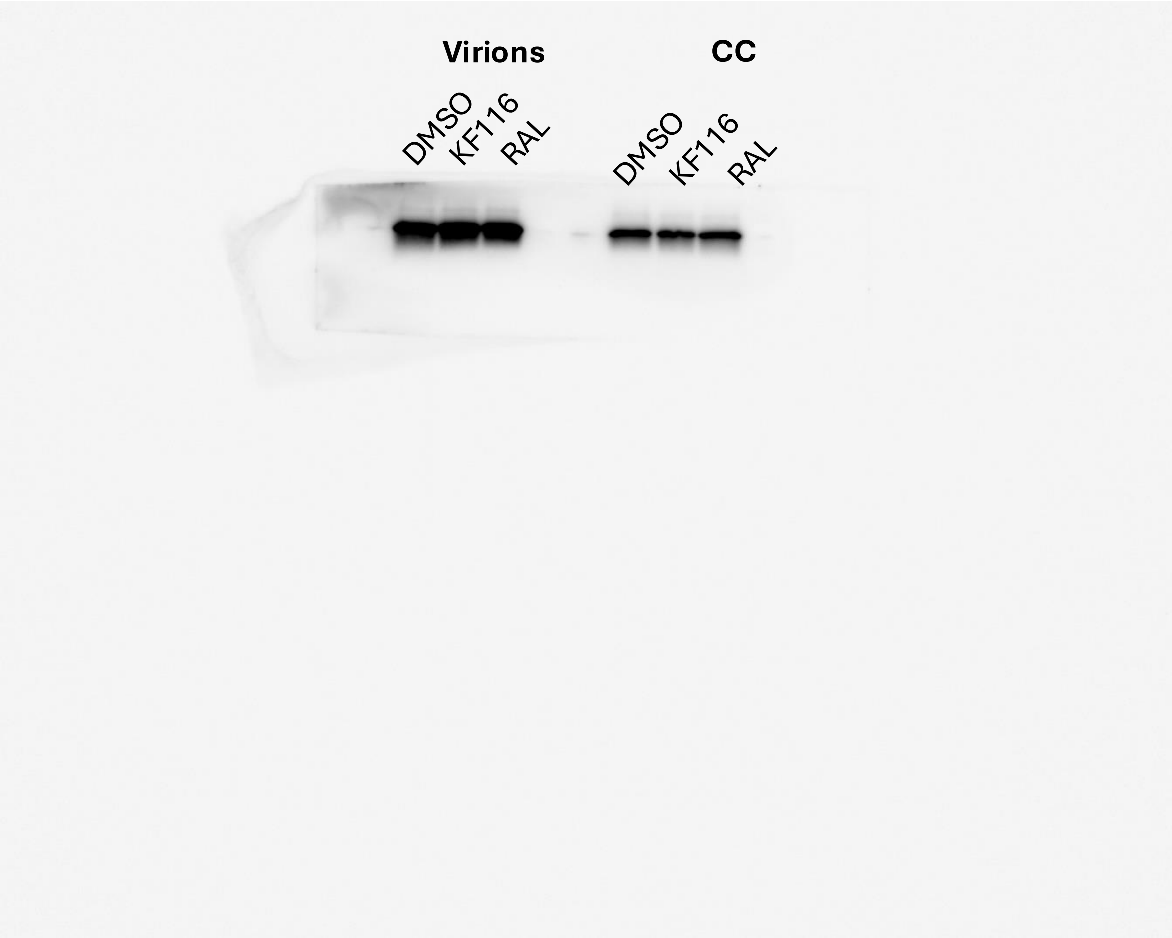

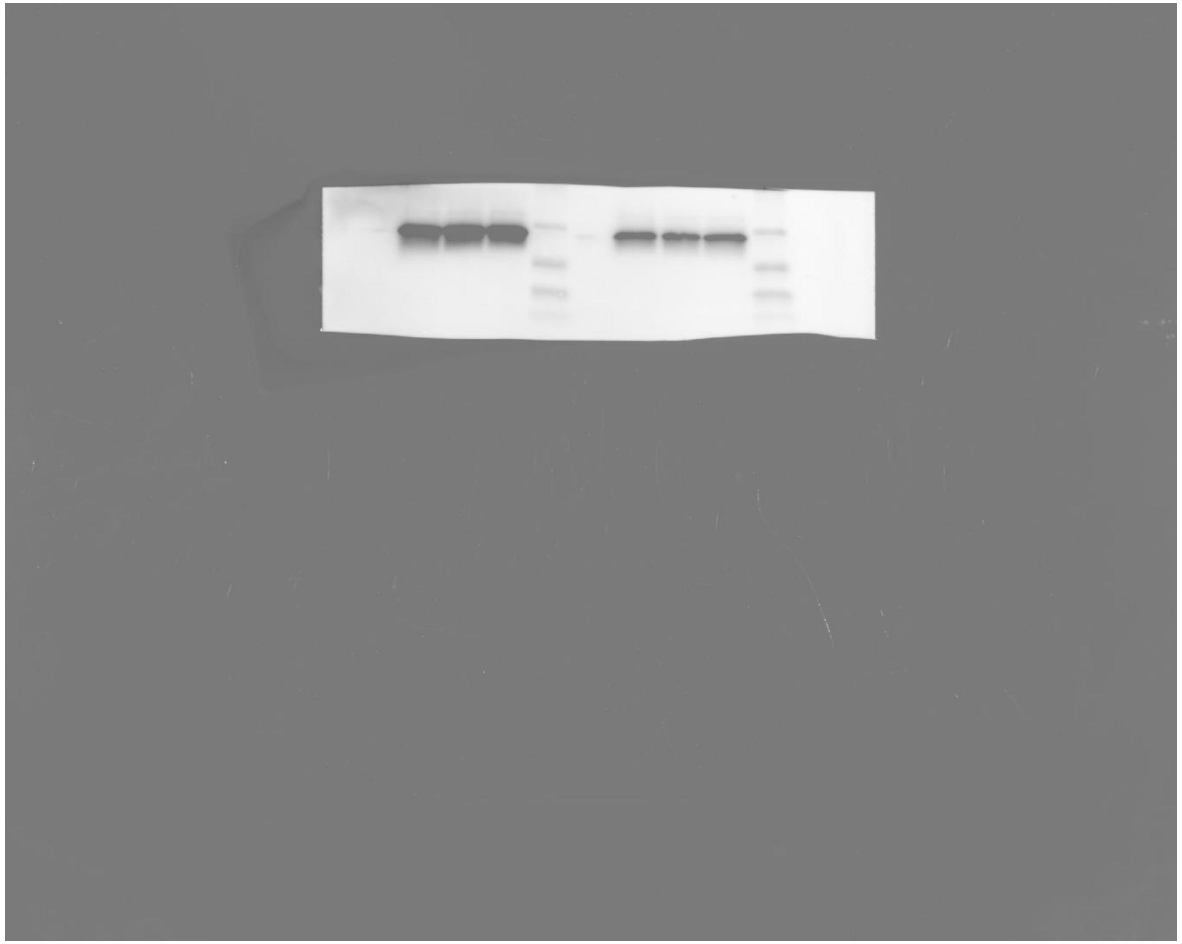

Anti-CA
Detection of CA/p24

Figure 3A NC

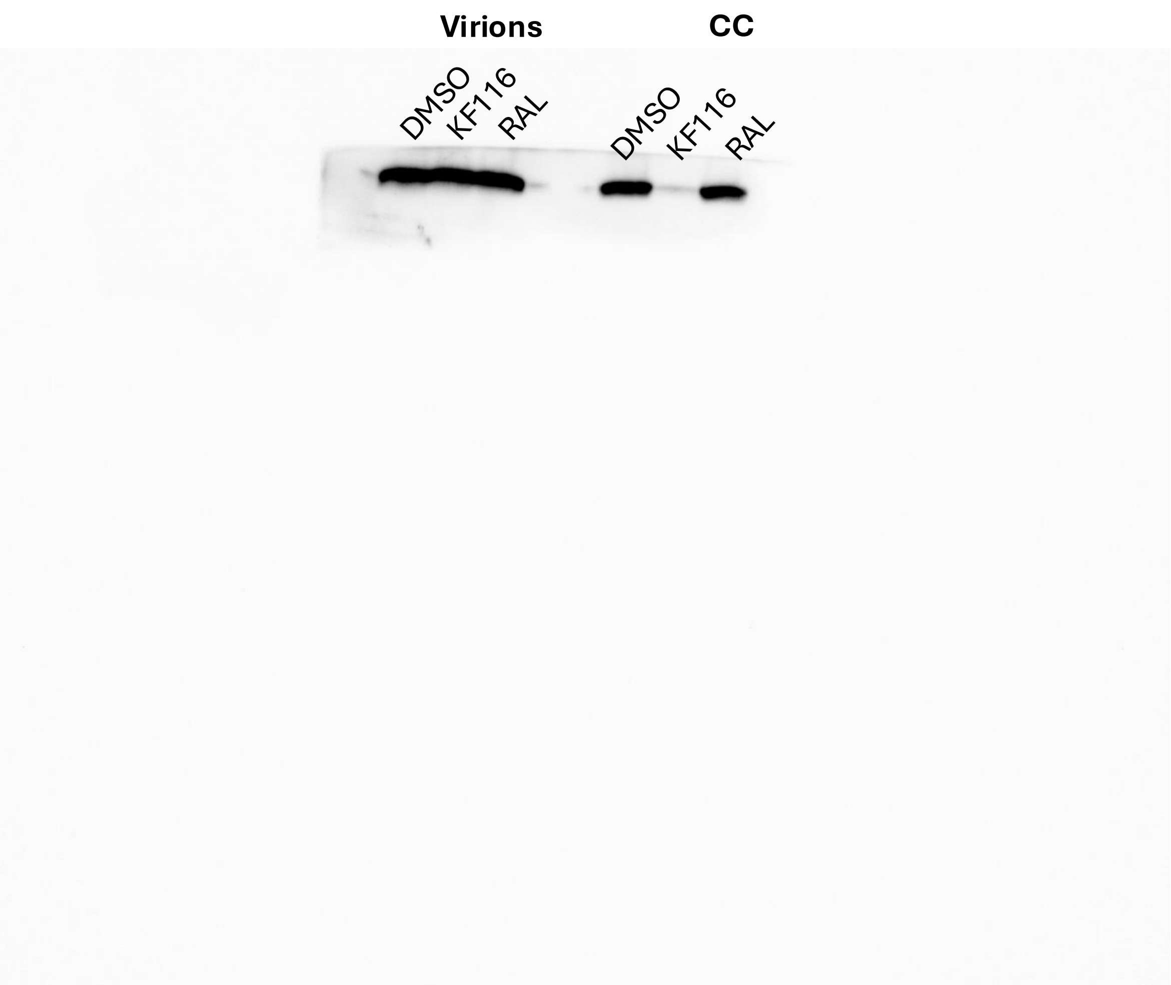

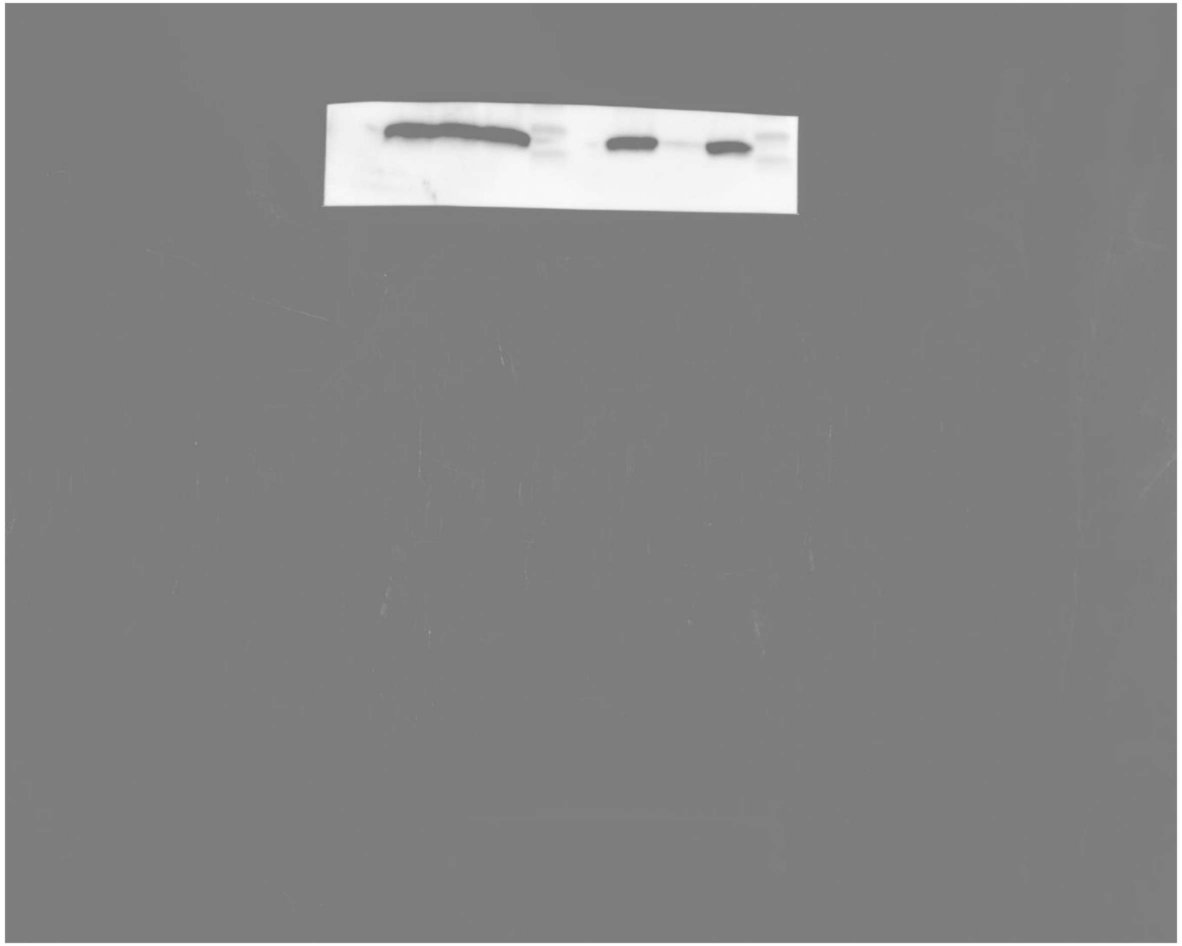

Anti-NC
Detection of NC

Figure 3A RT

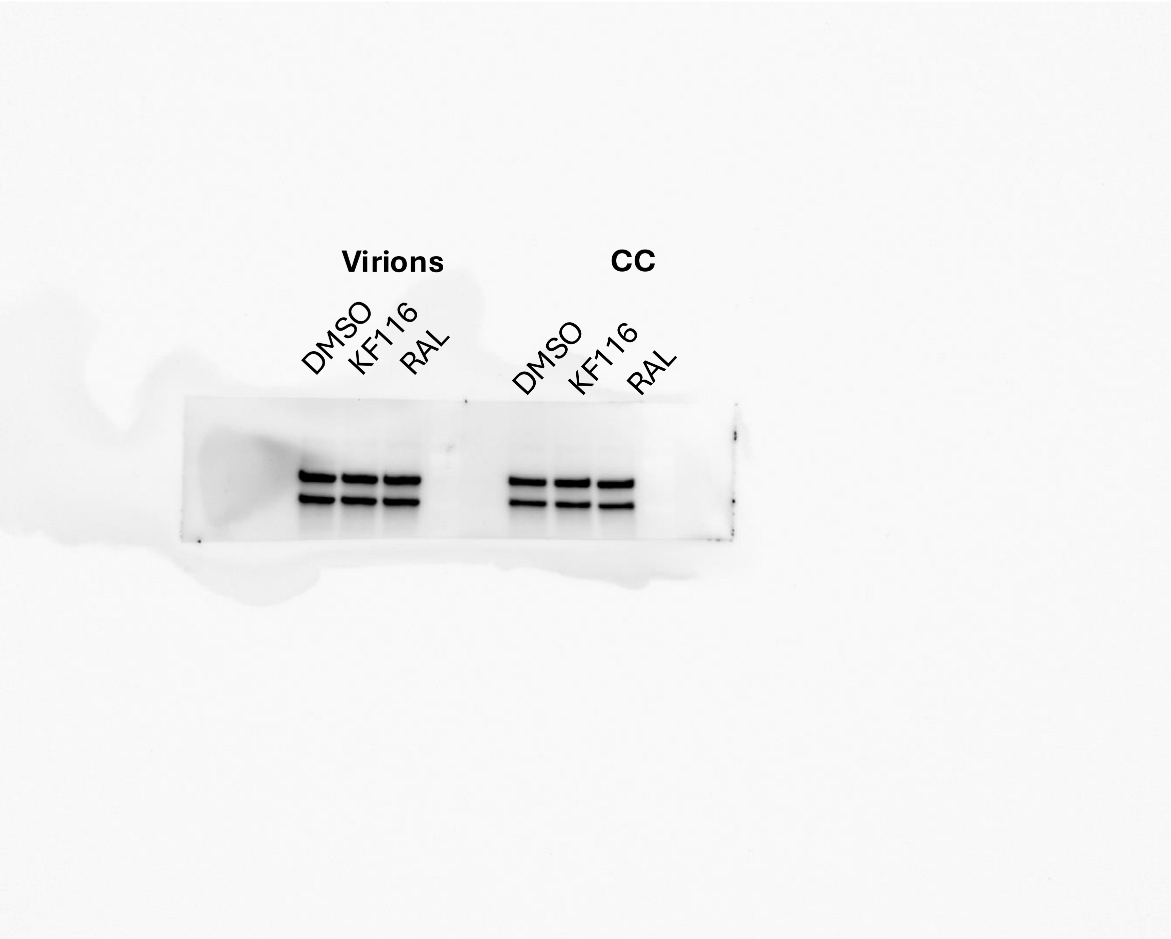

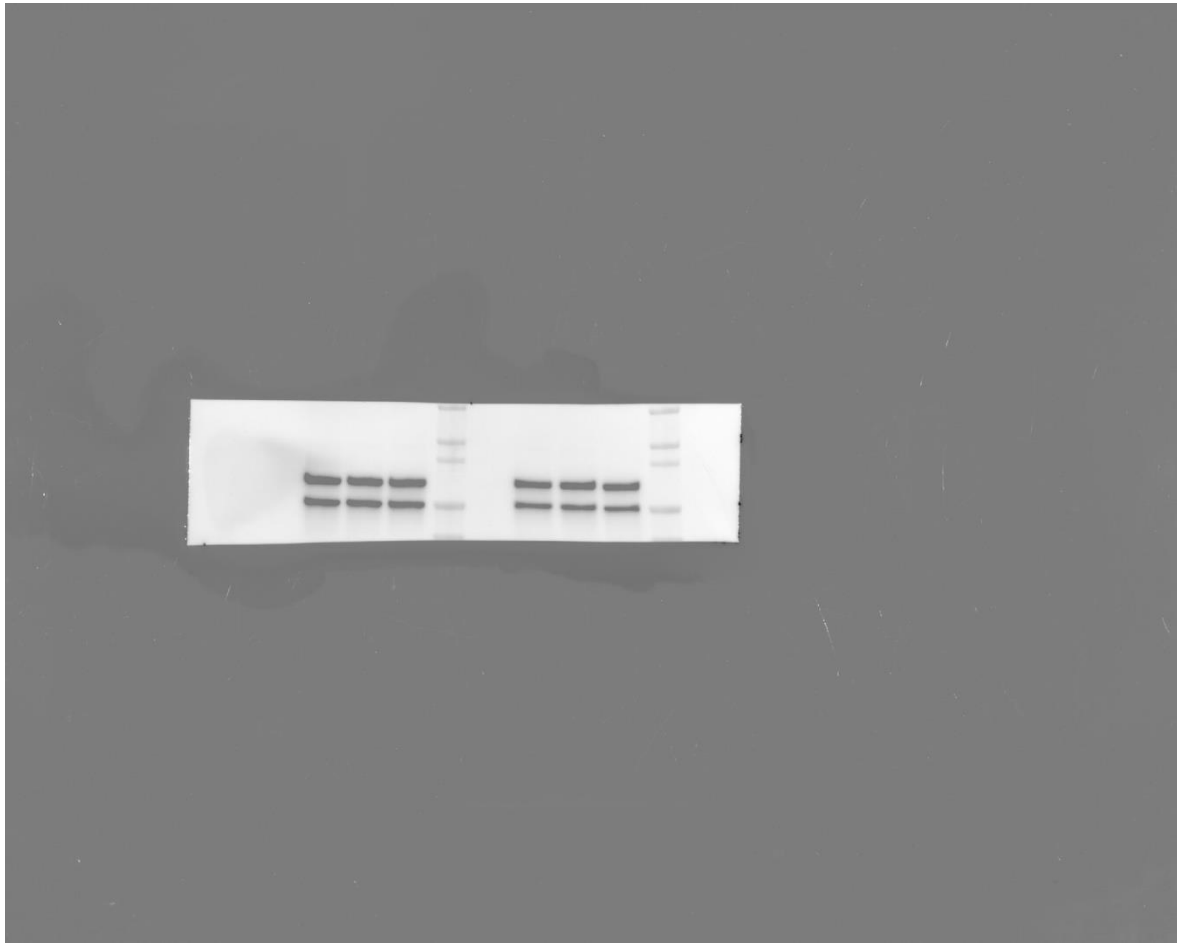

Anti-RT
Detection of RT heterodimer p66/p51

Figure 3A IN

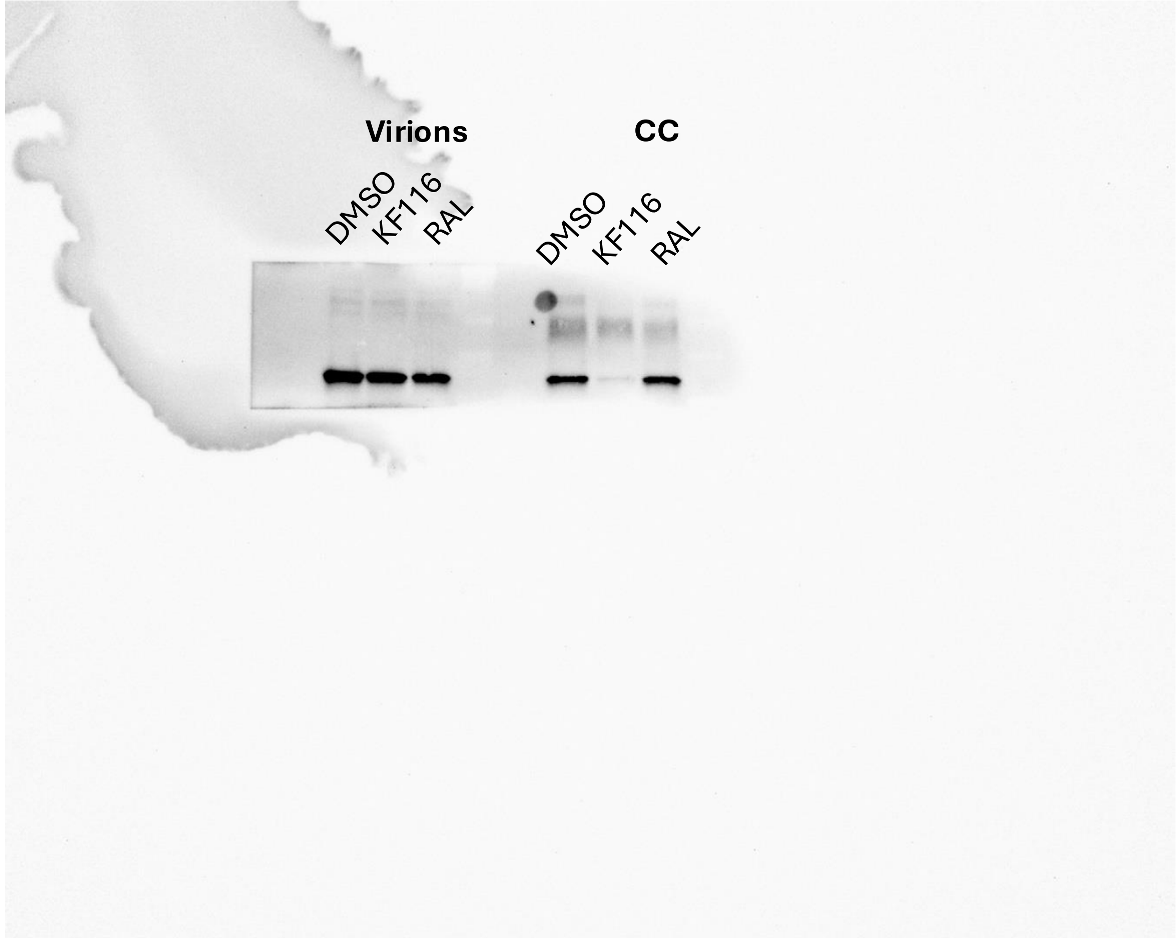

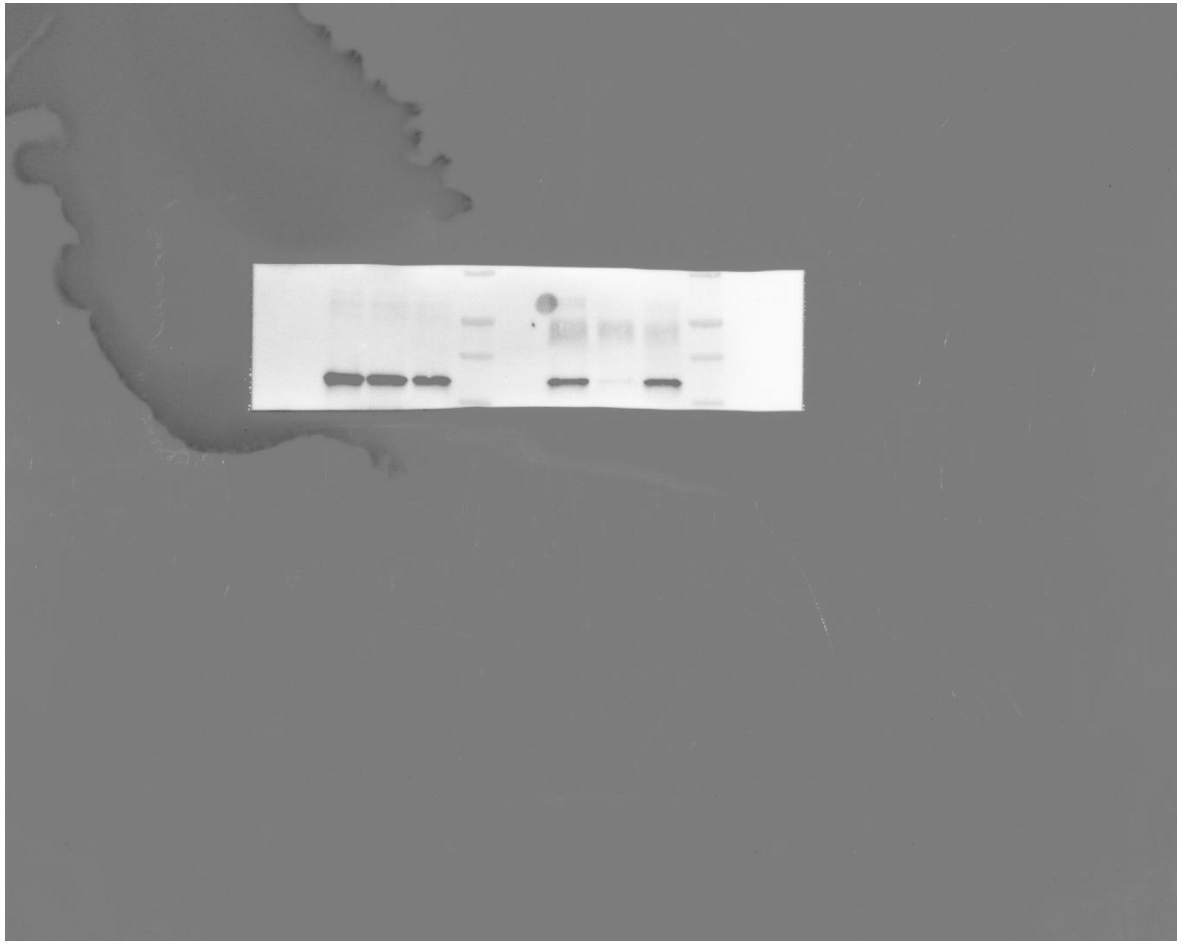

Anti-IN
Detection of IN/p32

Figure 3A YFP-Vpr

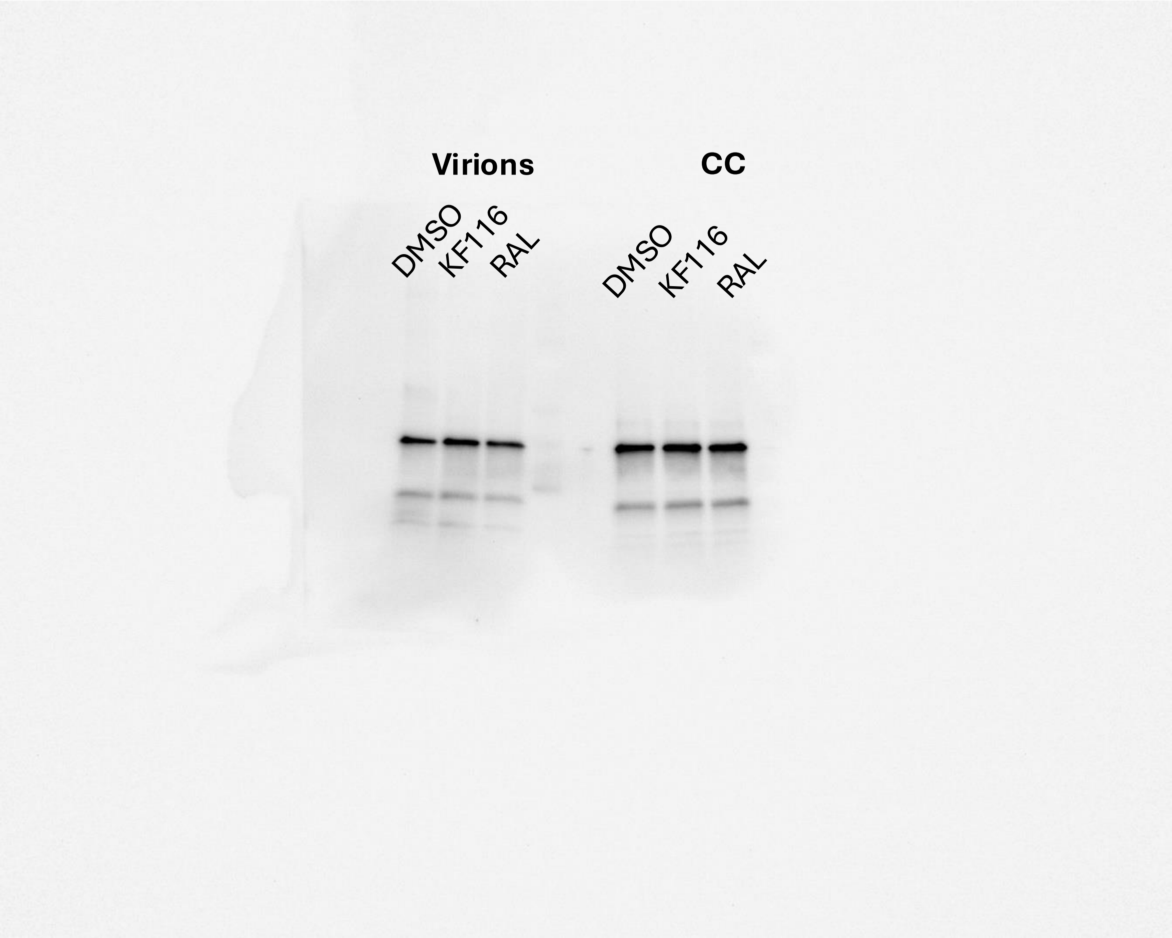

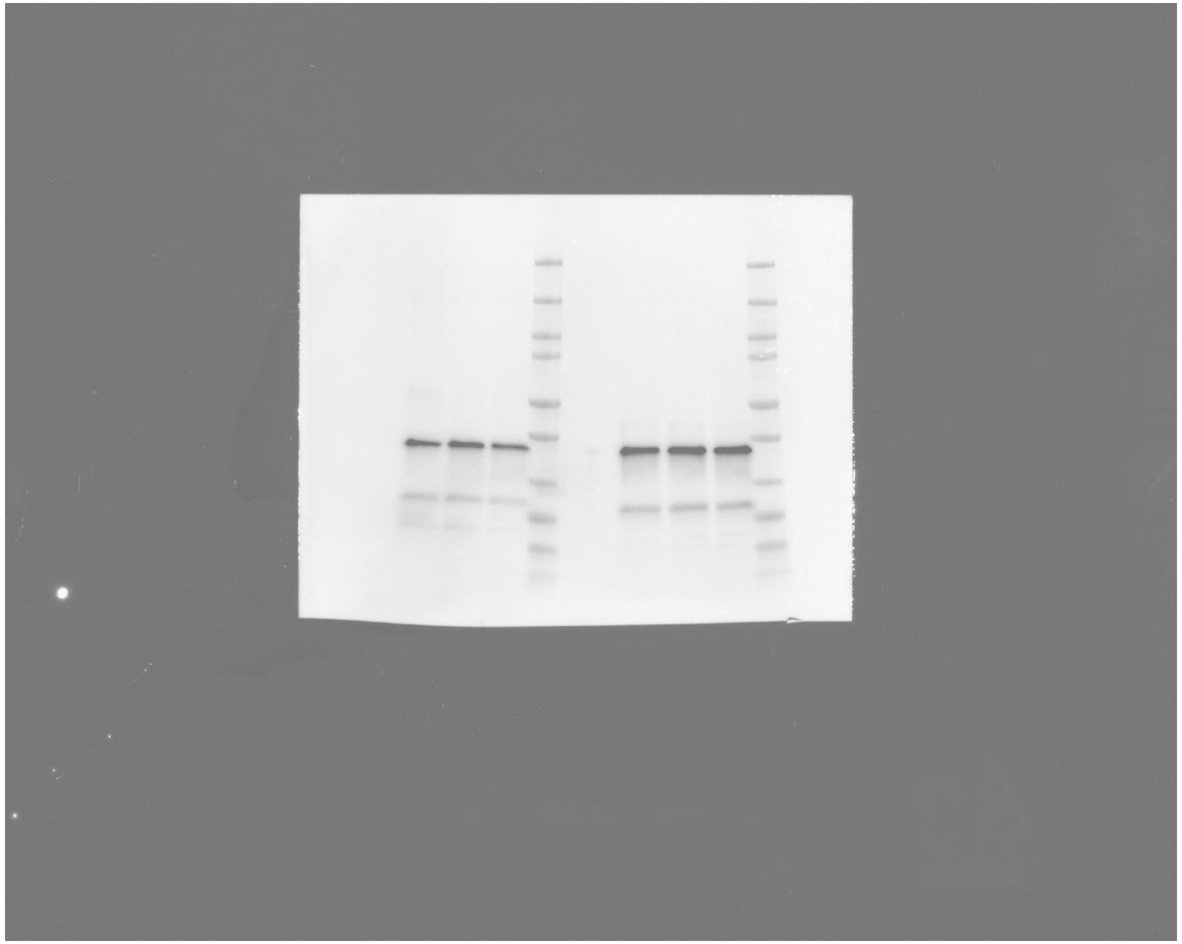

Anti-YFP
Detection of YFP-Vpr

Figure 4A CA

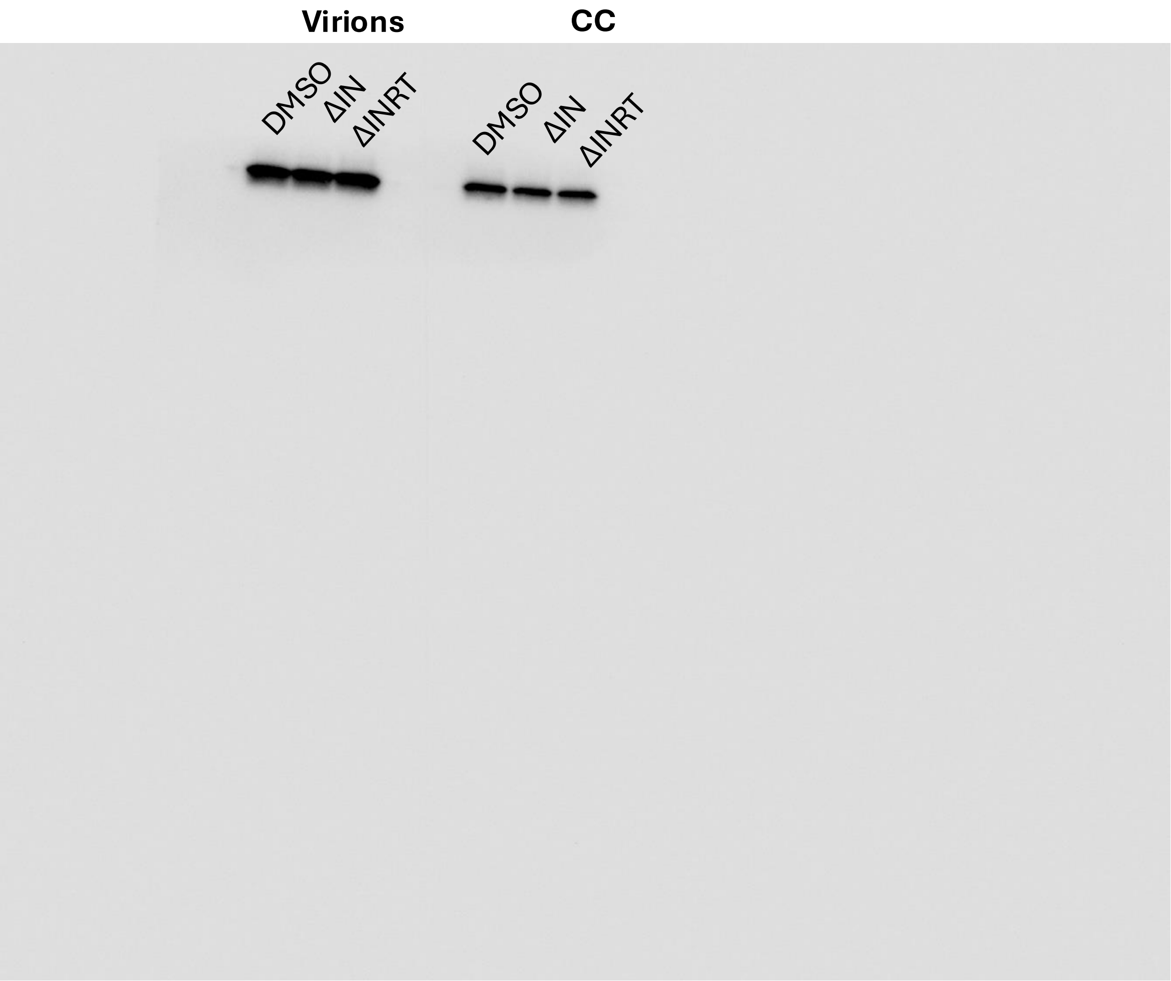

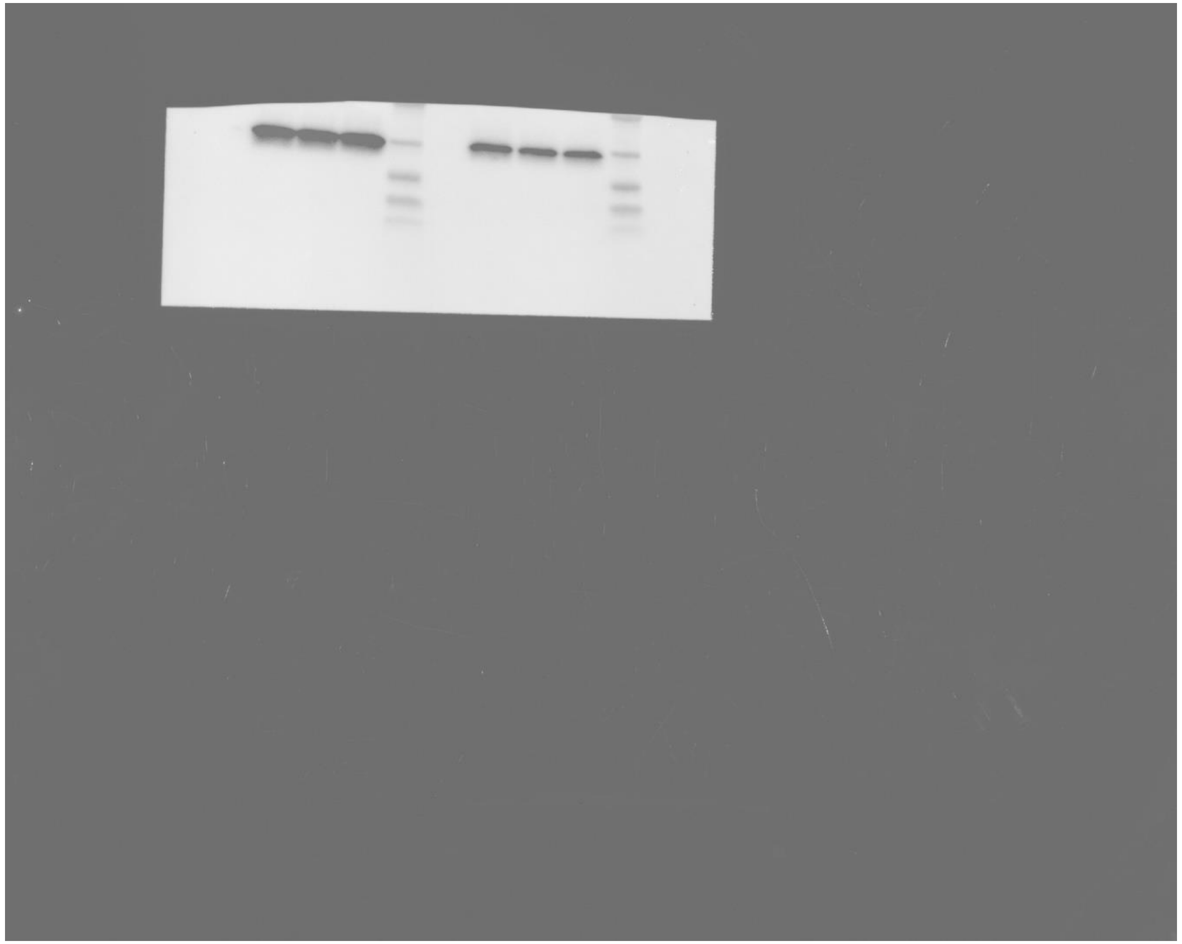

Anti-CA
Detection of CA/p24

Figure 4A NC

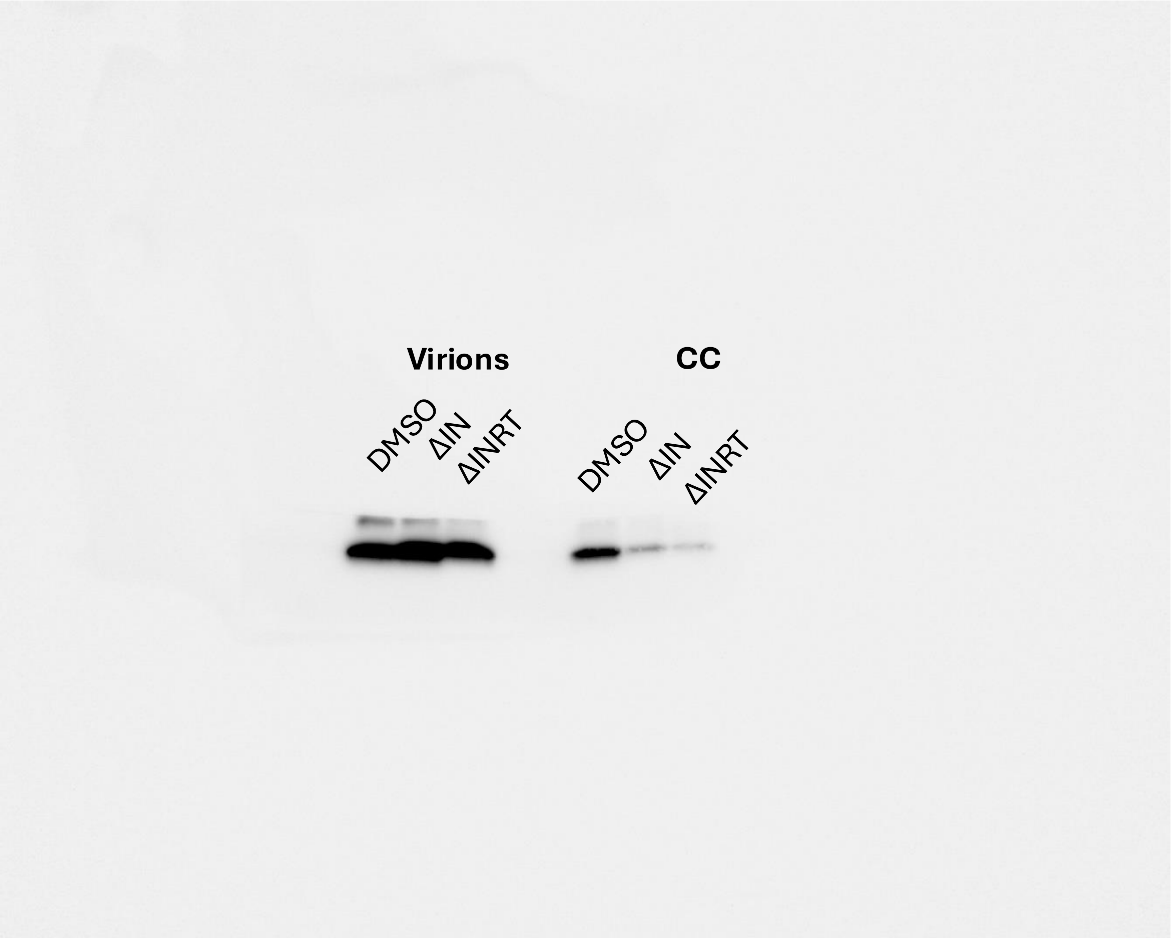

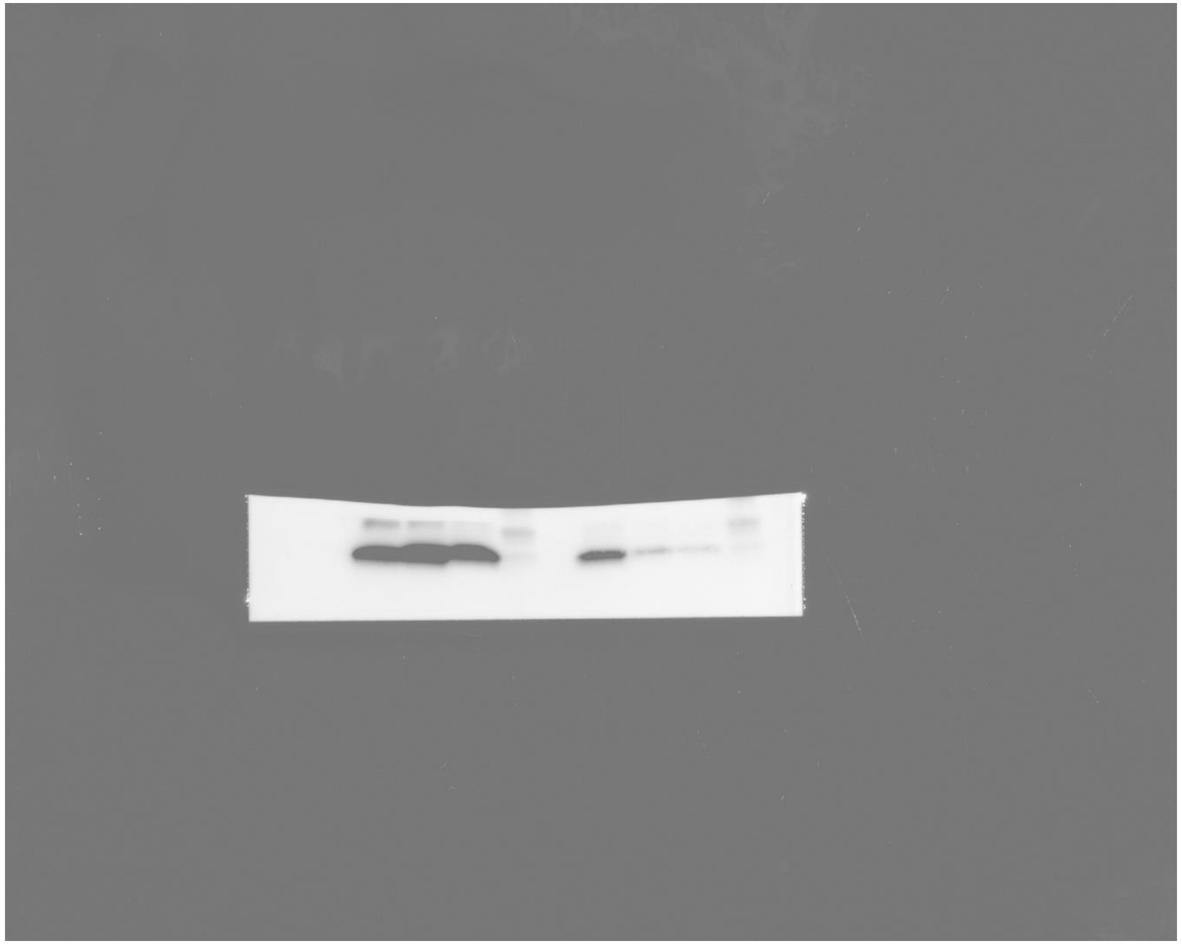

Anti-NC
Detection of NC

Figure 4A RT

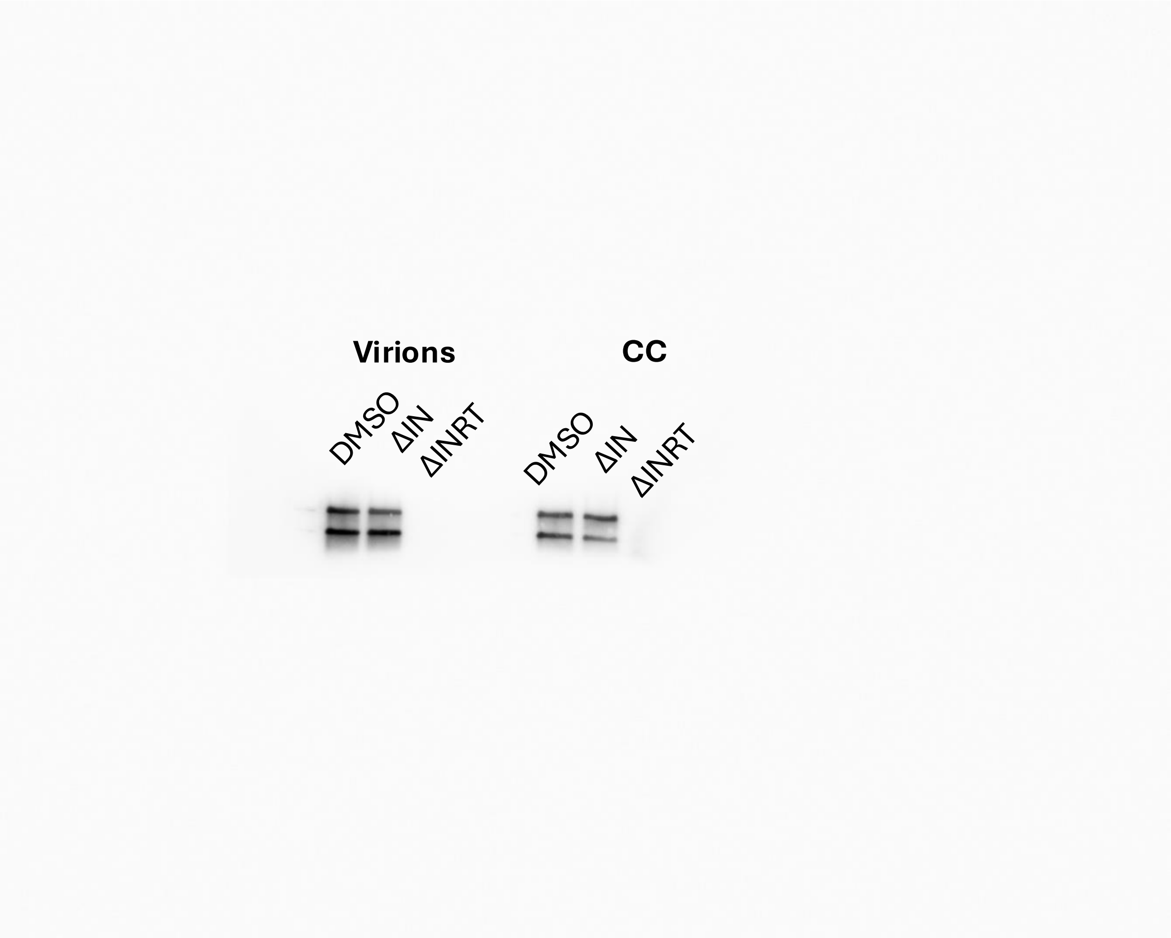

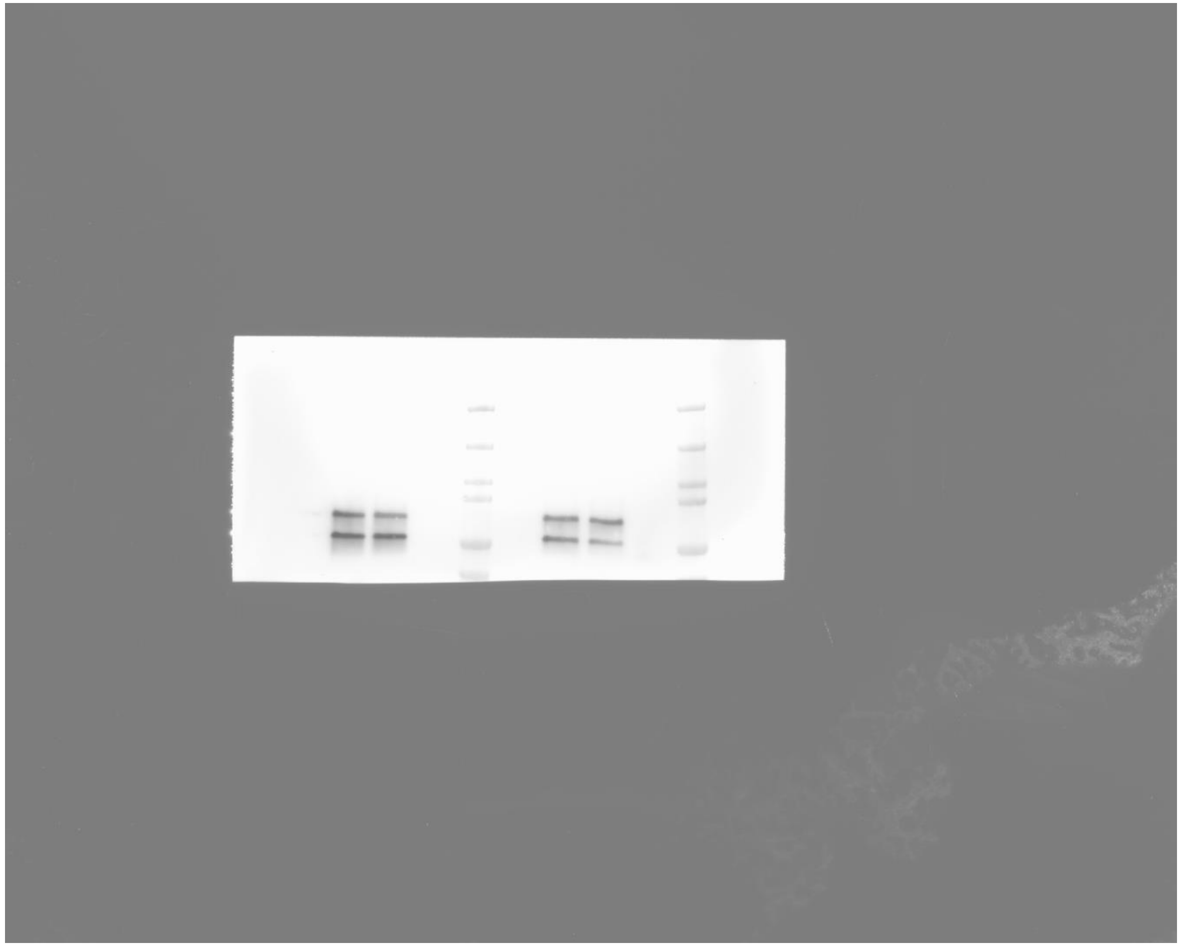

Anti-RT
Detection of RT heterodimer p66/p51

Figure 4A IN

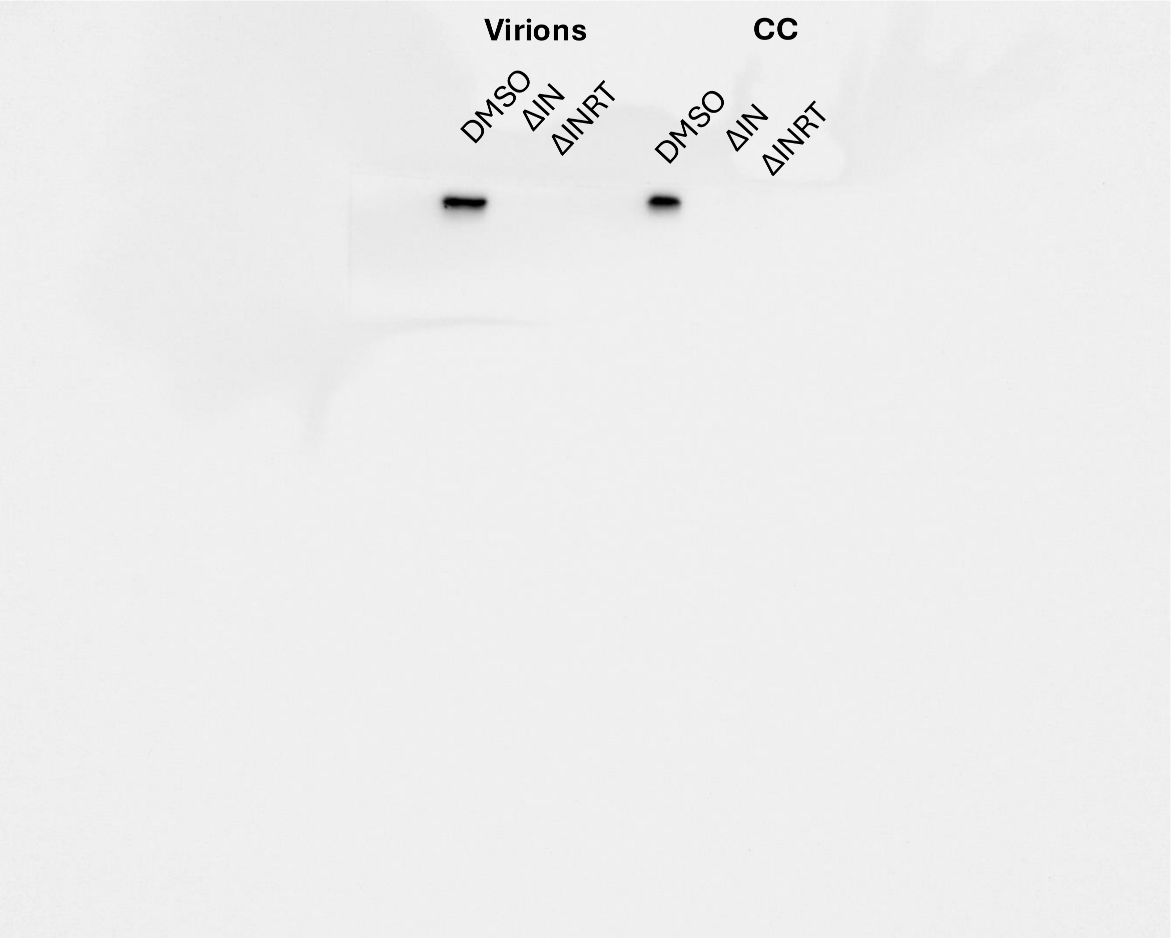

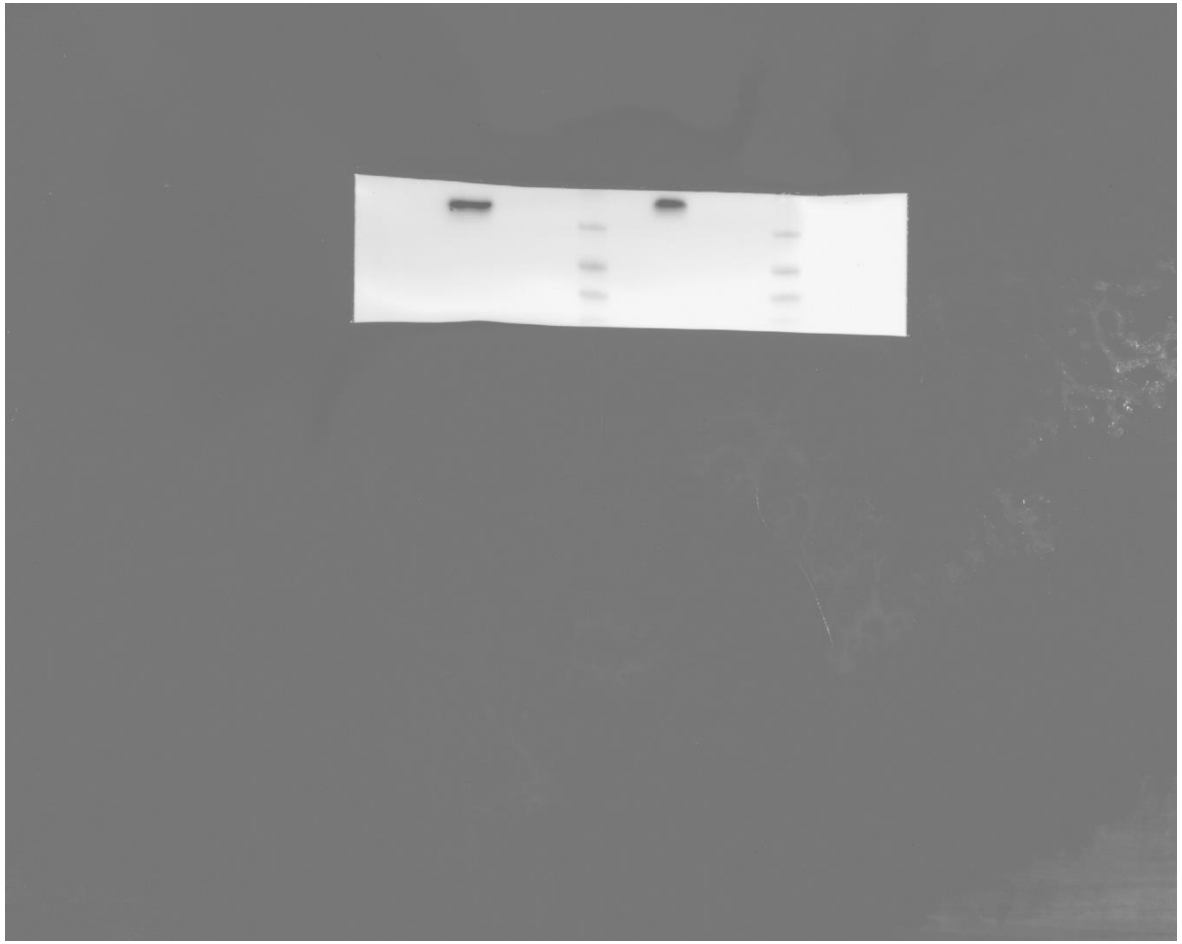

Anti-IN
Detection of IN/p32

Figure 5B CA Virus

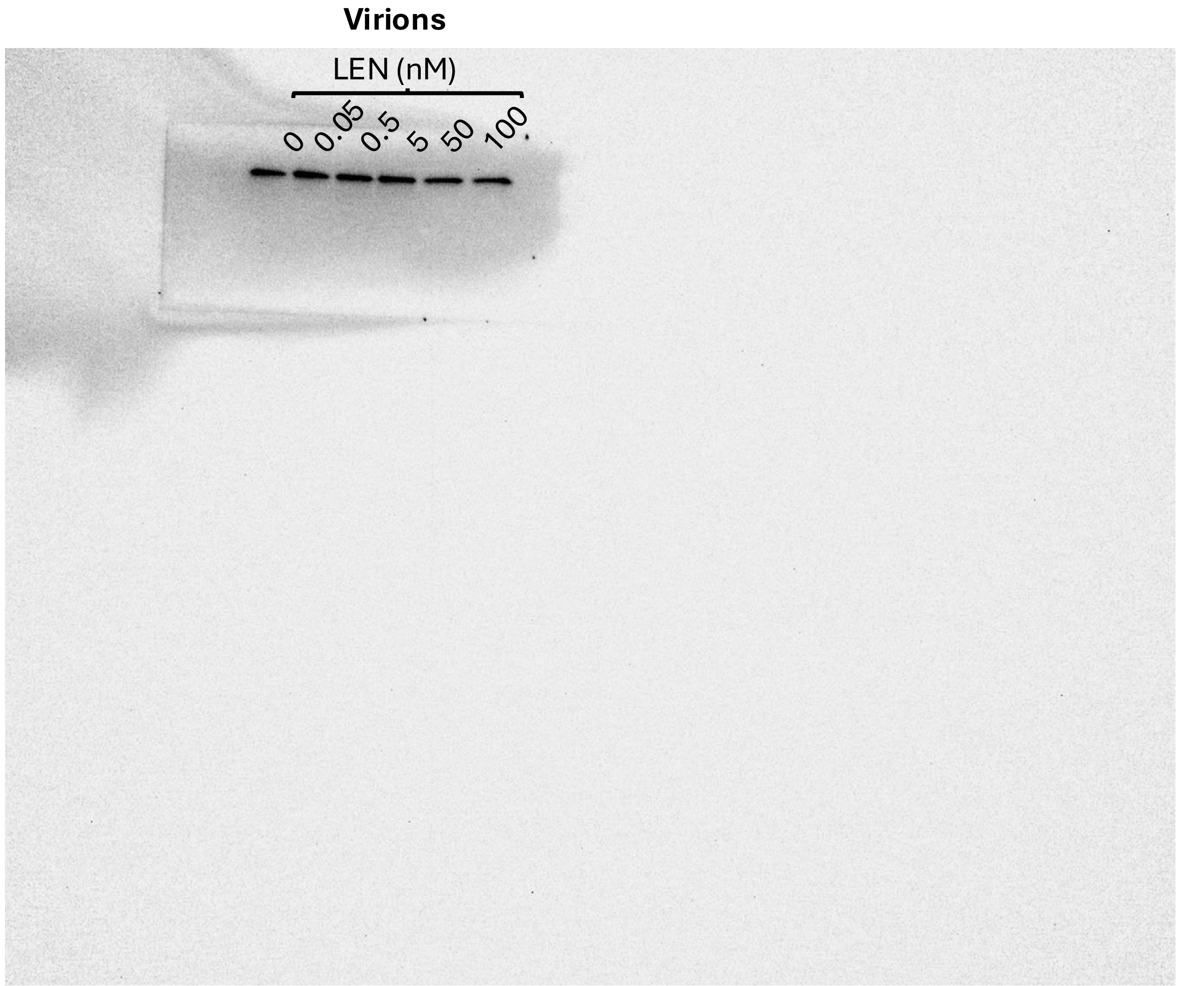

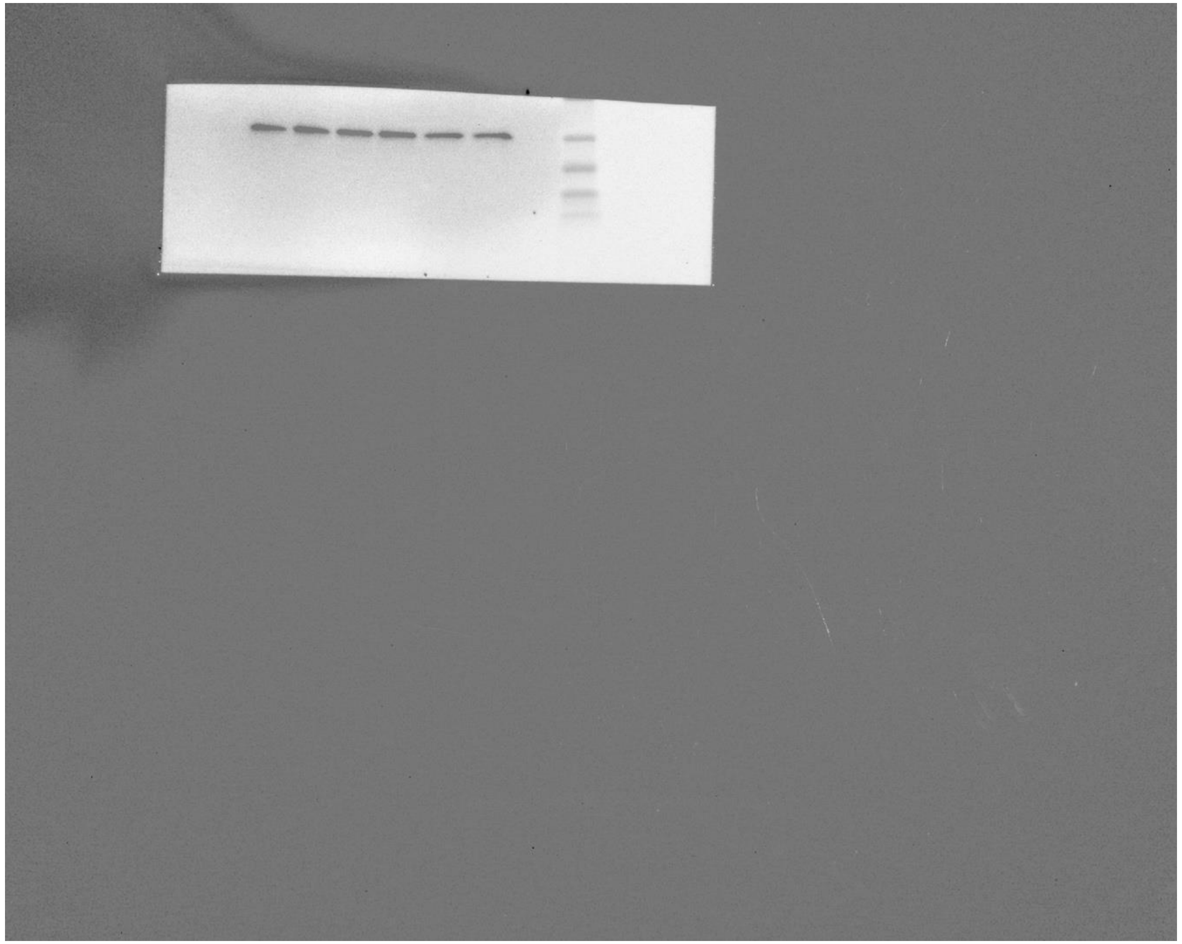

Anti-CA
Detection of CA/p24

Figure 5B NC Virus

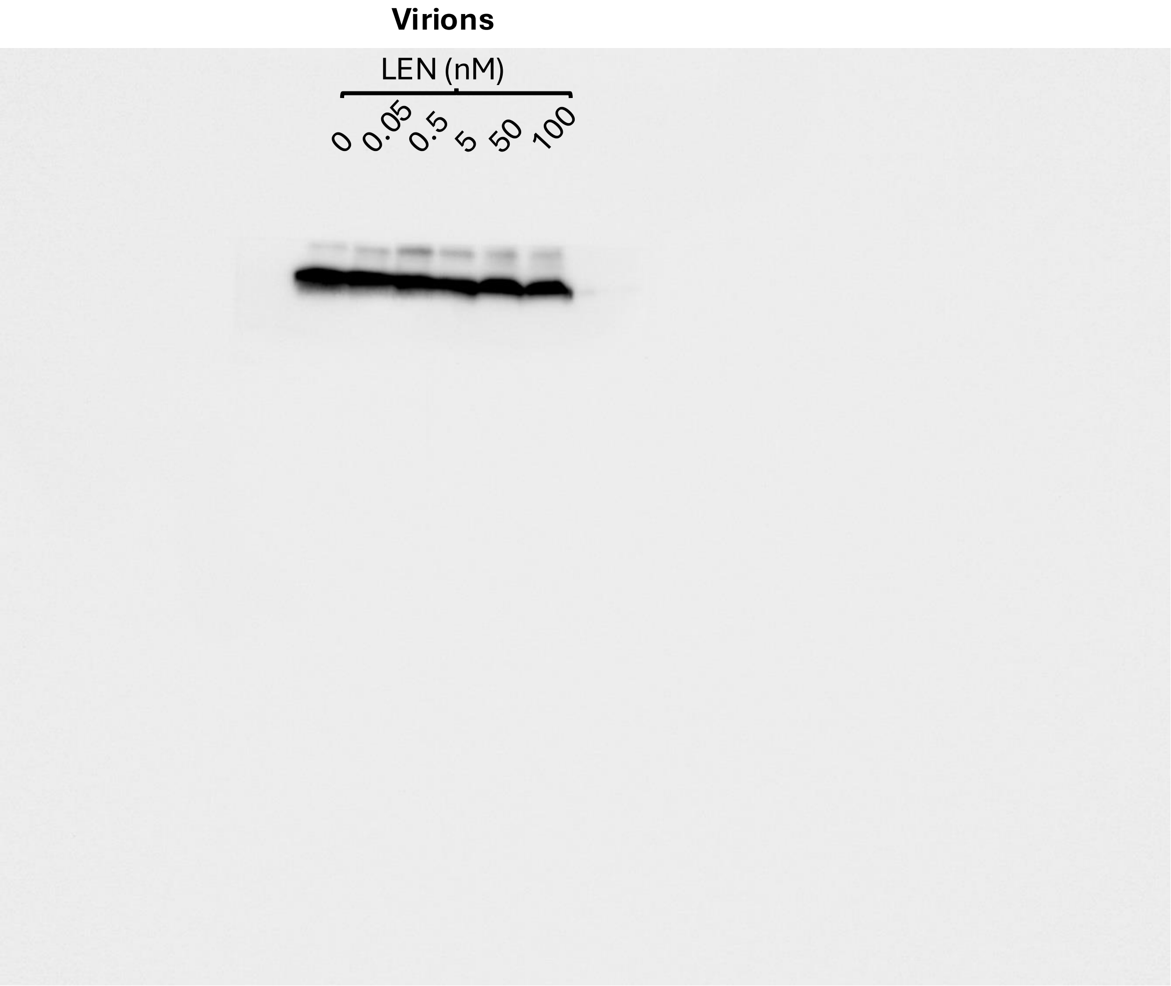

Anti-NC
Detection of NC

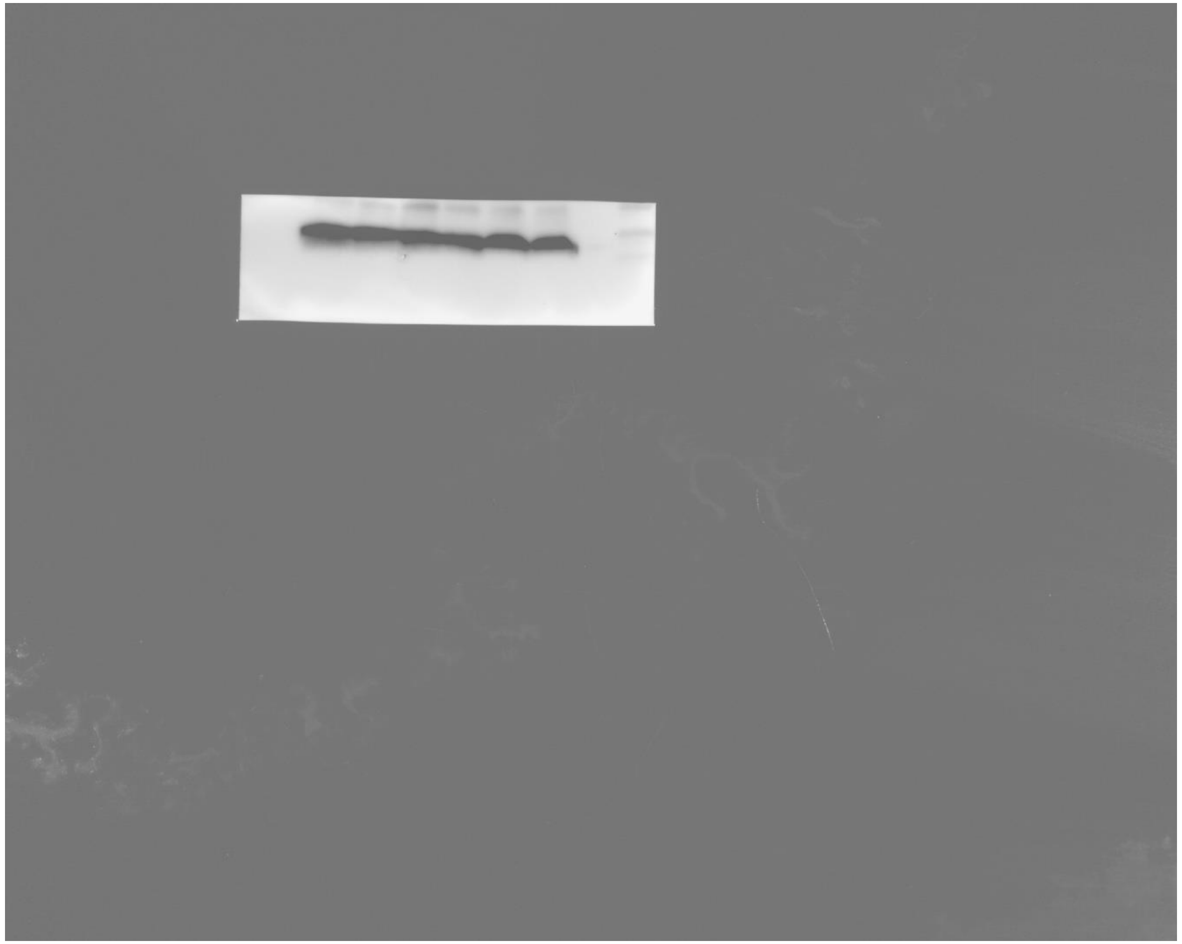

Figure 5B RT Virus

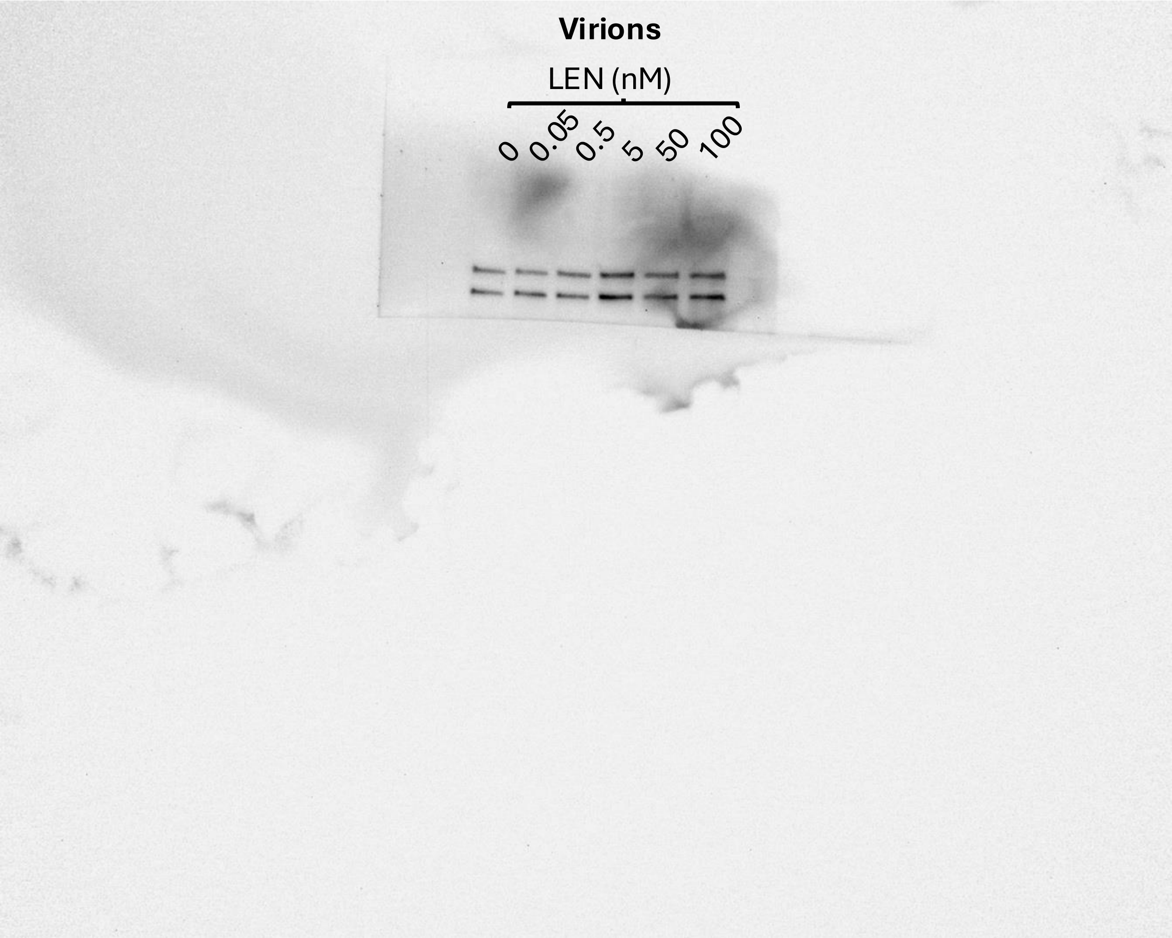

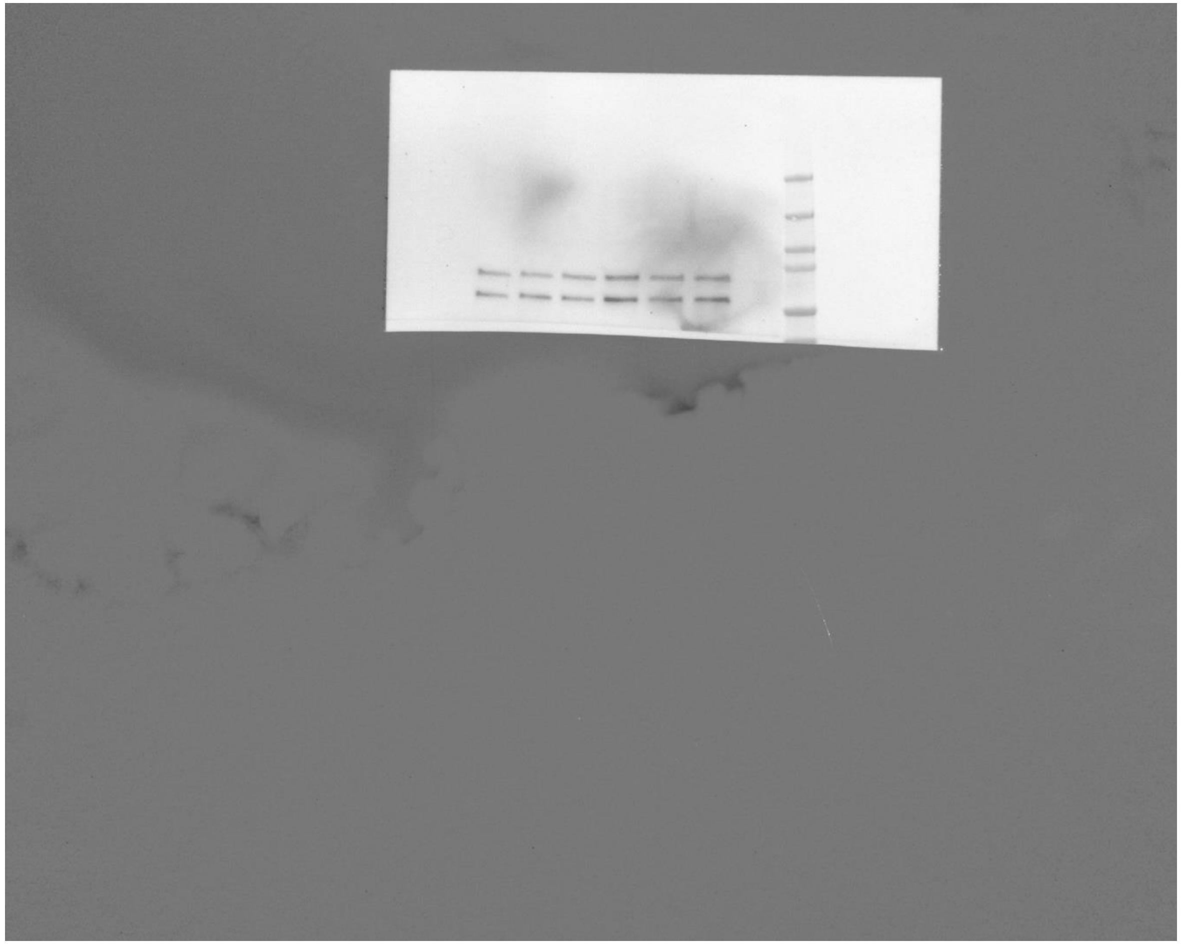

Anti-RT
Detection of RT heterodimer p66/p51

Figure 5B IN Virus

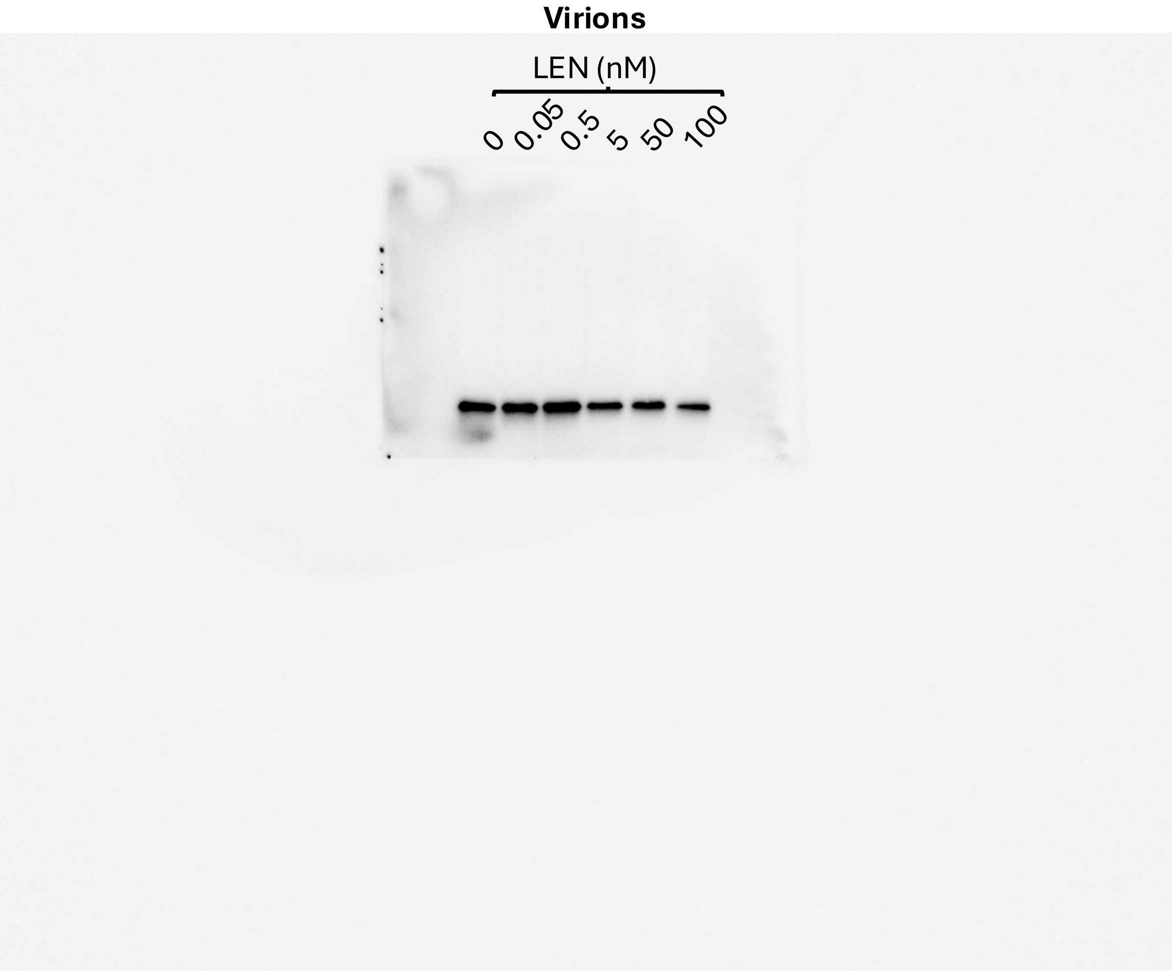

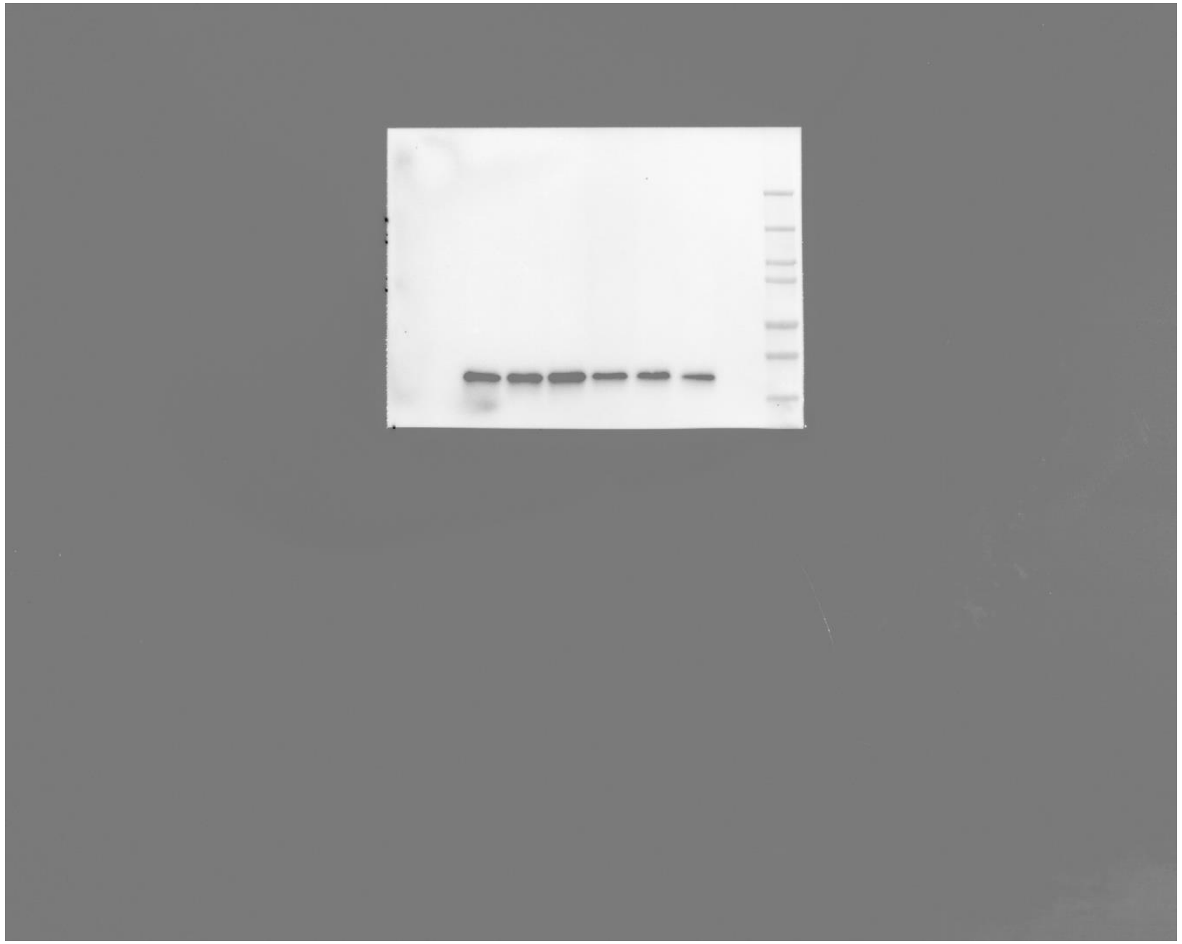

Anti-IN
Detection of IN/p32

Figure 5B CA CC

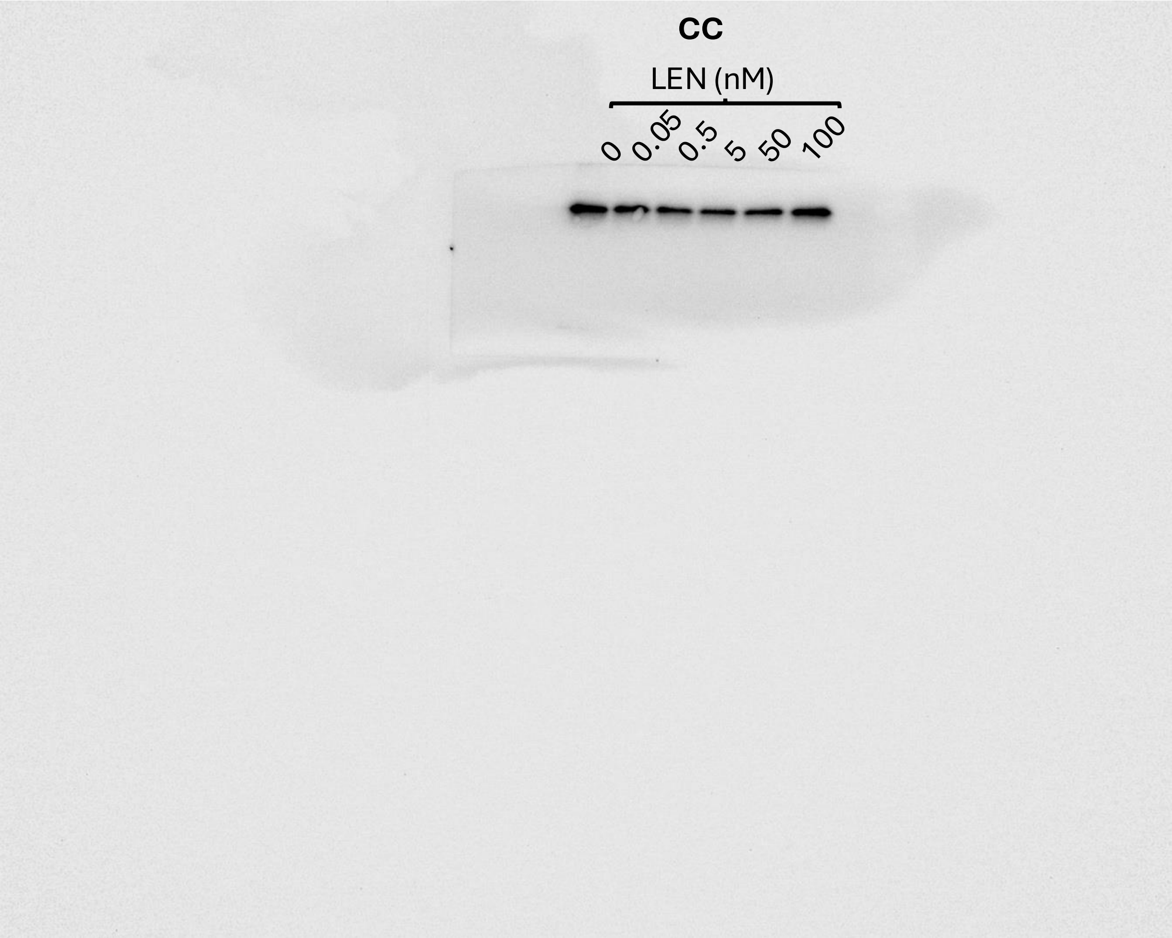

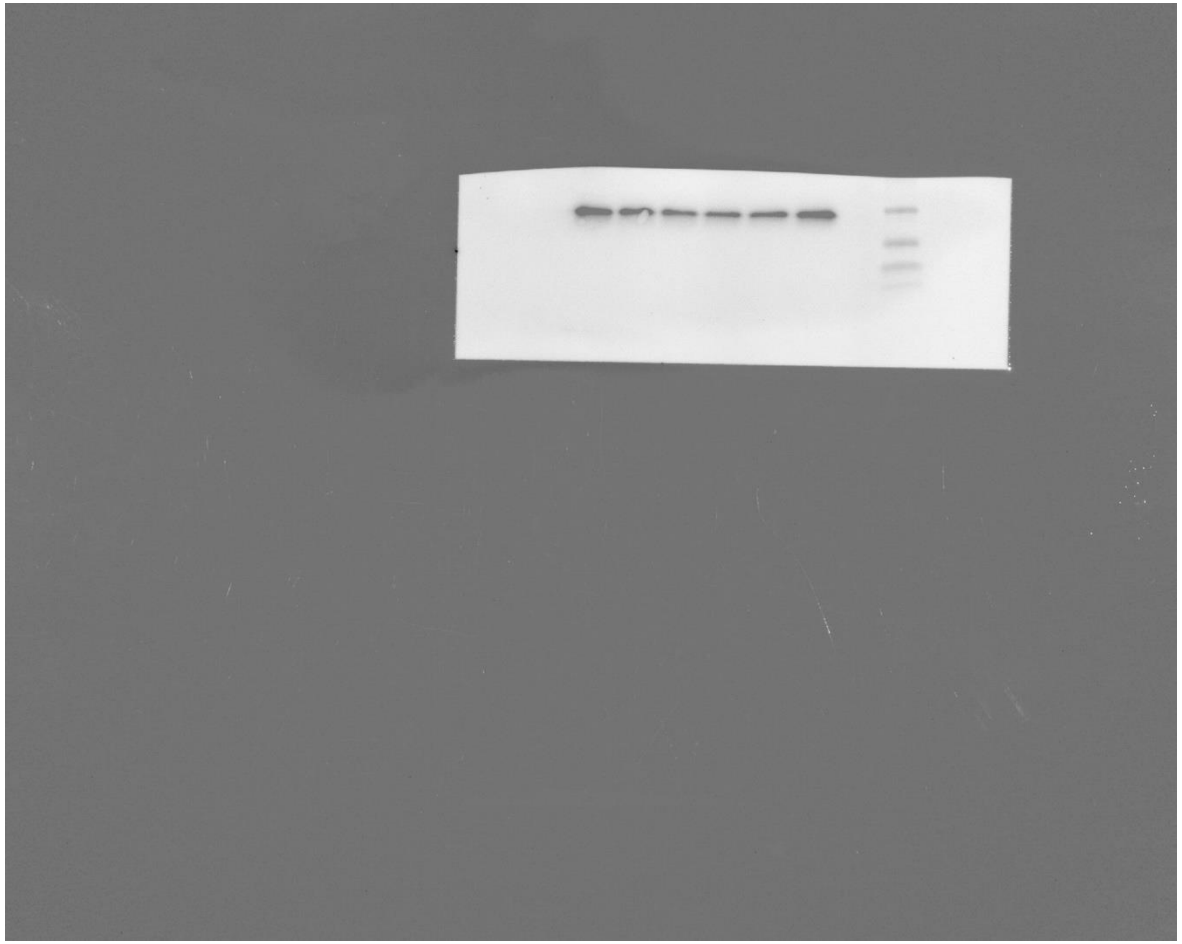

Anti-CA
Detection of CA/p24

Figure 5B NC CC

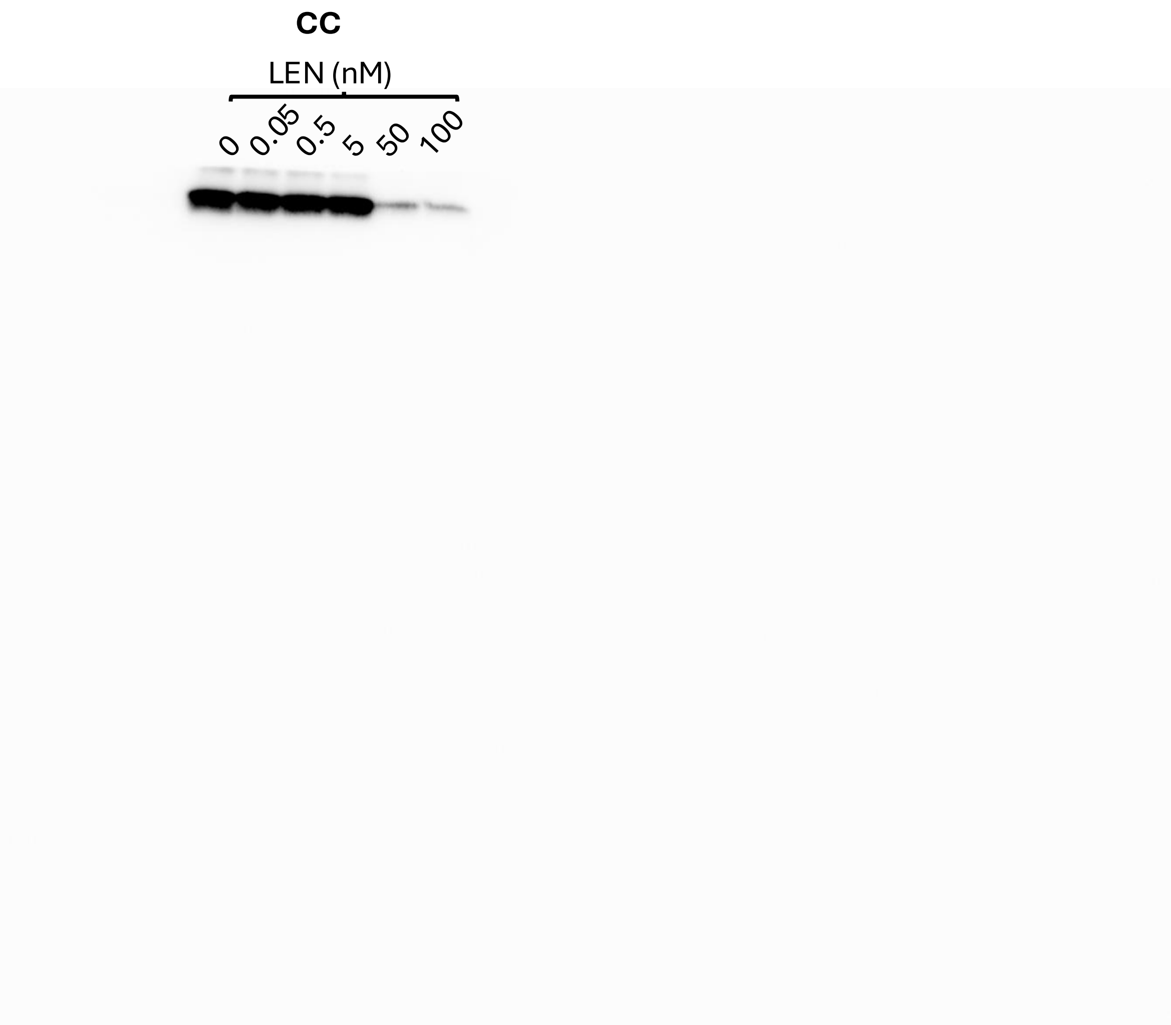

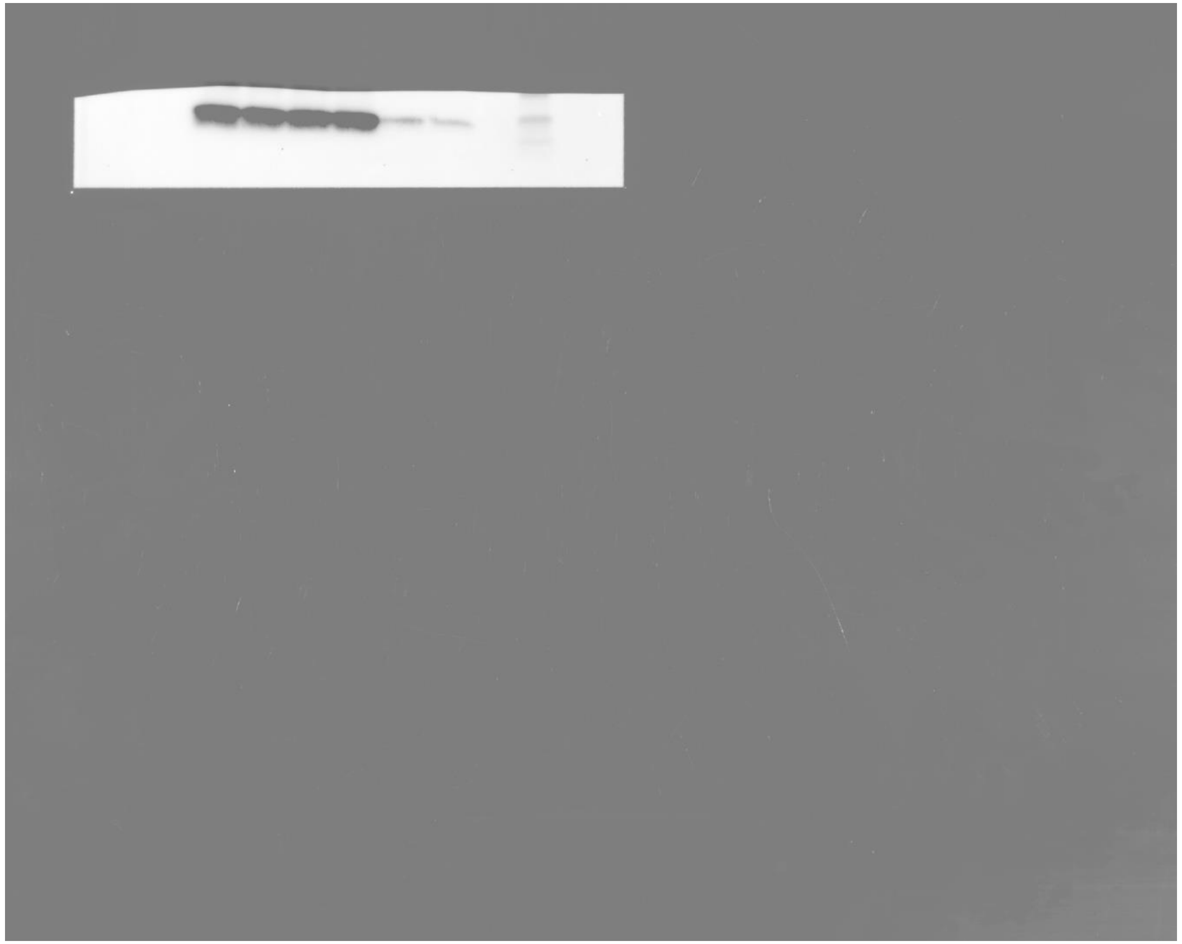

Anti-NC
Detection of NC

Figure 5B RT CC

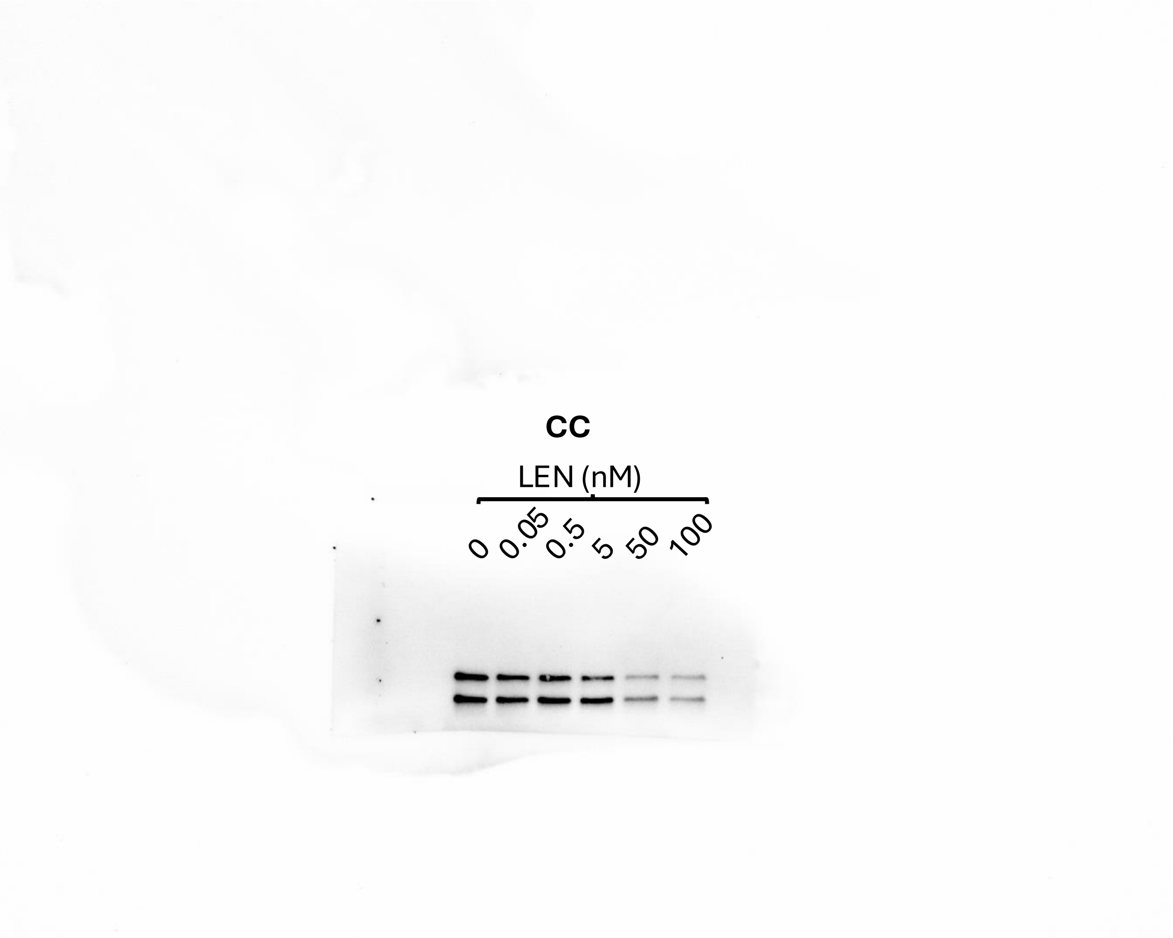

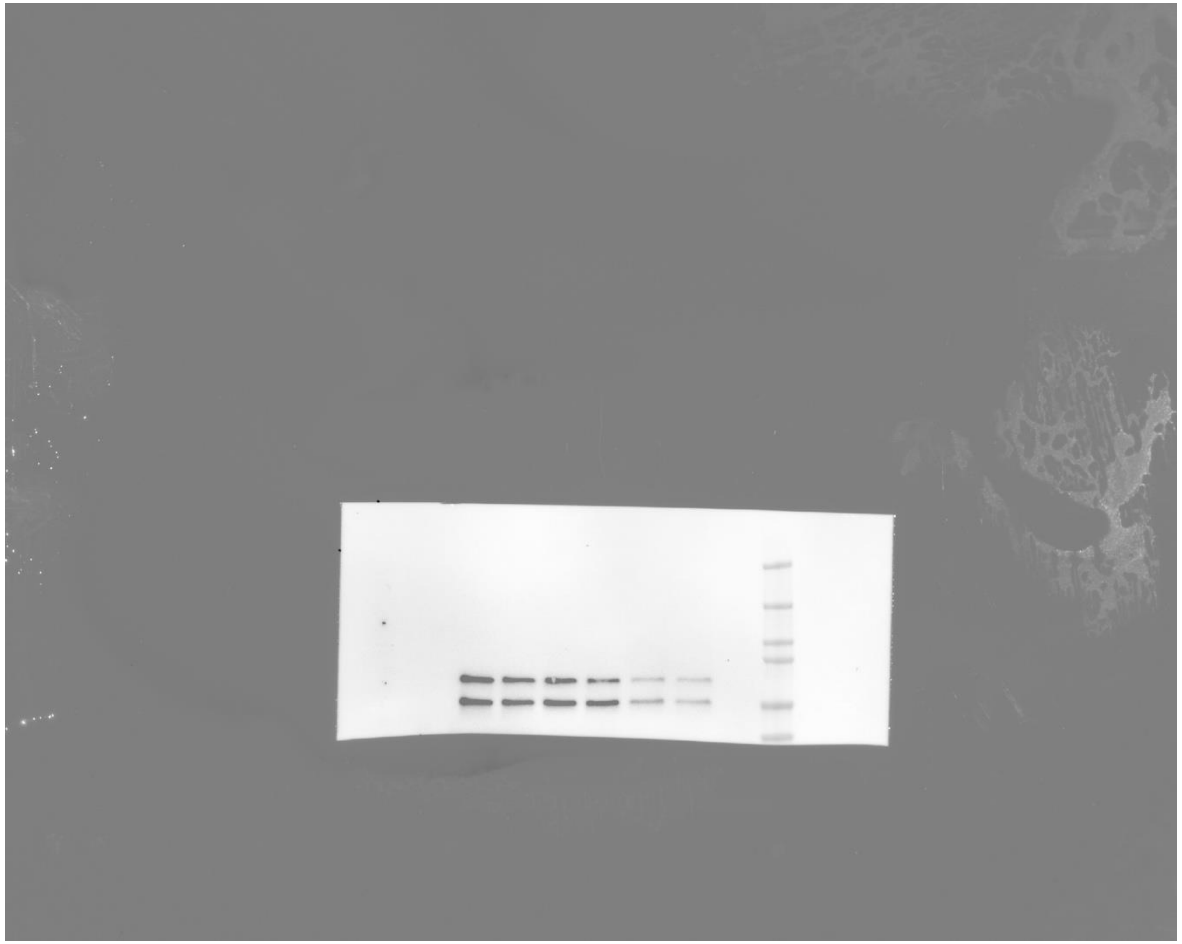

Anti-RT
Detection of RT heterodimer p66/p51

Figure 5B IN CC

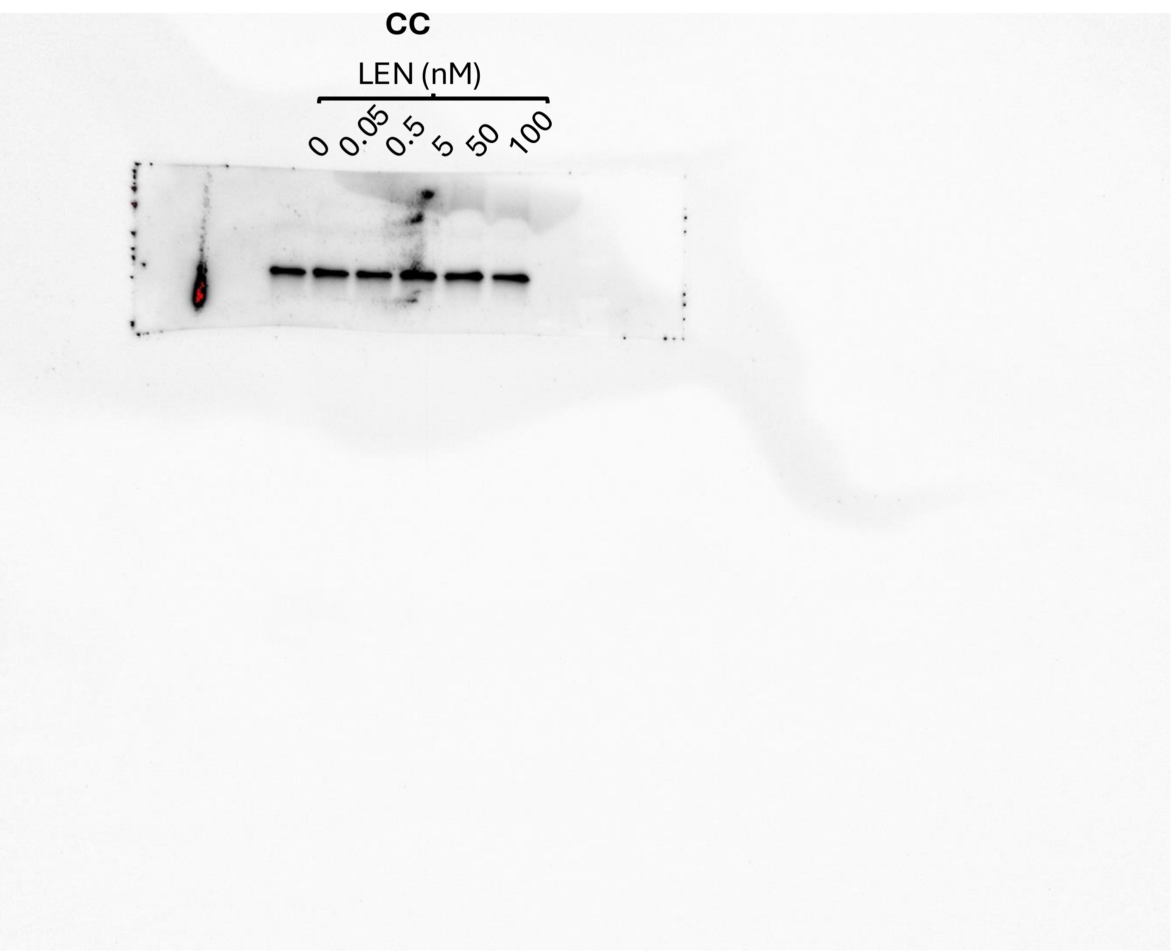

Anti-IN
Detection of IN/p32

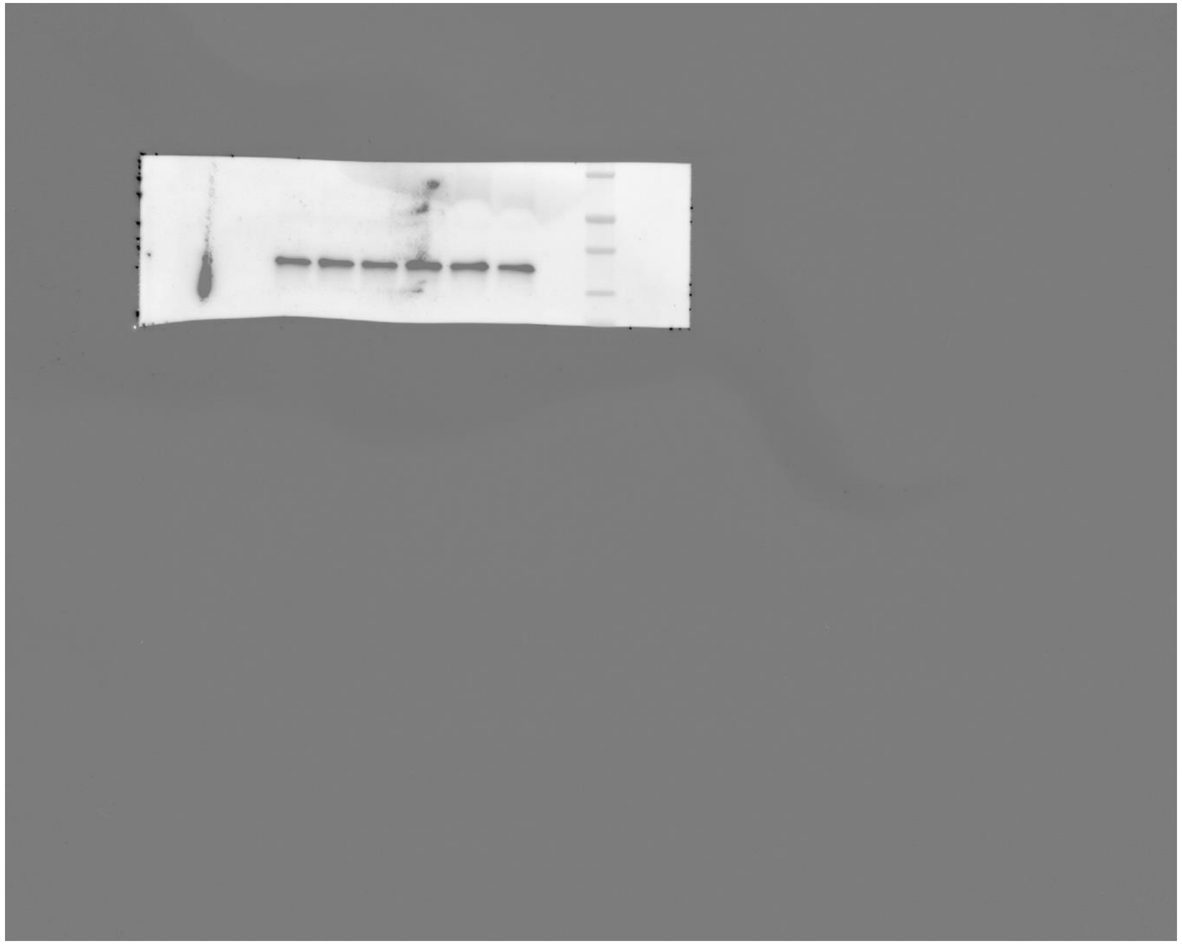

Figure 6A CA

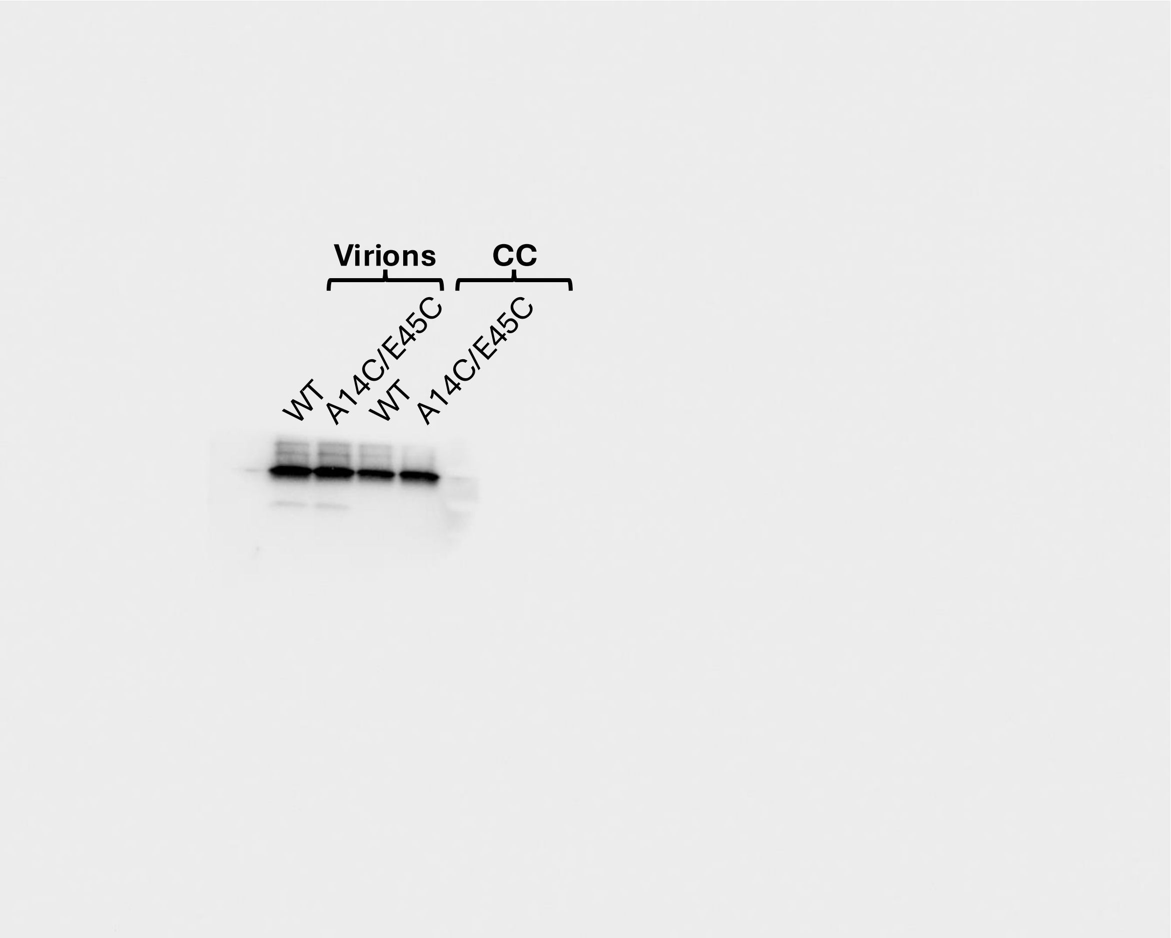

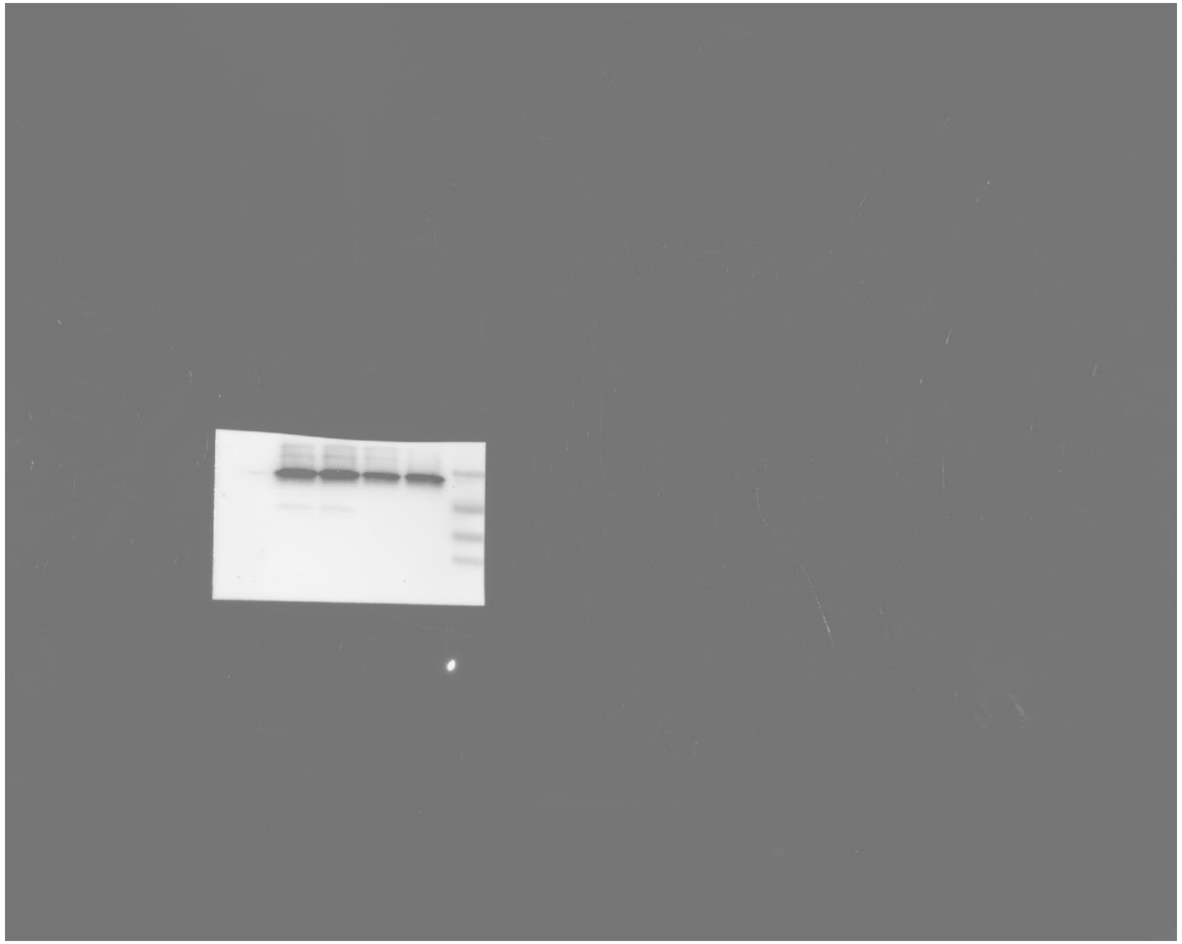

Anti-CA
Detection of CA/p24

Figure 6A NC

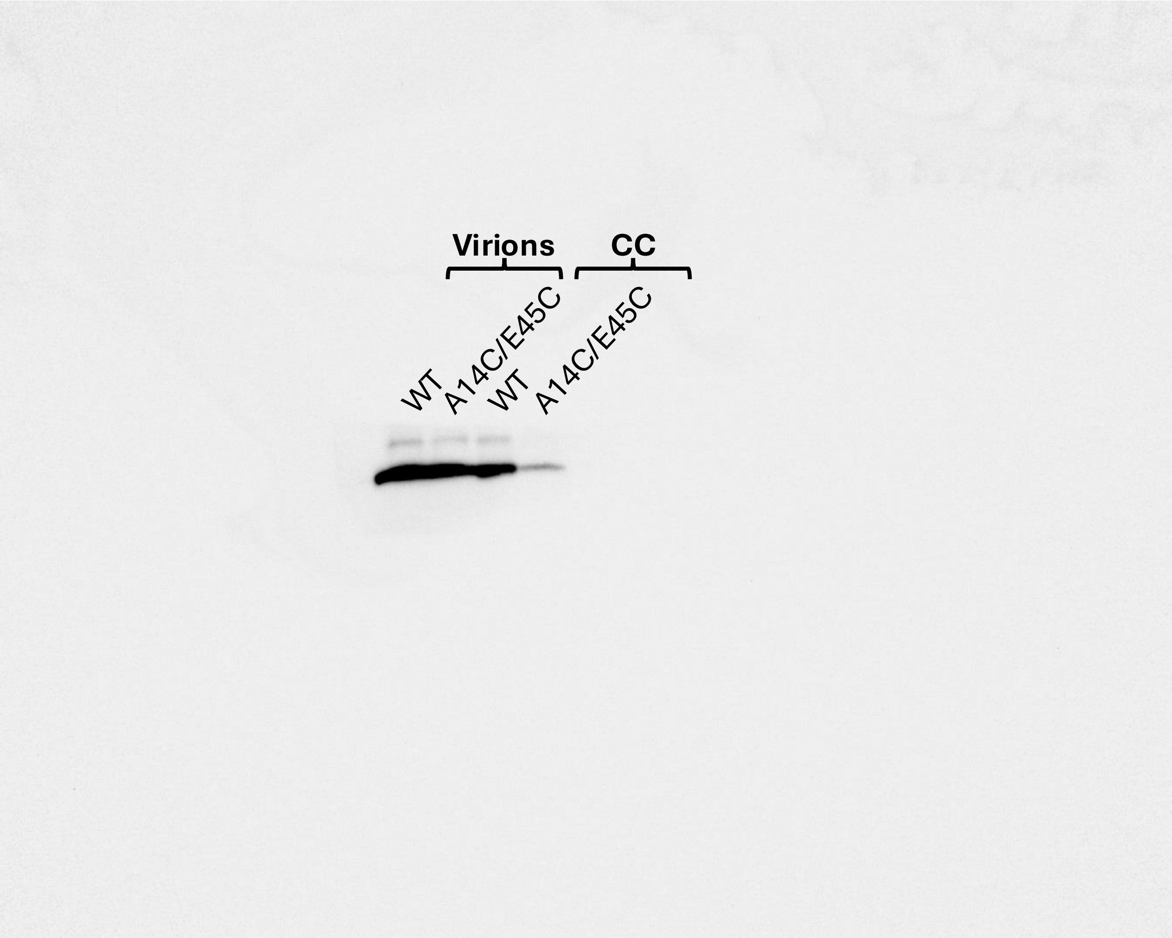

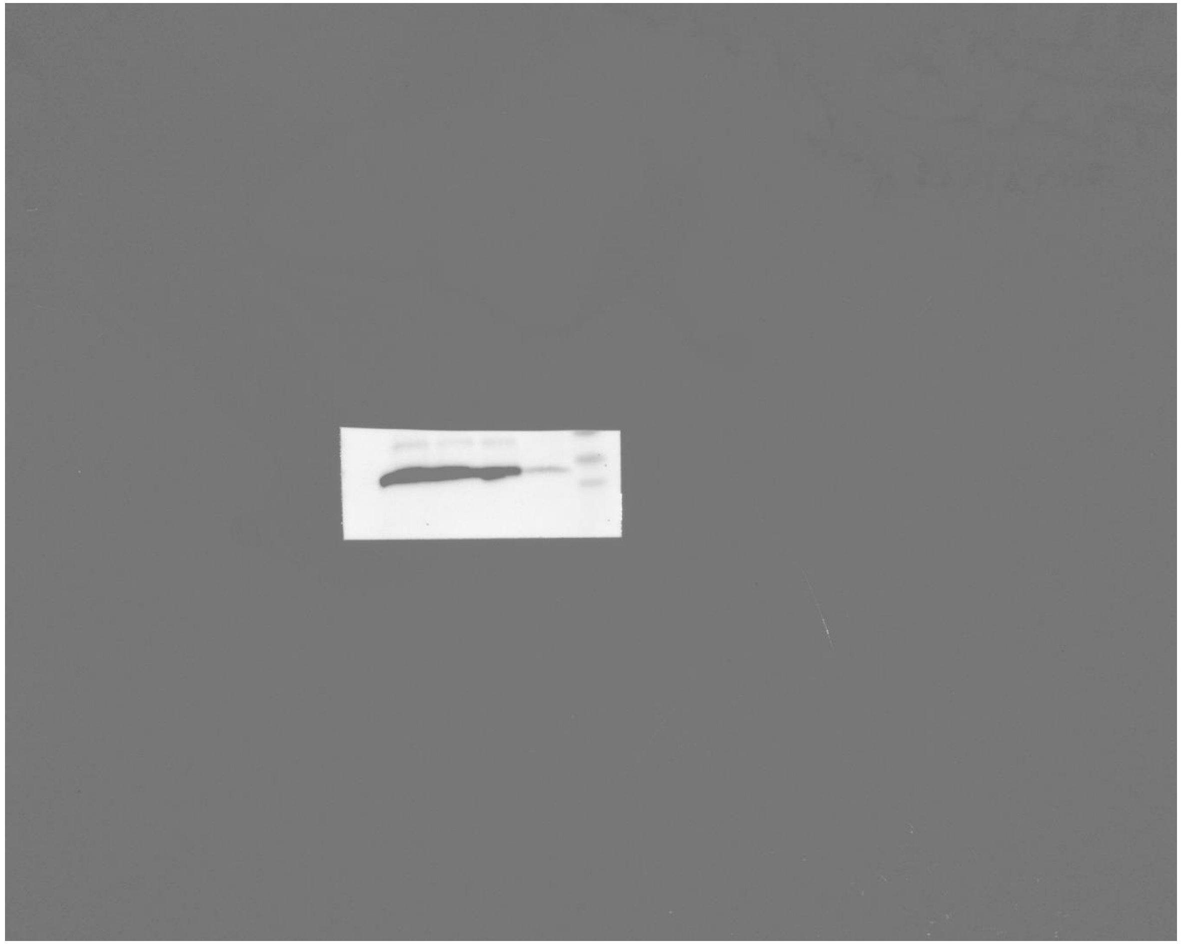

Anti-NC
Detection of NC

Figure 6A NC – different exposures

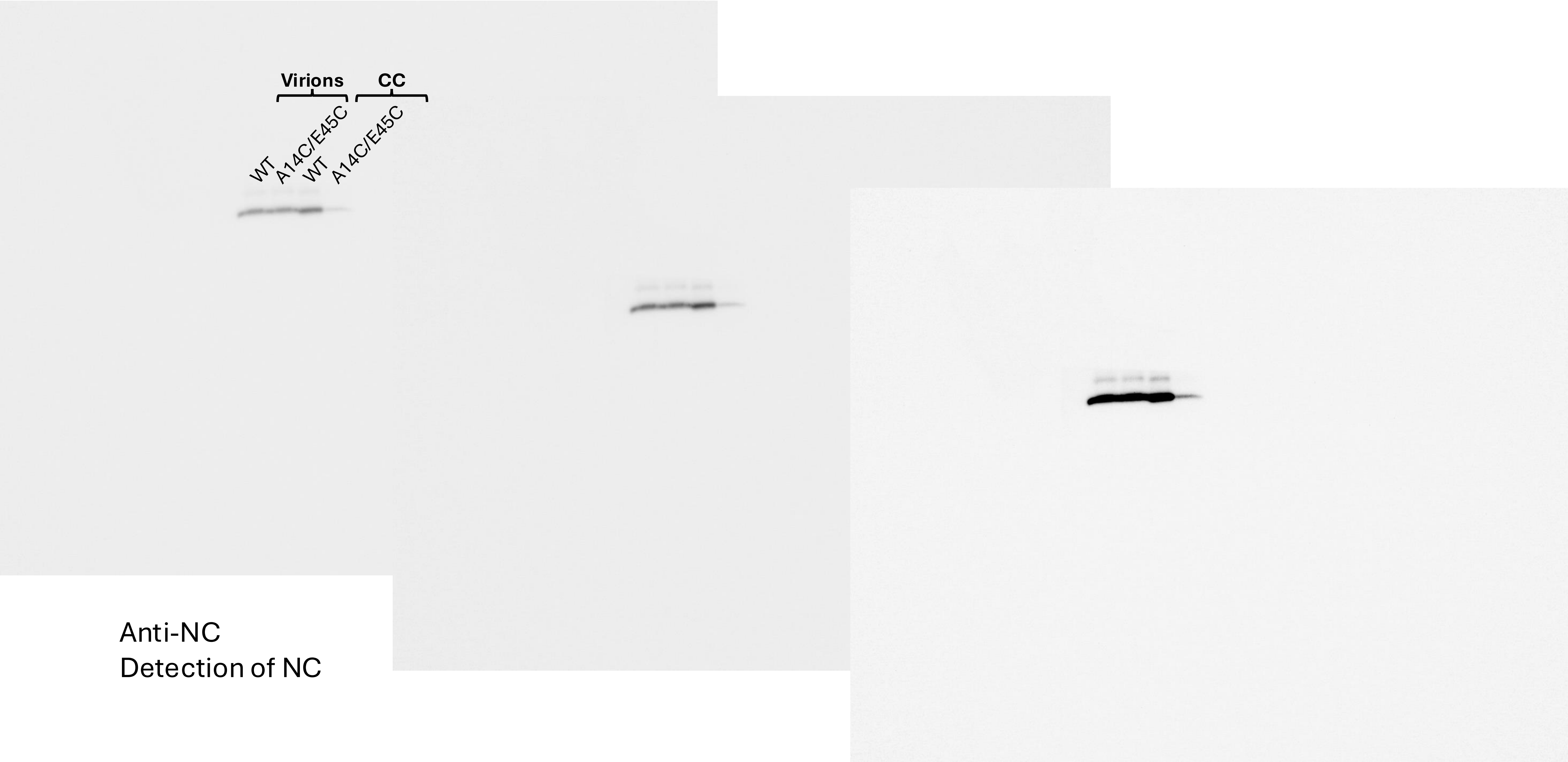

Anti-NC
Detection of NC

Another NC – different exposures

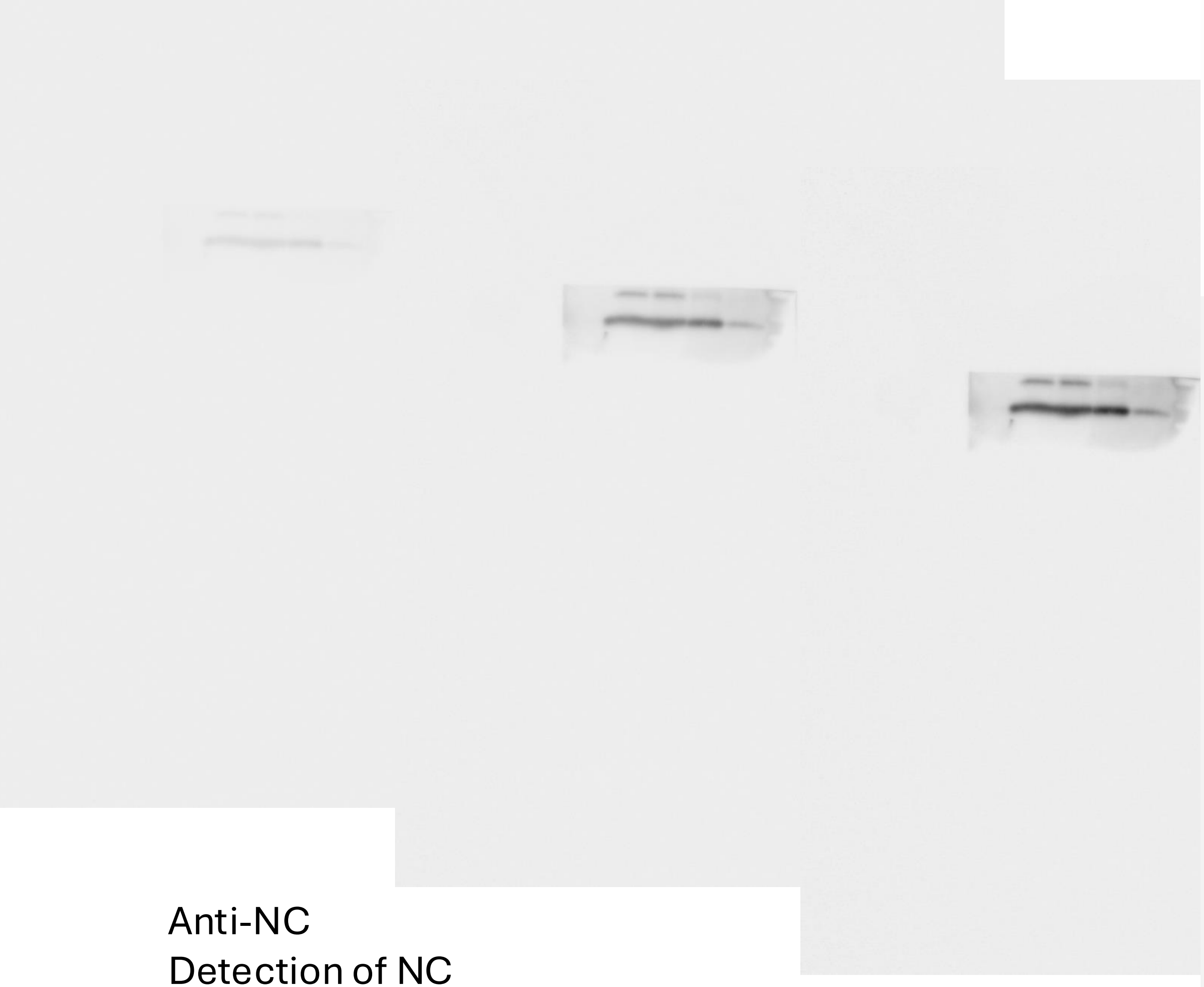

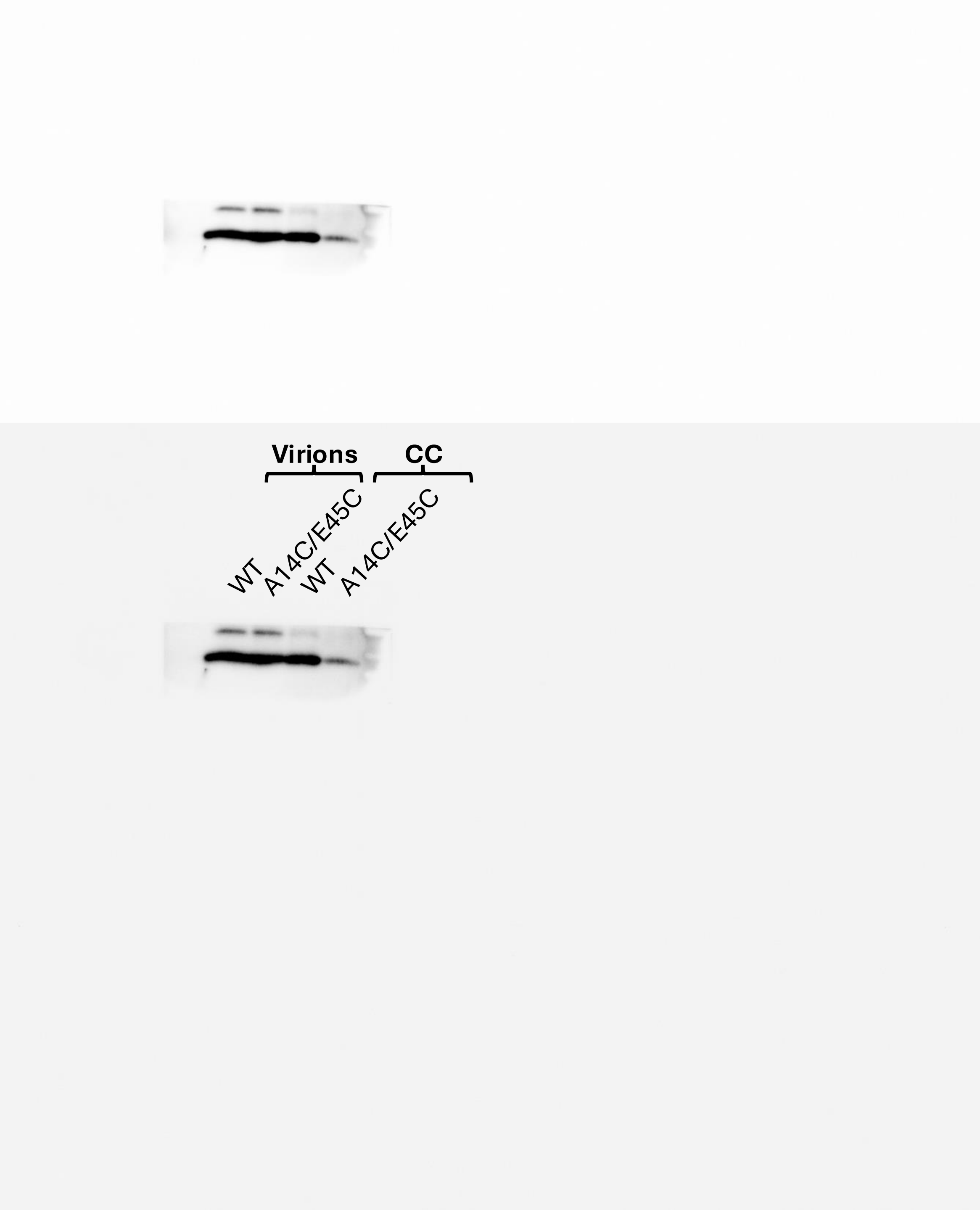

Anti-NC
Detection of NC

Figure 6A RT

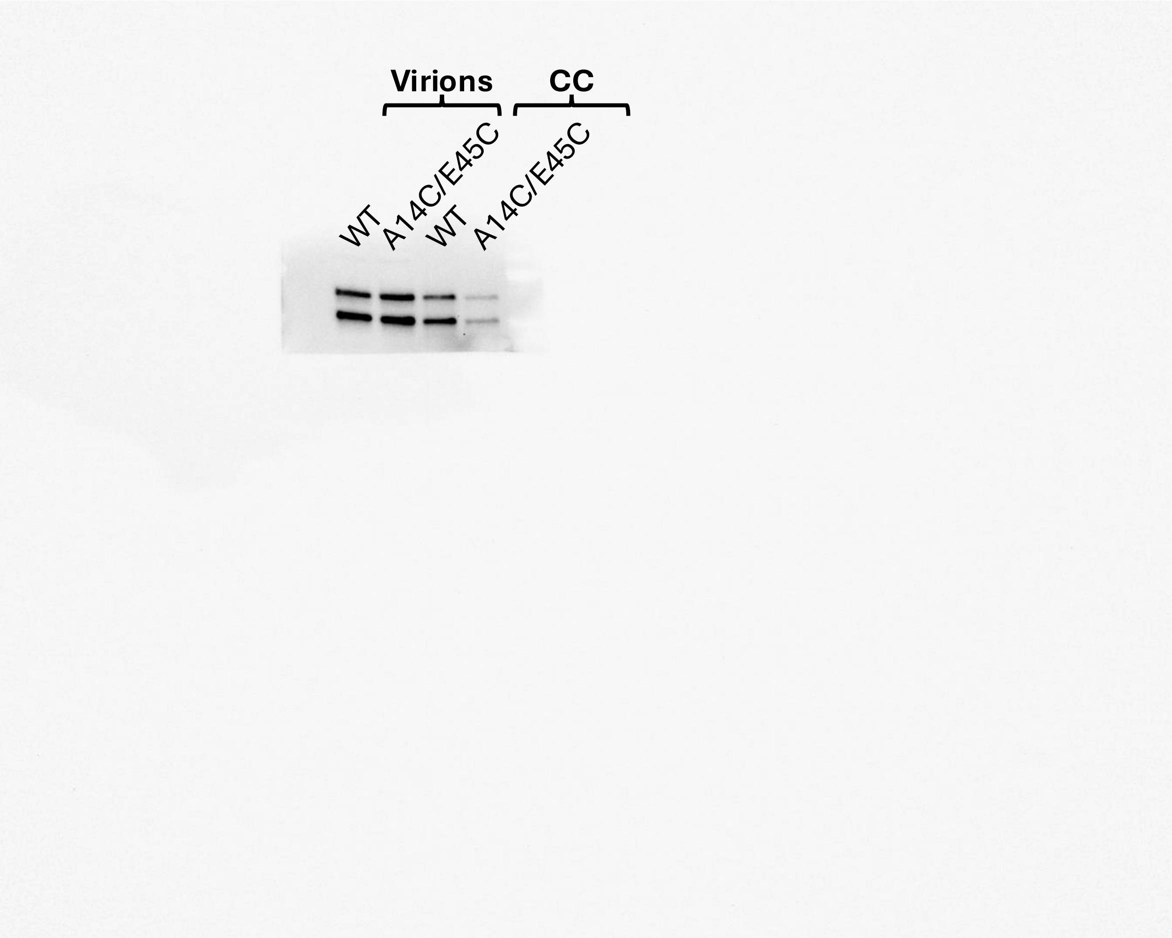

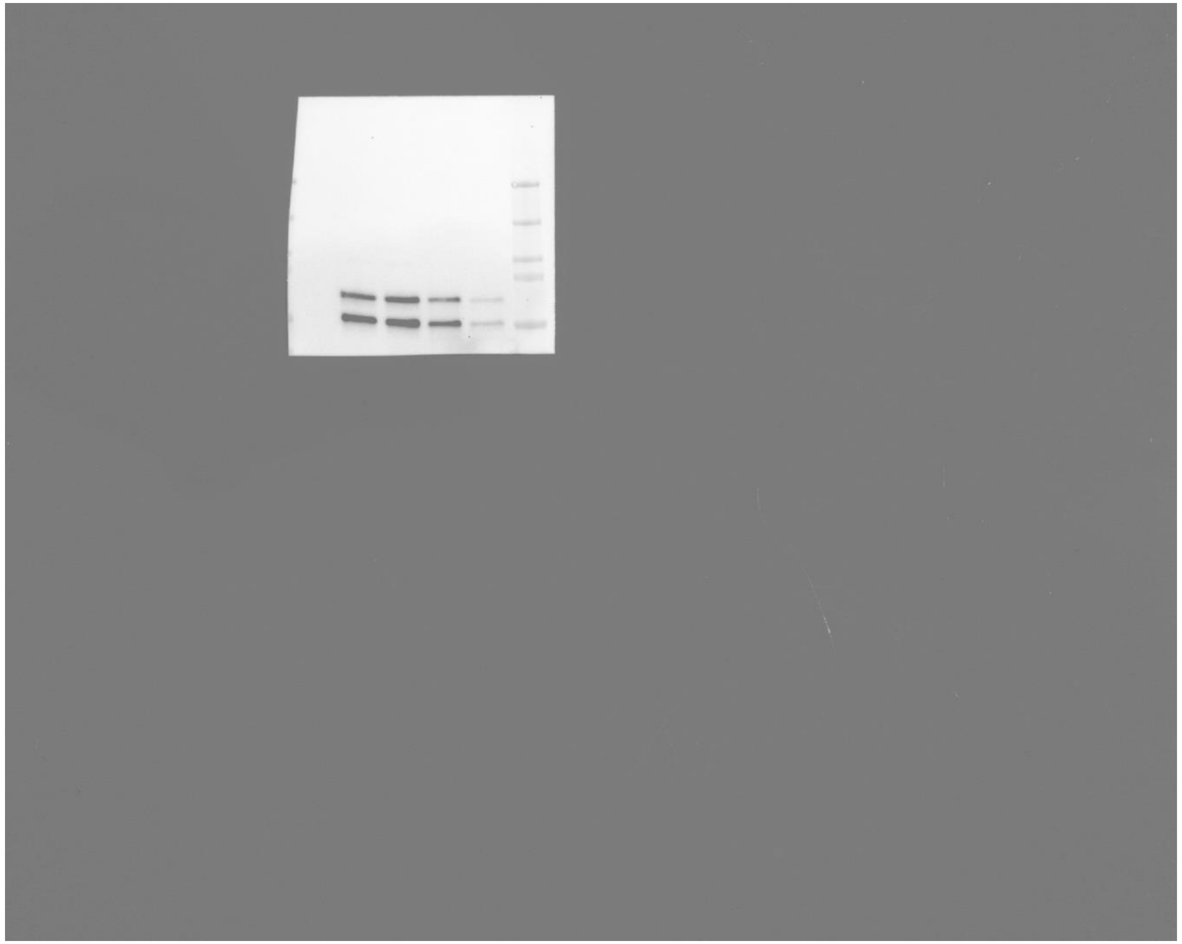

Anti-RT
Detection of RT heterodimer p66/p51

Figure 6A IN

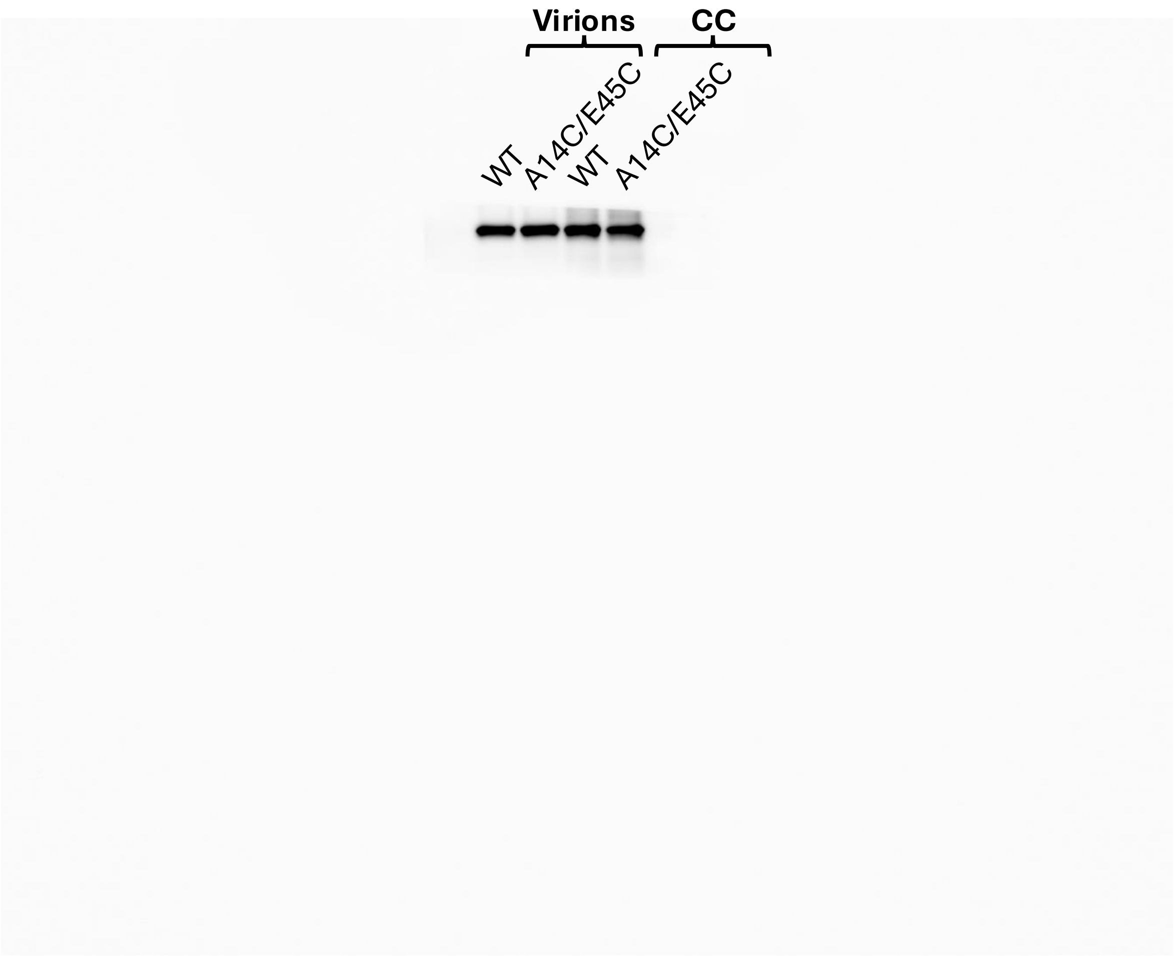

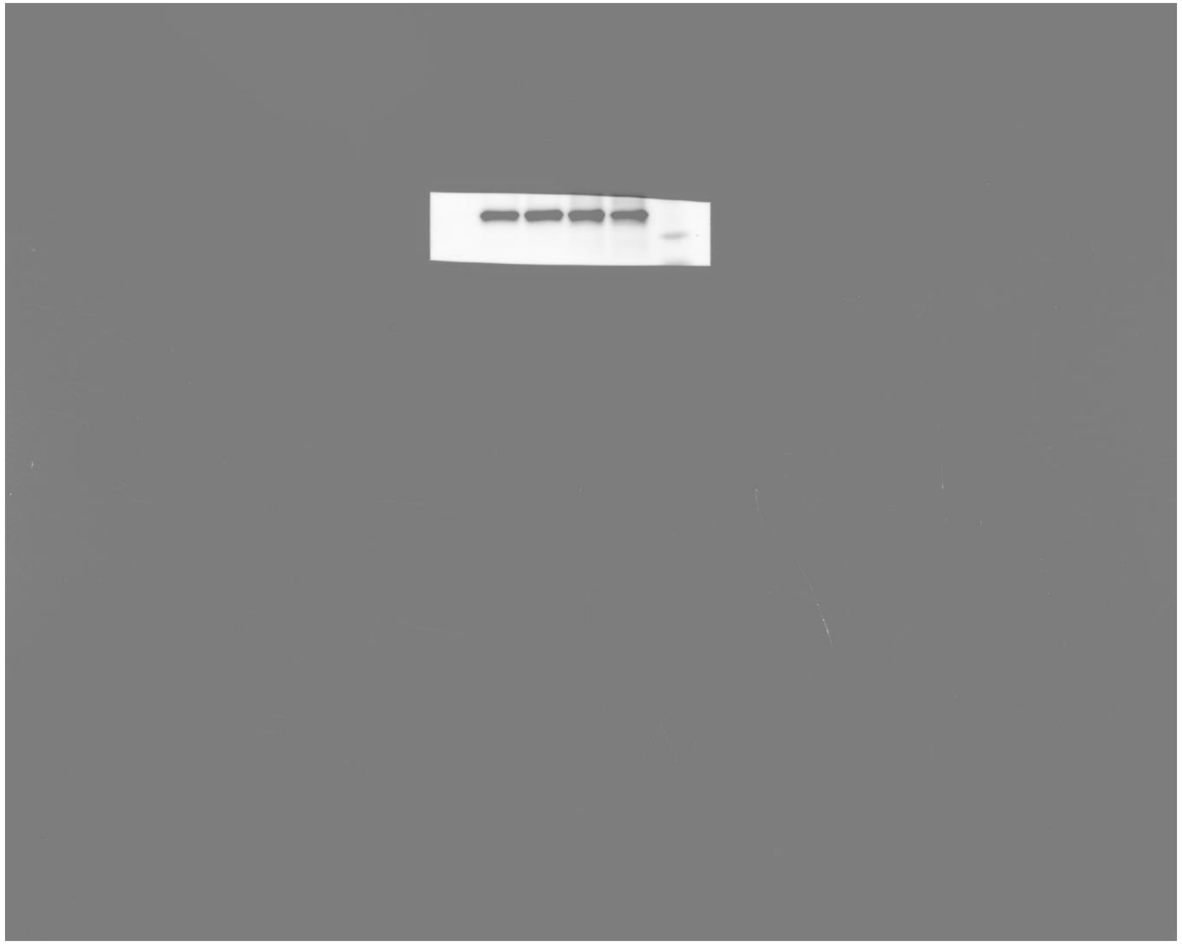

Anti-IN
Detection of IN/p32

Suppl. Figure 1C

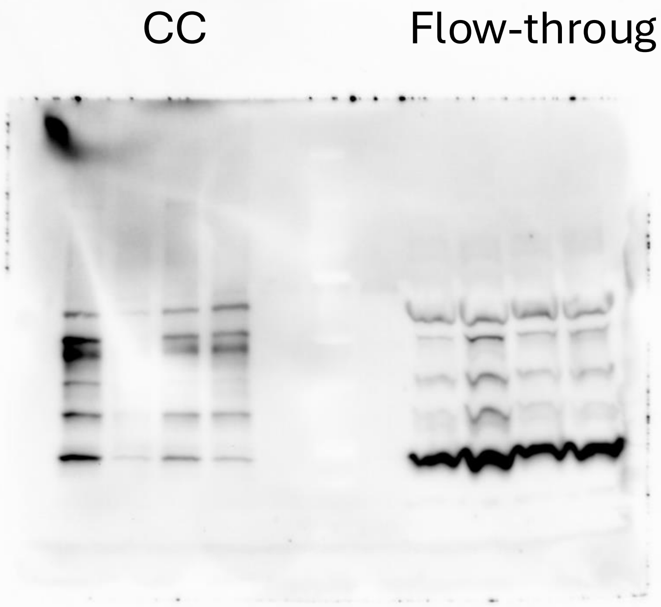

Validation of CC with different
CDR-binding antibodies
Using anti-HIV-IgG serum

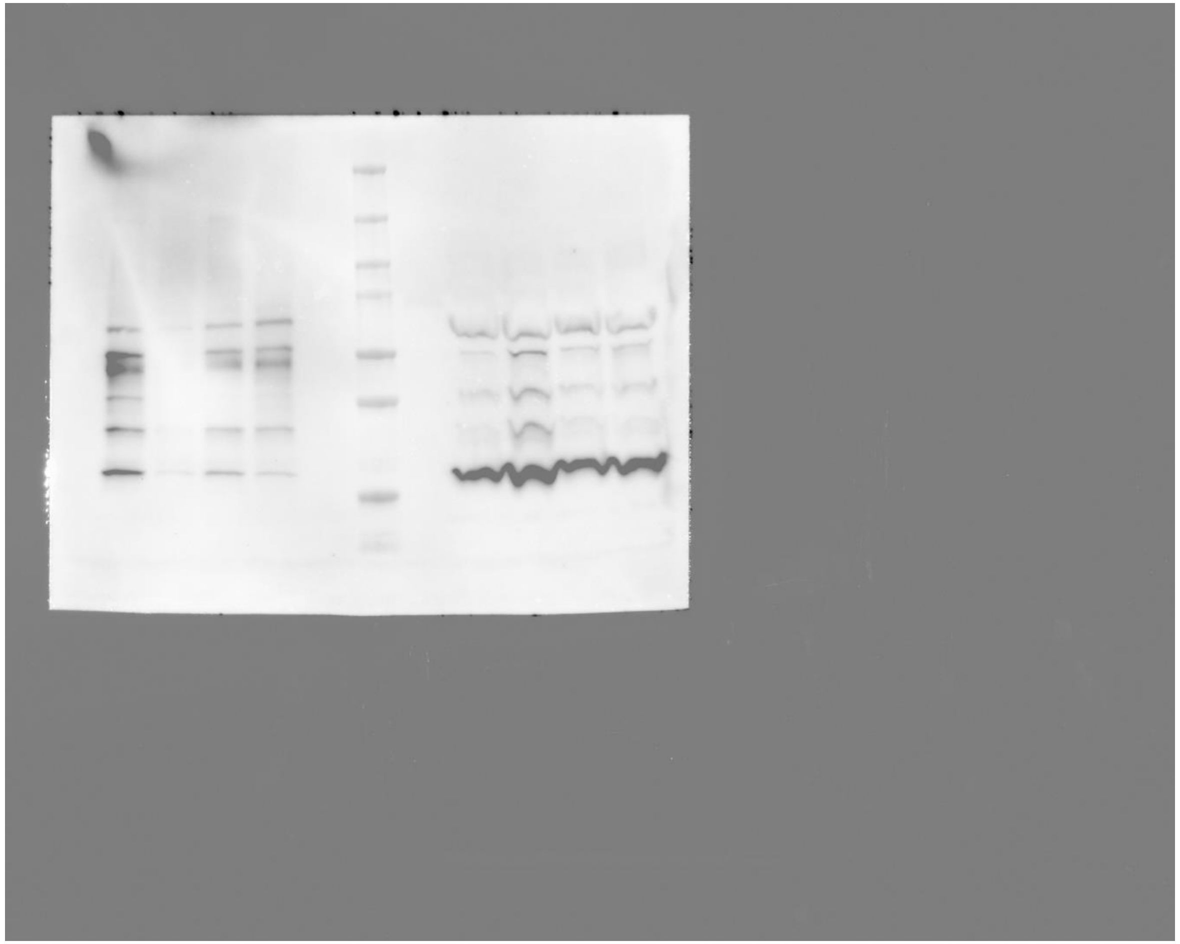

Suppl. Figure 1D

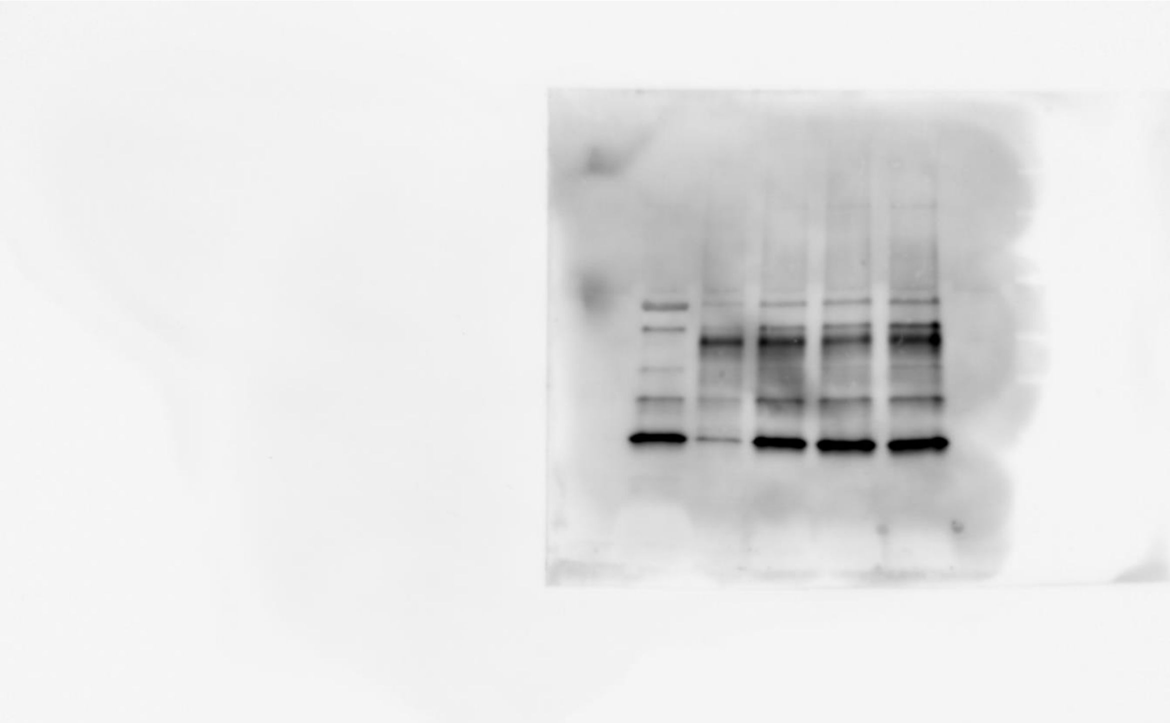

Validation of CC with volumes of antibodies
Using anti-HIV-IgG serum

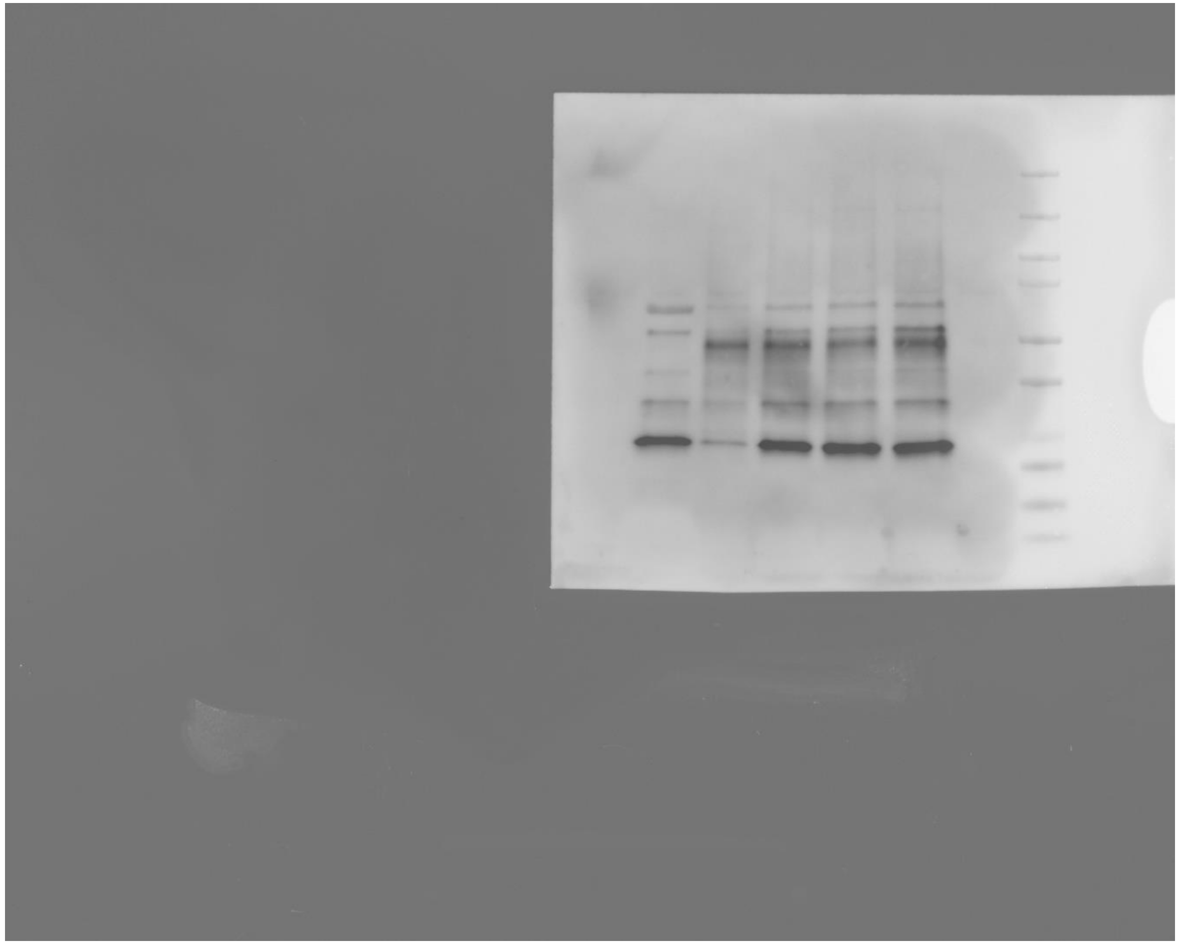

A high contrast blot in the paper

Suppl. Figure 1E

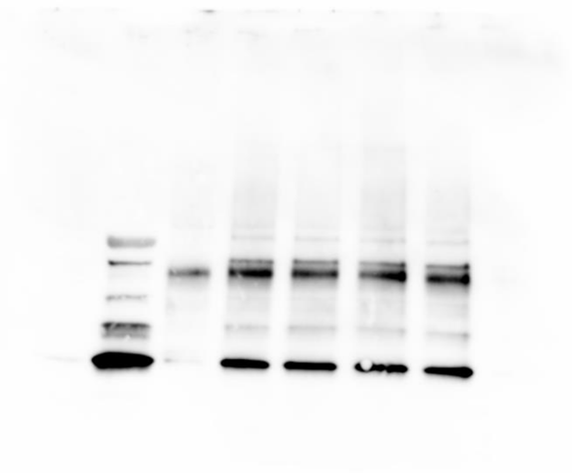

Validation of CC with different volume of CDR-coated beads
Using anti-HIV-IgG serum

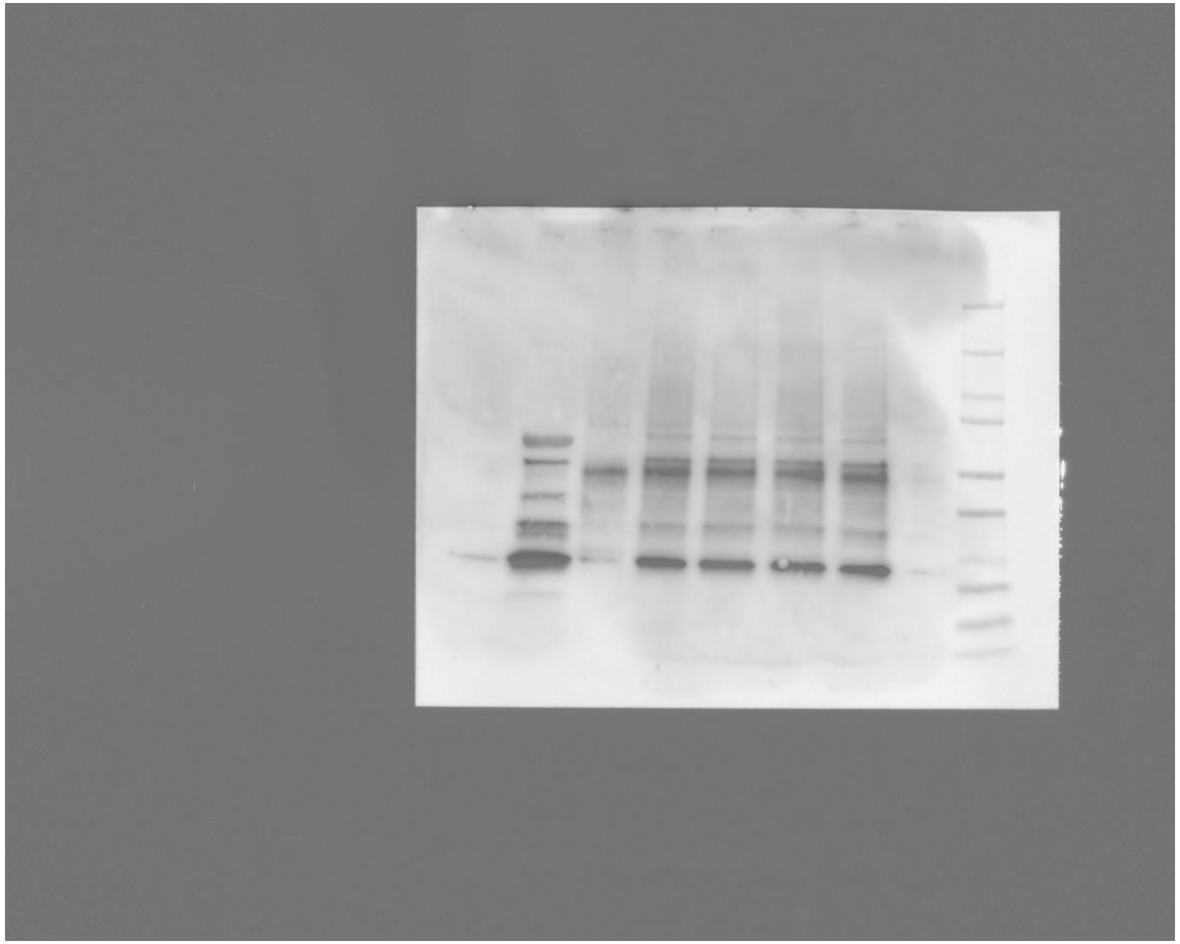

Suppl. Figure 2A

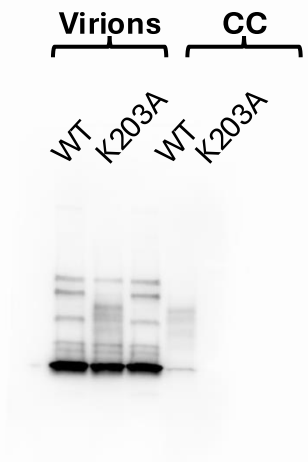

Probed with anti-HIV-IgG serum

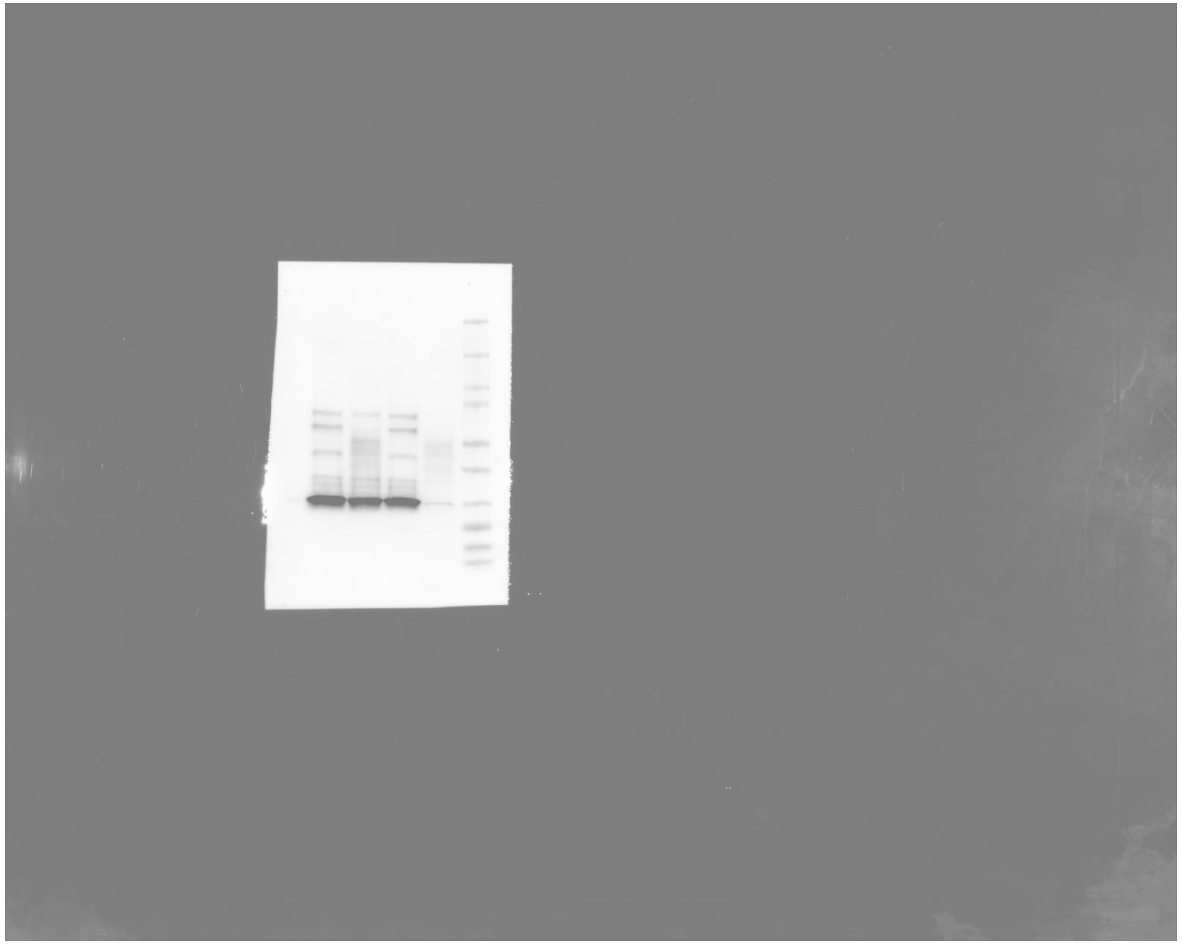

Suppl. Figure 2B

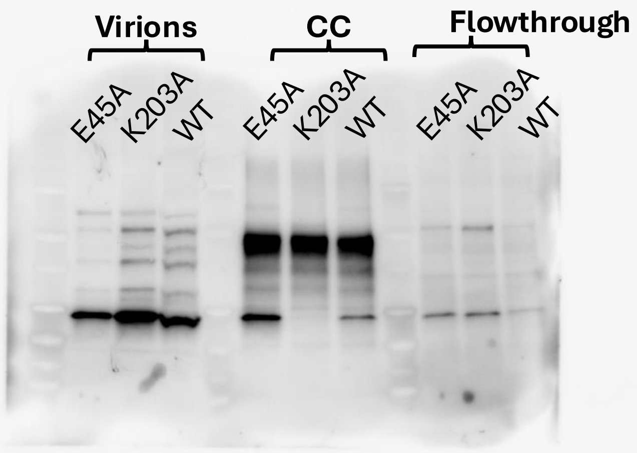

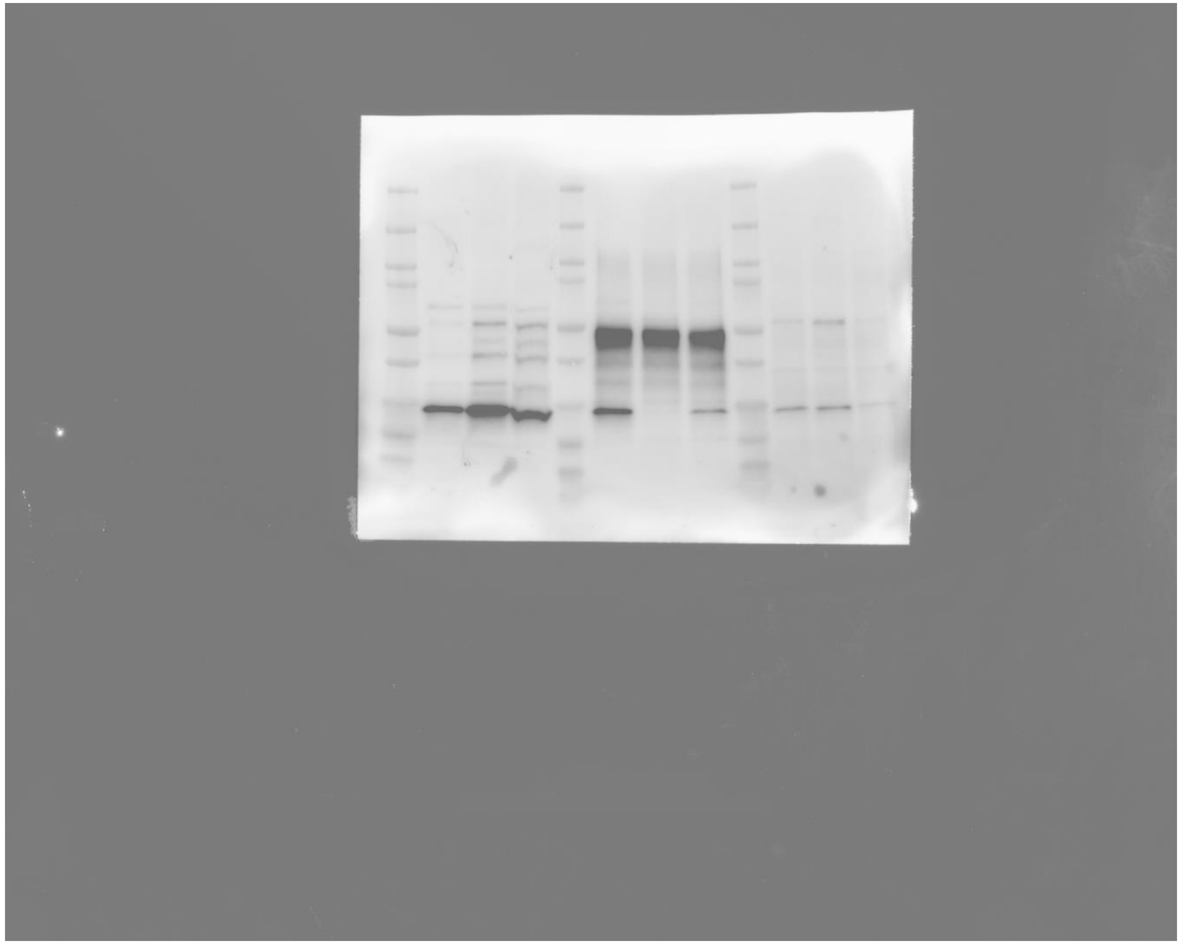

Fate of capsid assay
Probed with anti-HIV-IgG serum

Suppl. Figure 4A

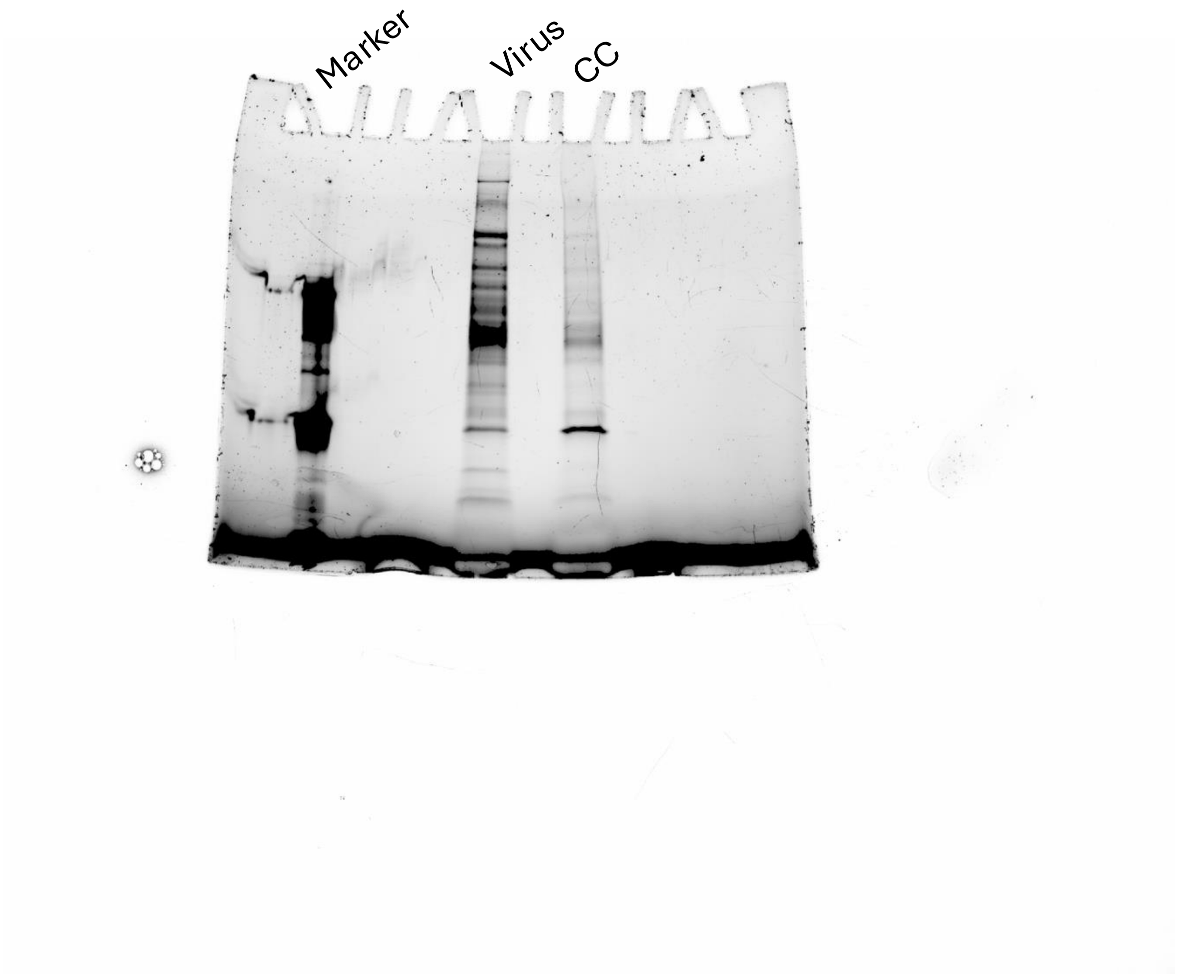

Low exposure

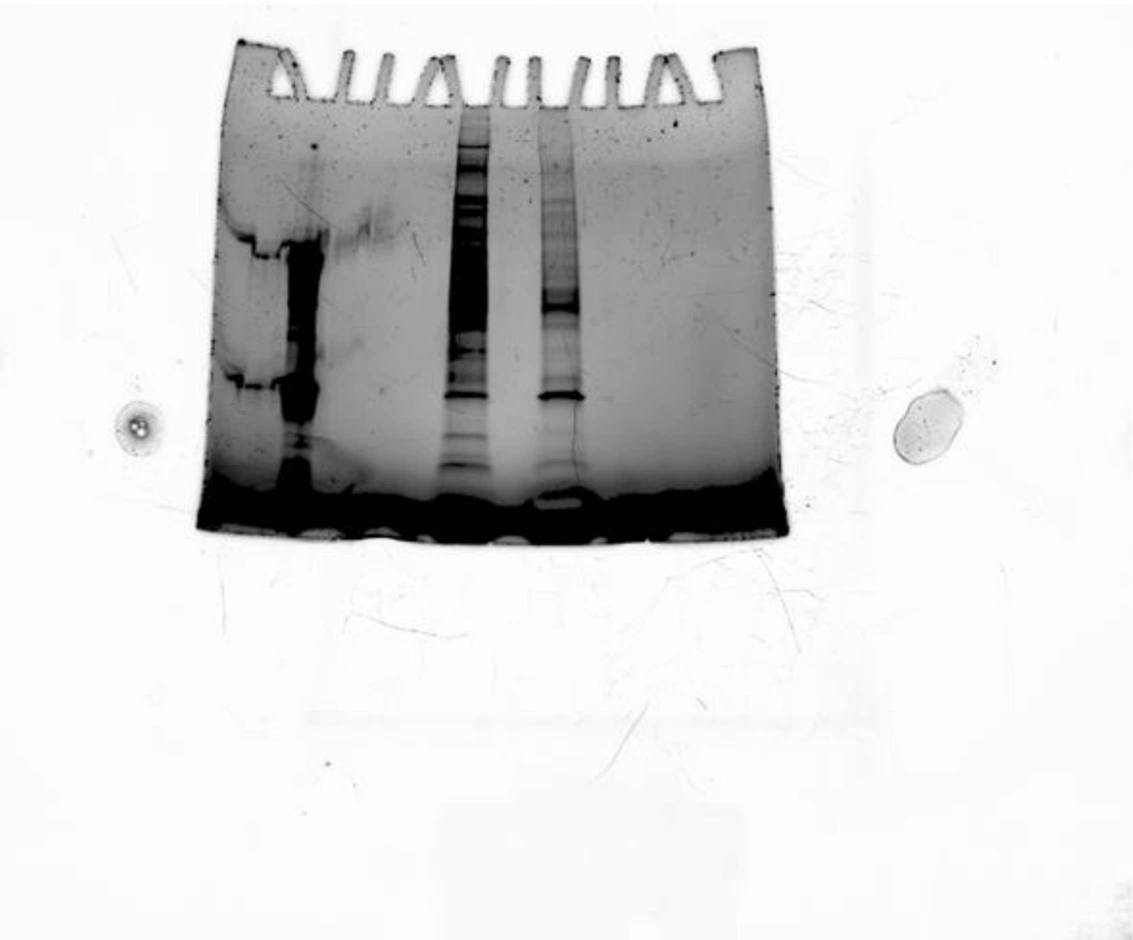

High exposure
